# Concurrent control of natural and robotic limbs through a tactile-encoded brain-computer interface

**DOI:** 10.1038/s41467-026-75213-3

**Published:** 2026-07-02

**Authors:** Tianyu Jia, Xingchen Yang, Ciaran McGeady, Yifeng Li, Jinzhi Lin, Kit San Ho, Feiyu Pan, Linhong Ji, Chong Li, Dario Farina

**Affiliations:** 1 Department of Bioengineering, Imperial College London, London, UK; 2 Department of Mechanical Engineering, Tsinghua University, Beijing, China; 3 School of Automation, Southeast University, Nanjing, China; 4 School of Biomedical Engineering, Tsinghua University, Beijing, China

**Keywords:** Biotechnology, Biological techniques, Neuroscience, Biomedical engineering

## Abstract

Brain-computer interfaces (BCIs) promise to extend human movement capabilities by enabling direct neural control of supernumerary effectors, yet integrating augmented commands with multiple degrees of freedom without disrupting natural movement remains a key challenge. Here, we propose a tactile-encoded BCI that leverages sensory afferents through a tactile-evoked P300 paradigm, allowing reliable decoding of supernumerary motor intentions even when superimposed with voluntary actions. The interface was evaluated in a multi-day experiment comprising a single motor recognition task to validate baseline BCI performance and a dual-task paradigm to assess the potential influence between the BCI and natural human movement. The interface achieved real-time and reliable decoding of four supernumerary degrees of freedom, with significant performance improvements after three days of training. After training, performance did not differ significantly between the single-task and dual-task conditions, and natural movement remained unimpaired during concurrent supernumerary control. Lastly, the interface was deployed in a movement augmentation task, demonstrating its ability to command two supernumerary robotic arms for functional assistance during bimanual tasks. These results establish a neural interface paradigm for movement augmentation through stimulation of sensory afferents, expanding motor degrees of freedom without impairing natural movement.

## Abstract (web summary)

Disentangling superimposed motor intentions remains a challenge for movement augmentation. Here, the authors develop a tactile-encoded brain-computer interface that enables concurrent control of natural and supernumerary degrees of freedom.

## Introduction

Humans interact with their surroundings with remarkable dexterity and efficiency. Recent advances in robotics and neural interfaces hold the potential to increase these capabilities, enhancing human movement beyond its natural limits. Movement augmentation aims to increase the mechanical degrees of freedom (DoFs) an individual can exert over their surroundings^1^, allowing movement tasks to be performed more efficiently or enable actions otherwise impossible with natural limbs alone, such as trimanual manipulation with a third arm^2^. A central challenge, however, lies in achieving practical control of supernumerary effectors without compromising natural movement. Here, we present a non-invasive method for supernumerary control that preserves natural movement while augmenting human motor capability.

Current strategies for augmenting DoFs often rely on augmentation by transfer, in which control of supernumerary effectors is derived from the function of an existing body part, typically one that is task-irrelevant^1,3,4^. For example, a supernumerary thumb has been controlled using a sensor on the toe^5^. While this approach can increase DoFs for a specific task, it does so at the cost of constraining DoFs elsewhere in the body. By contrast, augmentation by extension aims to increase the body’s total number of DoFs by decoding body signals that are independent of overt movement and whose modulation does not interfere with ongoing motor tasks^1^. Invasive cortical recording techniques have shown promise for cursor control during simultaneous independent natural movement^6,7^. While effective, non-invasive approaches such as high-density surface electromyography (EMG) and electroencephalography (EEG) may offer a safer, cost-effective, and widely accessible alternative. For instance, Lee et al. investigated whether muscle activation patterns within the wrist’s muscle-to-force null-space can be voluntarily modulated to provide independent control signals^8^. Their findings indicate that this null-space activity affords at most a single, low-fidelity control dimension that remains tightly coupled to natural wrist movements, limiting its suitability for reliable control. High-frequency inputs to motor neurons, reflected in EMG and largely independent of force production, represent an alternative control source^9^. While these signals have predominantly been modulated using external stimuli^10^, some evidence suggests they can also be influenced endogenously. For example, Ofner et al. demonstrated modulation of high-frequency components in motor neuron activities through motor imagery and even imagery of non-motor tasks such as mental arithmetic^11^. However, the resulting modulation was weak and inconsistent. Consequently, robust strategies for voluntary modulation remain lacking, and the practical applicability of EMG signals for supernumerary effector control remains uncertain.

EEG for control of external devices has been extensively studied within the field of brain-computer interfaces (BCIs) for neurorehabilitation. While rehabilitation primarily aims to restore the lost DoFs following neurological damage^12^, human movement augmentation pursues the enhancement of unimpaired abilities. Despite these differing objectives, both domains share fundamental principles in interpreting neural activity. Sensorimotor rhythms (SMRs) from EEG have been widely used for user command recognition in individuals with severe motor disabilities^13,14^. In paradigms involving ongoing movements, SMR activity is typically modulated using motor imagery (MI) of a task-irrelevant body part^15,16^. Similarly, Xie et al. employed classification of steady-state visual evoked potentials (SSVEPs) from occipital EEG channels to infer user intent and trigger pre-programmed commands for a robotic third arm^17^. While compelling, these approaches highlight the central challenge of augmentation: the redistribution of cognitive resources or detriment of natural motor function. More closely aligned with the augmentation by extension, Penaloza and Nishio demonstrated a SMR-based BCI, where able-bodied participants activated a robotic arm through grasping MI while performing a bimanual ball-balancing task concurrently^2^. However, their system functioned as a single discrete brain switch, and the extension to more DoFs faces significant challenges. While MI offers a reliable source of multidimensional neural signals that can be internally modulated, its application in human augmentation is impractical as it requires deciphering supernumerary control intentions that are superimposed on the neural activity associated with natural movement. Additionally, MI that is contradictory to ongoing movement may be counterintuitive for the user. Event-related potentials (ERPs), particularly the P300 signal, have also been extensively employed in BCI applications. The P300 is characterized in EEG by a positive deflection with an amplitude of ~5–10 μV, around 200–500 ms after a rare, task-relevant stimulus. The classic Farwell and Donchin “P300 Speller” enables users to communicate sequences of letters to a computer by exploiting this response^18,19^. In this paradigm, attending to an infrequent target flash elicits a P300 that can be decoded to infer the intended letter, typically achieving accuracies above 80% at around 7–8 characters per minute^20,21^. The P300 is particularly appealing for human augmentation as it offers a high number of DoFs with little training time and high accuracy; for example, a 6×6 matrix speller enables 36 distinct selectable options. However, the visual P300 is impractical for movement augmentation as it occupies the user’s entire visual attention. A vibrotactile modification of the classic visual paradigm holds promise for augmentation by extension. For instance, Li et al. delivered bursts of electrical stimulation to the fingers, analogous to the flashes in the matrix speller, eliciting sensory-evoked P300 responses for potential clinical applications in which the visual system is impaired^22^. Auditory implementations have also been proposed, but they typically achieve lower accuracies and, for movement augmentation, are associated with increased subjective workload^23^. We hypothesise that a tactile-encoded P300 could serve as an effective signal for discrete control of artificial supernumerary DoFs for human movement augmentation. This signal is robust, quick to calibrate, and can be decoded without interfering with natural movements or overloading the visual system.

**Fig. 1:**
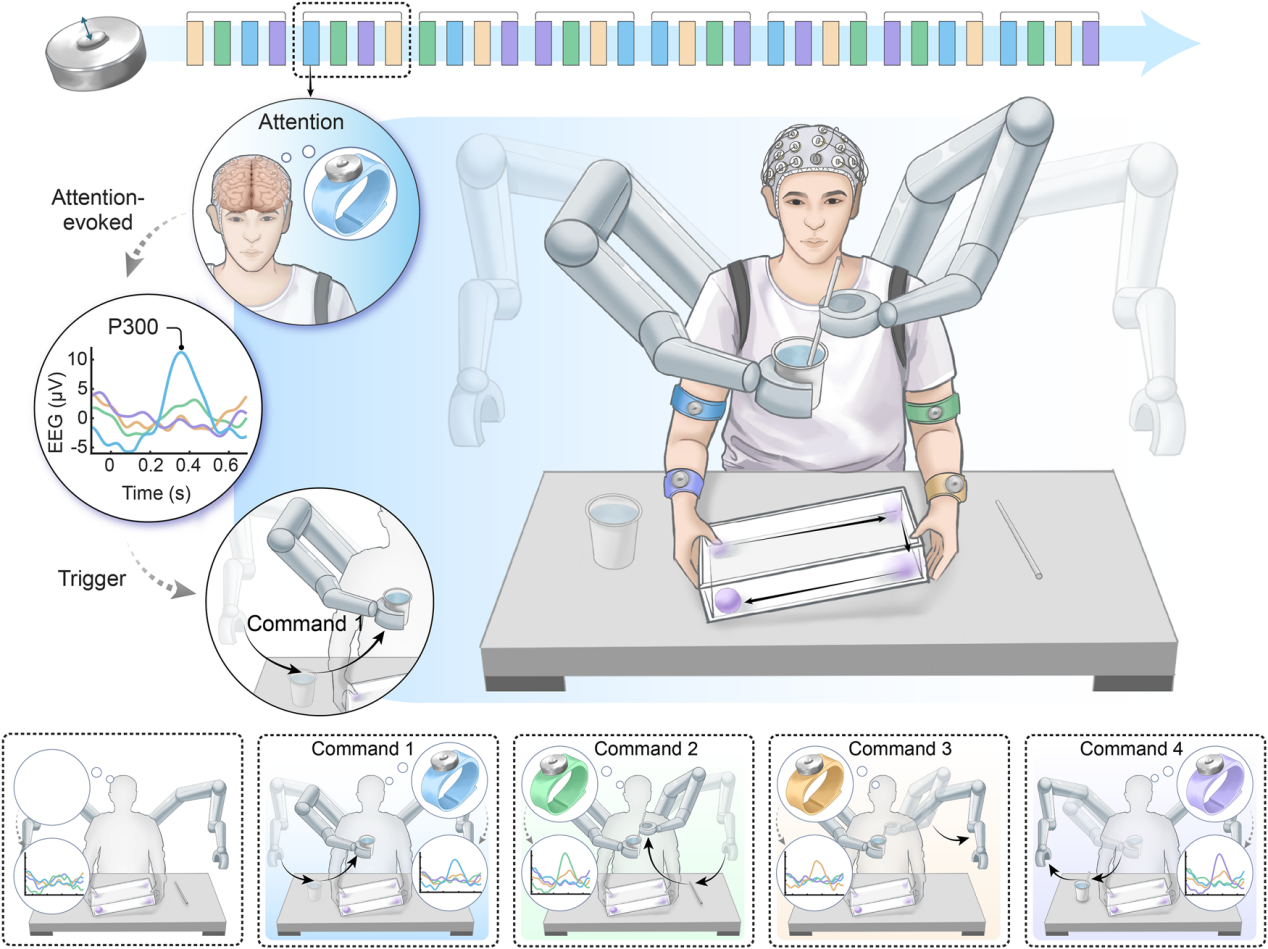
Experimental setup for tactile-encoded brain-computer interface control of supernumerary robotic arms. Four tactile vibrators were attached bilaterally to the upper and lower arms, each delivering 200-ms bursts of vibration with 200-ms interstimulus intervals in a pseudorandom order. Each vibrator corresponded to one of four selectable control states, with symmetric placement to avoid proprioceptive bias. Attended stimuli evoked P300 responses in the EEG, which were decoded online to trigger corresponding robotic commands. A transparent tray was used for the ball-balancing task in the dual-task condition.

To test our hypothesis, we investigated whether a tactile-encoded BCI can enable concurrent control of natural and robotic limbs for movement augmentation. EEG was recorded from ten able-bodied participants while engaged in our tactile-encoded BCI experiment. The BCI leveraged sensory afferents using an oddball paradigm where users selectively focused on vibrotactile target stimuli while ignoring non-targets (Fig. 1). Vibrotactile stimuli were delivered by four tactile vibrators, each corresponding to one BCI command. The P300 response, evoked by the participant’s attention to the target stimuli, was detected and used for BCI control. To evaluate the effectiveness of the tactile-encoded BCI for movement augmentation, participants completed three tests: (i) a baseline ball-balancing task to quantify bimanual motor control; (ii) an online BCI test to evaluate the performance of the tactile-encoded BCI for supernumerary command recognition (single-task); and (iii) a dual task combining BCI command recognition with the ball-balancing task to assess the potential interactions between the tactile-encoded BCI and natural movement ability (dual-task). To evaluate potential training effects on BCI performance, the experimental protocol was repeated on three separate days. Lastly, to demonstrate the feasibility of the proposed movement augmentation framework, we designed a functional test where participants controlled two supernumerary robotic arms using the tactile-encoded BCI to complete four-limb coordination tasks (two real arms and two supernumerary robotic arms, Supplementary Fig. S1, Supplementary Movies S1–S4). Overall, this study investigated (i) whether a tactile-encoded BCI can be utilised for user command recognition and DoF augmentation, (ii) whether the performance of the tactile-encoded BCI improves with training, (iii) whether the tactile-encoded BCI interferes with natural movement, and (iv) the performance of a tactile-encoded BCI used for functional movement augmentation tasks.

## Results

### Tactile vibrator enables stable P300 for user command recognition

**Fig. 2:**
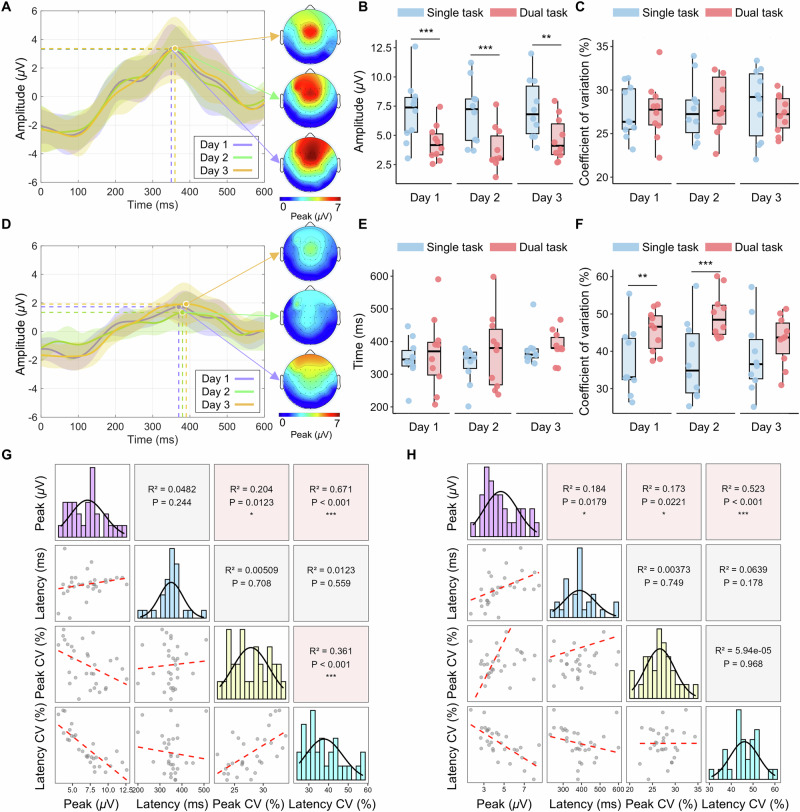
Evoked P300 underlying the tactile-encoded brain-computer interface tasks over three days of experiment across 10 participants. Grand-averaged P300 of Cz for **A** single-task and **D** dual-task conditions, with topographic maps illustrating the P300 peak distribution. The solid lines represent the mean amplitude, and the shaded boundary represents the standard deviation across participants. A one-way repeated-measures ANOVA with a Greenhouse-Geisser correction was applied with main effects of training on P300 peak and latency, followed by post hoc analysis with Bonferroni correction. *P*-values: 0.74 (**A**, peak), 0.29 (**A**, latency), 0.14 (**D**, peak), 0.80 (**D**, latency). Boxplots of P300 (**B**) peak amplitude, (**C**) peak coefficient of variation (CV), (**E**) latency, and (**F**) latency CV (*n* = 10 participants). The centre lines indicate median values, box bounds represent the 25th and 75th percentiles, whiskers extend to the minimum and maximum values within 1.5 times the interquartile range. Comparisons between the single-task and dual-task conditions were conducted using two-tailed Wilcoxon signed-rank tests or two-tailed paired-sample t-tests with False Discovery Rate (FDR) correction for multiple comparisons, depending on whether the differences between paired observations met the assumption of normality. *P*-values: 7.40e−04 (**B**, Day 1), 7.02e-04 (**B**, Day 2), 0.0013 (**B**, Day 3), 0.87 (**C**, Day 1), 0.50 (**C**, Day 2), 0.50 (**C**, Day 3), 0.77 (**E**, Day 1), 0.73 (**E**, Day 2), 0.77 (**E**, Day 3), 0.0083 (**F**, Day 1), 5.46e-04 (**F**, Day 2), 0.12 (**F**, Day 3). Correlation matrices of P300 features for (**G**) single-task and (**H**) dual-task conditions, with each dot representing individual data points from each participant per day (*n* = 10 participants). One peak CV outlier was omitted from the figure but included in the statistical analysis. Pairwise associations were assessed using Pearson’s product-moment correlation tests (two-tailed). ***if *p* < 0.001, **if *p* < 0.01, *if *p* < 0.05.

The tactile-evoked P300 over three days of training was evaluated by the amplitude and latency of its positive peak (Fig. 2). For the single-BCI task (Fig. 2A), a one-way repeated-measures analysis of variance (ANOVA) revealed no significant main effect of training time on P300 amplitude (*F*_1.45,13.06_ = 0.21, *p* = 0.74) or latency (*F*_1.62,14.57_ = 1.33, *p* = 0.29). Consistent results were observed in the dual-task condition (Fig. 2D), with no significant changes in P300 peak amplitude (*F*_1.89,16.99_ = 2.27, *p* = 0.14) or latency (*F*_1.78,16.06_ = 0.19, *p* = 0.80) across three training days.

To investigate whether P300 characteristics differed between single- and dual-BCI tasks, we compared the P300 amplitude and latency per day using paired comparisons. There was a significant difference in P300 amplitude between the single- and dual-BCI tasks on all three days [Fig. 2B; day 1 (single: 7.0±2.7 $\mu {\rm{V}}$, dual: 4.4±1.5 $\mu {\rm{V}}$, *p* < 0.001), day 2 (single: 6.9±2.7 $\mu {\rm{V}}$, dual: 3.8±1.8 $\mu {\rm{V}}$, *p* < 0.001), day 3 (single: 7.2±2.6 $\mu {\rm{V}}$, dual: 4.7±1.8 $\mu {\rm{V}}$, *p* < 0.01)]. In contrast, no significant difference was observed in P300 latency on all three days [Fig. 2E; day 1 (single: 349±62 ms, dual: 364±113 ms, *p* = 0.77), day 2 (single: 330±56 ms, dual: 372±116 ms, *p* = 0.73), day 3 (single: 373±51 ms, dual: 388±47 ms, *p* = 0.77)]. Moreover, by analysing the coefficient of variation (CV)^24^ of P300 amplitude and latency, we found that there was no significant difference in the CV of P300 amplitude on all three days [Fig. 2C; day 1: *p* = 0.87, day 2: *p* = 0.50, day 3: *p* = 0.50]. Comparatively, there was a significant difference in the CV of P300 latency between single- and dual-BCI tasks on day 1 (*p* < 0.01) and day 2 (*p* < 0.001), whereas not on day 3 (*p* = 0.12) (Fig. 2F). Pearson’s correlation analysis revealed a significant negative correlation between peak P300 amplitude and latency CV (Fig. 2G, H; single: *R*^2^ = 0.67, *p* < 0.001, dual: *R*^2^ = 0.52, *p* < 0.001), indicating that lower latency CV reflects higher consistency in P300 responses, thereby enhancing the BCI performance.

Overall, the results indicate that the dual-task condition was associated with reduced P300 amplitude and increased latency variability compared to the single-task condition, indicating greater variability in tactile-evoked responses during concurrent movement. By day 3, latency variability converged toward the level observed in the single-task condition.

### Tactile-evoked P300 allows effective movement augmentation and exhibits learning effects

**Fig. 3:**
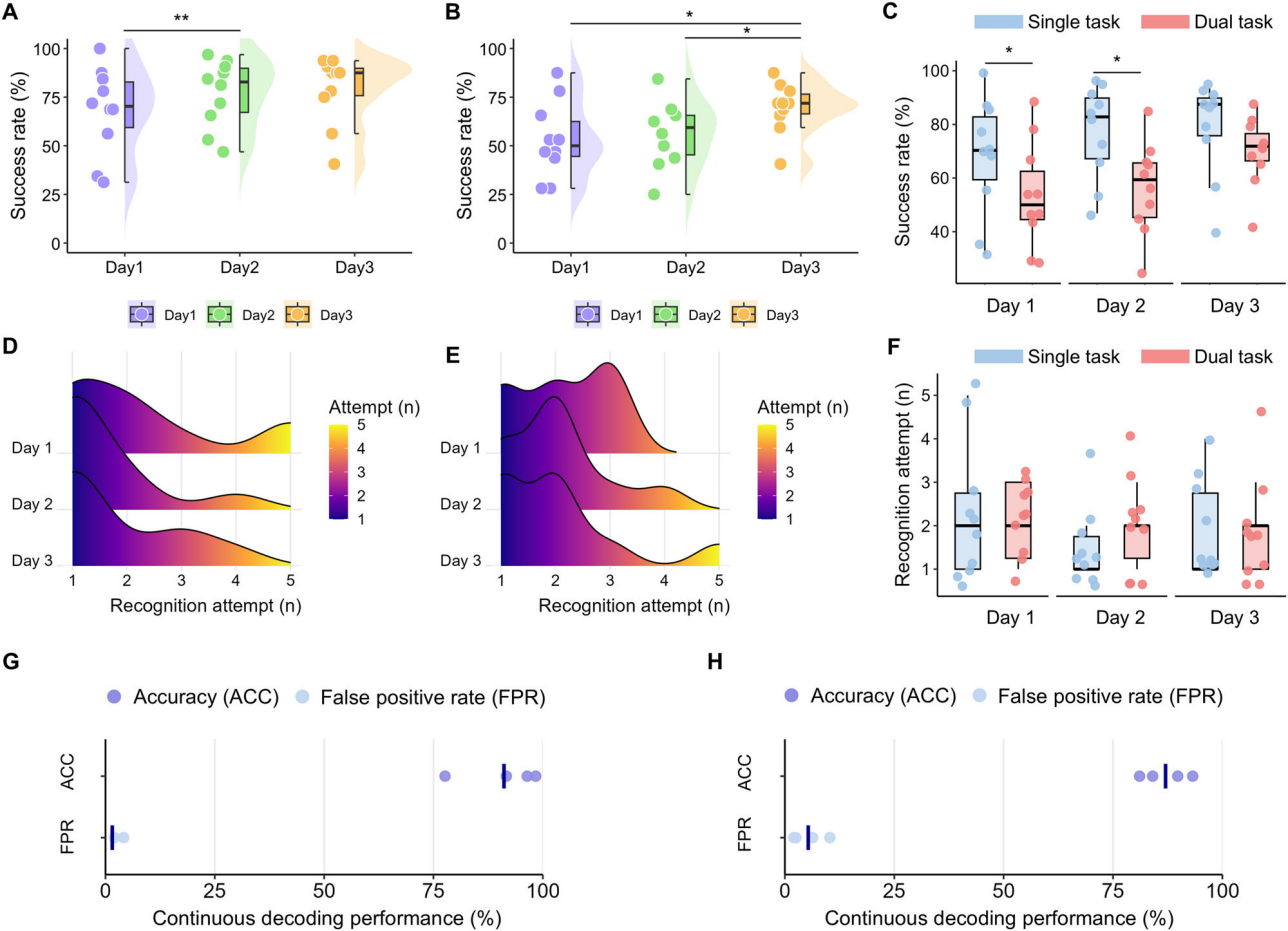
Participant-level online performance of tactile-encoded brain-computer interface over three days of experiment, comparing single-task and dual-task conditions. Success rates for (**A**) single-task and (**B**) dual-task conditions across days 1–3 (*n* = 10 participants). A one-way repeated-measures ANOVA with a Greenhouse-Geisser correction was applied with main effects of training on success rates, followed by post hoc analysis with Bonferroni correction. *P*-values: 0.0080 (**A**, Day 1-Day 2), 0.10 (**A**, Day 1-Day 3), 1.00 (**A**, Day 2-Day 3), 0.95 (**B**, Day 1-Day 2), 0.028 (**B**, Day 1-Day 3), 0.016 (**B**, Day 2-Day 3). Comparisons of (**C**) success rate and (**F**) recognition attempt between single-task and dual-task conditions on each day (*n* = 10 participants). Comparisons were conducted using two-tailed Wilcoxon signed-rank tests or two-tailed paired-sample *t*-tests with False Discovery Rate (FDR) correction for multiple comparisons, depending on whether the differences between paired observations met the assumption of normality. *P*-values: 0.031 (**C**, Day 1), 0.027 (**C**, Day 2), 0.058 (**C**, Day 3), 0.73 (**F**, Day 1), 0.73 (**F**, Day 2), 0.73 (**F**, Day 3). Distribution of the number of recognition attempts before first correct recognition for (**D**) single-task and (**E**) dual-task conditions across days. Continuous decoding performance metrics for (**G**) single-task and (**H**) dual-task conditions, expressed as accuracy (ACC), and false positive rate (FPR) (*n* = 4 participants). Each point represents performance from an individual participant. For all boxplots in the figure, the centre lines indicate median values, box bounds represent the 25th and 75th percentiles, whiskers extend to the minimum and maximum values within 1.5 times the interquartile range. **if *p* < 0.01, *if *p* < 0.05.

The online performance of the tactile-encoded BCI was evaluated with two metrics: (i) the percentage of target stimuli that successfully elicited a detectable P300 response (success rate) and (ii) the number of recognition attempts required to successfully detect such a response. Significant improvements in success rates were observed over training for both the single- (Fig. 3A; *F*_1.40,12.63_ = 4.93, *p* = 0.036) and dual-BCI tasks (Fig. 3B; *F*_1.45,13.04_ = 9.77, *p* = 0.0045). For the dual-BCI task, post hoc analysis with a Bonferroni adjustment revealed that the success rate significantly increased from day 1 to day 3 [−16.56 (95% CI, −31.28 to −1.84) %, *p* = 0.028], as well as from day 2 to day 3 [−13.44 (95% CI, −24.29 to −2.59) %, *p* = 0.016]. However, for the recognition attempts, no significant learning effect was observed for the single- (Fig. 3D; *F*_1.59,14.27_ = 2.74, *p* = 0.11) or dual-BCI tasks (Fig. 3E; *F*_1.57,14.15_ = 0.03, *p* = 0.95).

To further investigate the difference in BCI performance between single- and dual-BCI tasks, we compared the success rate and recognition attempts per day. Significant differences in success rates were observed on day 1 (single: 68.1±22.1%, dual: 53.1±19.4%, *p* = 0.031) and day 2 (single: 77.2±17.2%, dual: 56.3±16.9%, *p* = 0.027), while not on day 3 (single: 79.1±17.6%, dual: 69.7±12.9%, *p* = 0.058) (Fig. 3C). No significant difference was found in recognition attempt on all three days [Fig. 3F; day1 (single: 2.3±1.6, dual: 2.1±0.9, *p* = 0.73), day 2 (single: 1.5±1.9, dual: 2.0±0.9, *p* = 0.73), day 3 (single: 1.8±1.1, dual: 2.0±1.2, *p* = 0.73)]. Moreover, to validate the sustained decoding ability of the BCI, four participants were recruited for an online continuous decoding test (Fig. 3G, H). All participants could maintain the attention to the target stimulus and sustain accurate command recognition (single: 91.1±9.4%, dual: 87.0±5.6%), with a low false positive rate (single: 1.6±2.2%, dual: 5.3±3.9%). The convergence of success rates between the single- and dual-BCI tasks indicates the learning effect with training, and the sustained detection of enduring and continuous control capabilities of the augmented BCI.

### Tactile-encoded BCI does not interfere with natural human movement

**Fig. 4:**
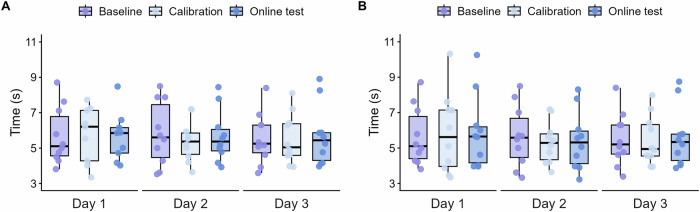
Performance of ball-balancing task. **A** Averaged effective rotation time, and **B** averaged gross rotation time during baseline, calibration, and online testing phases across days 1–3 (*n* = 10 participants). For all boxplots, the centre lines indicate median values, box bounds represent the 25th and 75th percentiles, whiskers extend to the minimum and maximum values within 1.5 times the interquartile range.

To assess the impact of the tactile-encoded BCI on natural movement, participants performed a bimanual ball-balancing task that served as a baseline measure of coordinated motor control. This was selected as a representative continuous motor task that engages sustained movement while minimising confounds from discrete action planning. We evaluated two complementary performance metrics: (i) averaged effective rotation time, calculated as the total task duration divided by the number of successful rotations, and (ii) averaged gross rotation time, calculated as the total task duration divided by the total number of rotation attempts, including unsuccessful ones. Detailed definitions and rationale for these metrics are provided in Supplementary Materials. Participants were instructed to perform the task at a steady and controlled pace rather than maximise speed, thereby minimising speed-accuracy trade-offs. A one-way repeated-measures ANOVA revealed no significant differences across the baseline, dual-task calibration and online conditions for either the effective rotation time [Fig. 4A; day1 (*F*_1.64,14.79_ = 2.08, *p* = 0.16; Bayes factors (BF_10_) = 0.729), day2 (*F*_1.12,10.05_ = 0.52, *p* = 0.51; BF_10_ = 0.295), day3 (*F*_1.76,15.86_ = 0.17, *p* = 0.82; BF_10_ = 0.236)], or the gross rotation time [Fig. 4B; day1 (*F*_1.79,16.08_ = 0.32, *p* = 0.70; BF_10_ = 0.262), day2 (*F*_1.21,10.89_ = 0.35, *p* = 0.61; BF_10_ = 0.266), day3 (*F*_1.81,16.25_ = 0.17, *p* = 0.83; BF_10_ = 0.234)]. The BF_10_ for day1 showed inconclusive evidence for the null hypothesis, which could be attributed to participants’ first exposure to the ball-balancing and tactile-encoded P300 control tasks. However, following training, the results of day 2 and day 3 supported the observation that use of the tactile-encoded BCI did not affect the efficiency or temporal stability of natural bimanual coordination.

### Functional task validation with supernumerary robotic arms

**Fig. 5:**
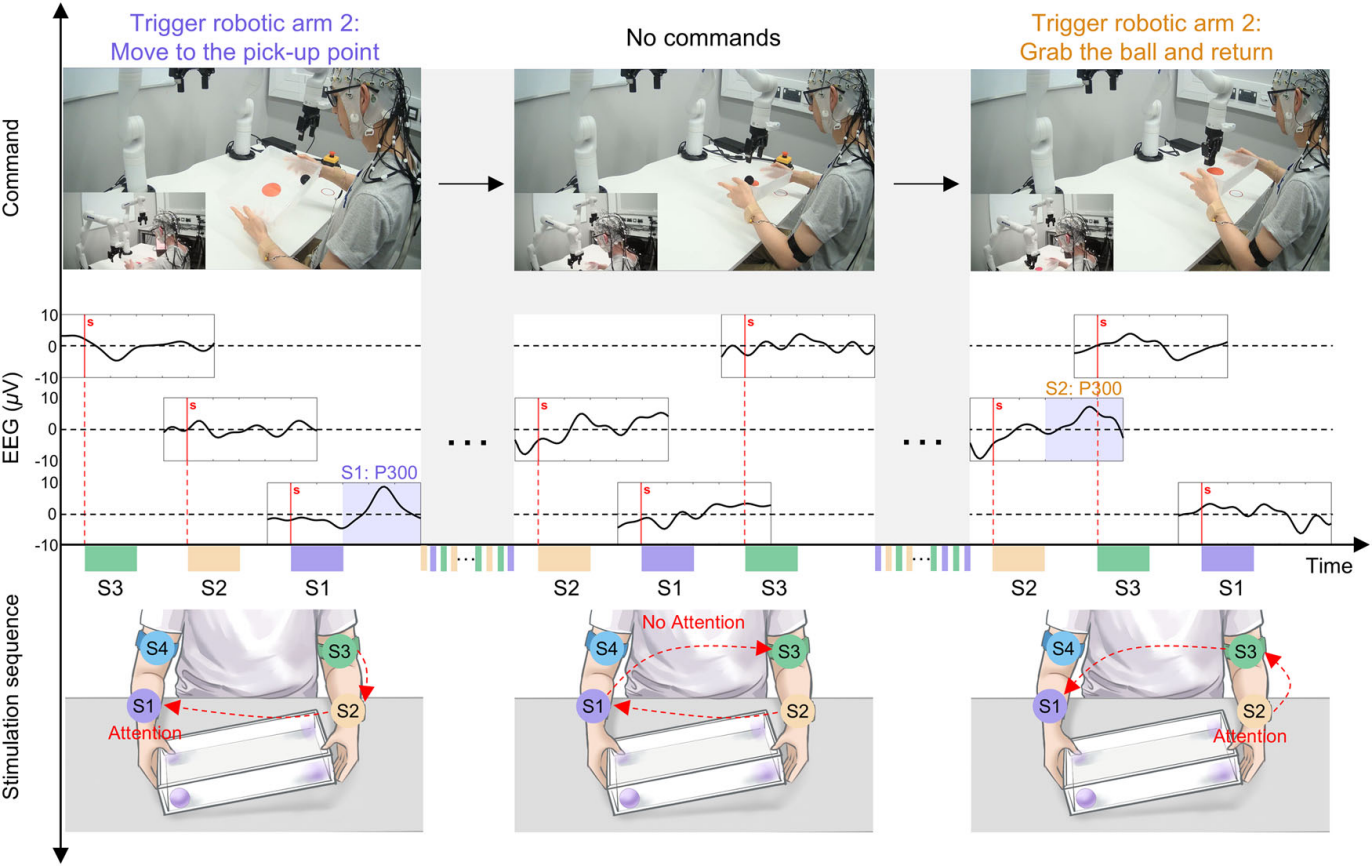
Demonstration of online robotic arm control using tactile-encoded brain-computer interface. Representative trial sequence illustrates how tactile-encoded P300 responses were used to control robotic arms. The bottom panel shows the stimulation sequence from vibrators, with the participant’s attention directed to the designated target vibrator. The middle panel displays corresponding EEG traces, where P300 components (shaded) are elicited by selective attention to the target stimulus (e.g., S1 in the first block, S2 in the third block). The top panel presents the resulting robotic movements: Arm 2 moves to the pick-up point when a P300 is detected for S1, no command is issued when no P300 is identified, and a subsequent P300 for S2 triggers the grasp-and-return action. Together, these sequences illustrate the translation from attended vibrotactile stimuli to robotic control commands in real time.

Four functional tasks were designed to demonstrate the performance of the tactile-encoded BCI for supernumerary robotic arm control (Supplementary Movies S1–S4). These functional tasks needed sequential and continuous recognition of multiple commands to achieve task objectives. In the first task, the participant provided unimanual commands to the robotic arms to complete a ball-grasping task (Fig. 5, Supplementary Movie S1). Starting from their home positions, the robotic arms could be freely selected, either Arm 1 or Arm 2, to perform two possible actions: (i) move to the pickup location and (ii) grasp the ball to return home. This task required both accurate sequential execution and correct arm selection, introducing an additional control challenge. The second task required sequential control of both supernumerary arms to place a straw into a cup while the participant’s natural limbs remained engaged in a separate task (Supplementary Movie S2). The participant first commanded Arm 1 to pick up the cup, then instructed Arm 2 to grasp and drop the straw and finally directed Arm 1 to return the cup to the table. This demonstration highlighted the potential of coordinated bimanual supernumerary control to achieve a shared goal. In the third dual-arm workflow (Supplementary Movie S3), the arms performed a cyclic sequence in which Arm 1 repeatedly picked up a ball and returned home, while Arm 2 transported the ball to a drop-off point, illustrating reliable repetition of multi-step coordination. In the final demonstration, the participant used the BCI to guide a single robotic arm through incremental movements on a two-dimensional workspace (Supplementary Movie S4). Through continuous decoding of P300 responses, this task demonstrates how successive discrete neural interface outputs are integrated over time to enable incremental control of the robotic arm. Across all demonstrations, the participant executed the correct temporal sequence of commands required for successful task completion, even while concurrently engaged in natural bimanual movement.

## Discussion

In this study, we presented a method for supernumerary robotic arm control using a tactile-encoded BCI without compromising natural human movement. Our results first demonstrated the feasibility of evoking P300 responses through vibrotactile stimulation, exploiting the tactile receptors distributed throughout the human body. We then encoded the relationship between tactile perception and user commands. By combining the evoked P300 with prior tactile encoding, the proposed BCI increased the controllable DoFs by the number of tactile vibrators, without requiring separation of overlapping neural activity. In our experiments, we achieved an online task success rate of 69.7% when decoding tasks between four potential targets without compromising the concurrent natural human movement. Additionally, the performance of the BCI significantly improved over three days of training, indicating that practise promoted skill acquisition. This learning effect further promotes the realistic application of our system, as the performance would improve over continuous use.

A central challenge in practical movement augmentation is expanding controllable DoFs without compromising natural movement performance. Previous studies demonstrated that leveraging output-null neural dimensions and kinematic redundancy could provide additional control dimensions for supernumerary limbs. However, achieving stable and intuitive decoupling of augmented and natural motor intentions remains challenging. For instance, foundational studies on superimposing BCI commands over natural movement highlighted this difficulty; while Penaloza and Nishio demonstrated BMI control of a third arm for multitasking^2^ and Bashford et al. achieved concurrent invasive BCI control with overt movements^6^, this simultaneous control was typically limited to a single degree of freedom. Recent human movement augmentation studies explored control dimensions decoupled from the primary motor task. Dominijanni et al. presented an augmentation-by-transfer approach in which gaze and respiration were used to control a robotic arm without interfering with natural limb movements, demonstrating decoupled control with a limited control bandwidth^25^. Umezawa et al. demonstrated that independent supernumerary limbs could be embodied at the perceptual level, while the controllability remained modest^26^; Baldi et al. systematically reviewed the kinematic redundancy schemes to control supernumerary robotic limbs and concluded that while mapping approaches are feasible, they are often task-specific and difficult to generalise^27^; A consistent conclusion was achieved by Lee et al. from the exploration on null-space muscle activation patterns^8^. These studies demonstrated that while null-space and kinematic redundancy strategies can, in theory, provide additional control dimensions, their practical use is often constrained by coordination difficulties, the need for substantial user training, and limited scalability. Instead of separating motor intentions from superimposed motor-related neural activity in a top-down manner, we built a bottom-up approach that encoded neural markers by leveraging sensory afferents through changing attention to vibrotactile stimuli. Our tactile-encoded BCI maps tactile attention to augmented motor output, utilising the tactile perception of human body to provide supernumerary control dimensions.

Our tactile-encoded BCI enables movement augmentation, expanding the control dimensionality by four more DoFs. Although recent non-invasive BCIs have extended online decoding beyond binary control, their performance typically decreases as the number of classes increases. For instance, a tactile P300 system achieved an accuracy of 73% with two vibrotactile targets but dropped to 58% with six targets in a single BCI task^28^. Likewise, a finger-level EEG decoder using deep learning reached an accuracy of 80.56% for a two-finger motor imagery task but decreased to 60.61% for a three-finger task^29^. Moreover, only a few multi-class BCI studies have considered the dual-task conditions. SSVEP-based BCIs have demonstrated a successful application in a four-class control task during concurrent treadmill walking, with an information transfer rate above 12 bit/min. However, the system relied on gaze-dependent visual stimuli^30^. Similarly, tactile ERP BCIs have been tested under a multimodal dual-task paradigm, with significant performance degradation while introducing the secondary task^31^. In comparison, for a four-class online decoding task, our system achieved an average accuracy of 74.79% in the single-BCI task and an average accuracy of 59.69% in a dual-BCI task across participants. The expansion of DoFs is attributable to the tactile-encoded P300 paradigm, which does not require distinguishing P300 patterns across vibrators. Extending to four DoFs still remains binary, not four-class, classification. The classifier simply detects the presence of a P300, and the system identifies the intended command by the corresponding vibration onset time. This reduces decoding complexity while increasing control dimensionality. These online-test results demonstrate that robust four-target online control is feasible even under simultaneous motor demands in a non-visual, tactile paradigm.

The enhancement in BCI performance following training is closely linked to the evolution of the P300 component. Across three days of training, the P300 peak amplitude under the single-BCI task was consistently higher than that in dual-BCI task. However, no significant difference in P300 amplitude and latency was found across all three days. As we cannot voluntarily modulate the P300 with training, how did the performance of the dual-BCI task improve with training, and what are the underlying mechanisms that drove this improvement? The decoding performance of the BCI relies on distinguished features, such as the higher P300 peak amplitude and their greater similarity across trials. To quantify this similarity, we introduced CV as a metric to evaluate the trial-to-trial variability^32,33^. Lower CV values indicate a leptokurtic distribution, reflecting greater consistency in P300 responses across trials, which may enhance decoding performance. As shown in Fig. 2F, by three days of training, no significant difference was observed in the CV of latency between single- and dual-BCI tasks. This indicates that the trial-to-trial variability of the P300 latency tends to converge across both tasks, enabling the model to more reliably capture the key characteristics of the P300 peak at specific time points. Additionally, a significant negative correlation between P300 peak amplitude and latency CV (Fig. 2G, H) further indicated that lower latency variability results in a higher P300 amplitude after trial averaging, which ultimately enhances the performance of the model. We thus hypothesise that through training, participants learned to effectively allocate attention during the dual BCI tasks, leading to more stabilised P300 latency. However, it was not possible to infer a learning effect in BCI control solely from changes in the CV of the P300 latency, especially because other metrics of performance, such as information transfer rate, did not fully correlate with CV. While performance improvements were observed across sessions in the dual-task condition, no significant learning effect was detected in the single-task condition. This pattern may indicate that performance in the single-task condition approached the effective ceiling for this paradigm, whereas the additional attentional and coordination demands in the dual-task condition likely left greater room for improvement. The continuous performance improvement observed on day 2 and day 3 suggests that dual-task performance may not have reached its ceiling. Future studies with longer training time could help determine whether further improvements can be achieved under dual-task conditions.

Although convergence in P300 latency variability following training offers a plausible explanation for the observed performance gains, the mechanisms underlying dual-task effects are likely more complex. Voluntary movement is known to modulate somatosensory processing via sensory gating, whereby afferent signals are partially attenuated during active movement^34^. Thus, the dual-task effects observed here likely arise from an interaction among top-down attentional allocation, increasing task familiarity, and movement-related sensory gating. Within this framework, we deliberately selected the ball-balancing task as a canonical continuous manipulation paradigm that engages ongoing motor coordination while minimising confounds related to fine dexterity, grip-force modulation, or discrete movement planning. This experimental design enabled us to probe the robustness of bottom-up, tactile-evoked P300 responses during continuous movement, without introducing additional sources of motor complexity that could obscure interpretation of training-related changes in BCI performance. Furthermore, the enhancement of the sense of embodiment may also contribute to performance gains. In future studies, this could be investigated by questionnaires to assess whether users could develop a sense of embodiment and improve it with training.

For human-machine interaction systems’ real-world applications, the speed with which neural interfaces decode control intentions and generate output commands is of central importance. The temporal characteristics of the P300, combined with the need for repeated stimulation to elicit a distinct response, introduce decoding delays that can limit responsiveness in quick-response applications^35^. In our study, after three days of training, most targets were recognised within the first or second recognition cycle, indicating that reliable supernumerary command selection could be achieved with relatively few cycles. Further improvements in responsiveness will depend on the advances in single-trial P300 decoding, although such approaches must balance decoding accuracy, computational cost, and real-time feasibility. De Venuto et al. developed an embedded single-trial P300 decoder that increased the BCI responsiveness, while the validation was limited to only speller data^36^. Leoni et al. introduced an explainable deep learning pipeline that improved single-trial decoding accuracy with increased computational cost^37^. Du et al. reported that fusing multi-subject CNN features boosted the accuracy of single-trial P300 decoding to above 80%, despite requiring large datasets and training overhead^38^. Kim et al. demonstrated a calibration-free “plug-and-play” single-trial BCI using pre-trained spatial filters, showing promising real-time use^39^. In parallel, Eidel et al. observed that tactile P300 could be recorded using ear-mounted electrodes, highlighting the practical trade-off between improved wearability and reduced signal quality^40^. Collectively, these developments suggest that improving single-trial decoding while maintaining high computational efficiency will be important for enhancing the practical performance of our proposed tactile-encoded BCIs. In addition to decoding speed and computational efficiency, tactile adaptation to repeated vibrotactile stimulation may also affect real-world applications. Repeated exposure may reduce the salience of the stimulus and thereby lessen the attention allocated to it, which could contribute to a weaker P300 response over time. Although this effect was not systematically evaluated in the present study, it may influence BCI performance during extended real-world operation. Future work should investigate how prolonged tactile stimulation influences BCI performance and user experience during extended use.

Neural interfaces are increasingly transitioning from discrete, switch-based control towards continuous control paradigms. For real-time BCI applications, control that is continuously proportional to a neural signal is desirable compared to a simple action trigger, as it may allow direct control over the robotic device’s range of motion^41^. Such control method relies on the ability of users to voluntarily modulate neural rhythms through real-time neural feedback. For example, for endogenous paradigms such as MI, the modulation of SMRs can be utilised to drive continuous control. However, whether users can learn to independently modulate sensorimotor rhythms for concurrent supernumerary control and identical natural limb movement using non-invasive EEG remains an open question, representing an important direction for future investigation. In contrast, a P300 is an event-related potential elicited by infrequent stimuli. Because it relies on externally presented stimuli, it is ill-suited for proportional regulation of robotic effectors^20,42^ and is generally limited to providing discrete control commands^18^. Although P300 characteristics in this study exhibited significant differences between single- and dual-BCI tasks due to the variation in attention to the target stimuli^43^, its characteristics cannot be continuously modulated through training. Accordingly, it is difficult to design neural feedback that would allow subjects to voluntarily modulate P300 characteristics for proportional control. To address this limitation and aim at continuous P300 control, we proposed to replace the modulation of P300 characteristics with a continuous detection of P300. This approach translates the sustained, uninterrupted detection of P300s into a continuous control signal (e.g., Supplementary Movie S4: Moving a robotic arm between two positions) rather than event-based commands, allowing users to navigate the supernumerary workspace. By testing the continuous P300 recognition on a subset of four participants, we showed that the continuous detection had high control accuracy and a low false positive rate. This demonstrated that sustained P300 recognition could mitigate the inherent discreteness of the P300 paradigm. Our further work will combine the continuous P300 detection with a ReFit-Kalman filter, enabling continuous and smooth neural control^44^.

## Methods

### Participants

Ten able-bodied participants (six males, four females, aged between 23 and 37 years) volunteered for this study. All participants were right-handed, possessed normal or corrected-to-normal vision, and had no history of neurological disorders. Two participants had BCI experience, while the others were naïve to BCI. This study was approved by the Imperial College Research Ethics Committees (ICREC reference 18IC4685). Prior to the experiment, participants received an explanation of the experiment procedure and provided signed informed consent. Potential video recording during the experiment was also approved by all participants. Consent to publish subject-identifiable information was obtained. The authors affirm that human research participants provided informed consent for publication of the images in Fig. 5, Supplementary Fig. S1, as well as Supplementary Movies S1–S4.

### EEG system and vibrotactile stimulator

EEG data were recorded at 1000 Hz using a 64-channel EEG cap and an actiCHamp Plus amplifier (Brain Products GmbH, Gilching, Germany) with a customised GUI (Supplementary Fig. S2) using MATLAB (2024a, The MathWorks Inc., Natick, MA, USA). EEG electrodes were positioned according to the international 10-10 system. A ground electrode was placed on the forehead. Electrode impedances were maintained below 5 kΩ. Event markers indicating stimulus onset and vibrator identity were transmitted via a parallel port to ensure precise alignment.

Four C2-HDLF vibrators (Engineering Acoustics Inc., Casselberry, FL, USA) were attached bilaterally to the participants’ upper and lower arms using adjustable straps (Supplementary Fig. S3). Each stimulus consisted of 200-ms bursts of vibration with 200-ms interstimulus intervals. Each vibrator location was associated with a predefined command throughout the experiment. This mapping was kept consistent across participants, while the presentation order of the vibrotactile stimuli within each run was pseudorandomized according to the oddball paradigm. The vibrators were positioned symmetrically to avoid asymmetric proprioceptive cues.

### Experimental design

#### Baseline ball-balancing phase

At the beginning of each experimental session, participants were instructed to perform a ball-balancing task to familiarise themselves with the baseline task. Using a lightweight rectangular plastic tray (31.5 × 23.5 cm), they were instructed to roll a lightweight ball sequentially around all four corners at a steady pace. After practice, participants were required to perform a 60-second ball-balancing task for baseline task performance assessment. An overhead camera recorded the movement of the ball throughout the session. A rotation was considered successful only if the ball passed through all four corners; incomplete rotations were marked as unsuccessful.

#### Exploratory and calibration phase

Participants were first introduced to the tactile-encoded BCI and the dual-task requirements. To familiarise themselves with the protocol, they completed one single-task and one dual-task exploratory run designed to confirm that all vibration sites were perceived consistently. In each run, one vibrator was designated as the target, and participants silently counted its activations while ignoring non-targets. During dual-task runs, the same procedure was performed while simultaneously performing the ball task. This exploratory phase ensured that participants understood the task demands before the BCI calibration phase. Calibration was conducted separately for the single-task and dual-task conditions. Four vibrators delivered stimuli in a pseudorandom order, with one designated as the attended target. Each run comprised 80 stimuli (20 targets, 60 non-targets), preserving the 1:3 target-to-non-target ratio. Three runs were completed for each of the four target locations. The calibration phase took ~15 min, and the resulting data were used to train participant-specific decoders for online testing.

#### Online evaluation

Participants completed three experimental sessions on separate days. Each session included both single-task (BCI task only) and dual-task (BCI task combined with bimanual ball-balancing) conditions, with the order counterbalanced across subjects. In single-task runs, participants focused solely on the vibrotactile stimuli, whereas in dual-task runs, they performed the ball-balancing and BCI task simultaneously. For each condition, 32 online tests were conducted, with each of the four vibrators serving as the target eight times in a randomised order. At the beginning of each run, a cue displayed on the screen in front of the participant indicated the target to focus on (Supplementary Fig. S2). Participants began the ball-balancing task at their own pace following run onset. Feedback was presented on the screen, where the marker corresponding to the current decoding result was illuminated in blue.

Given that the P300 response typically requires trial averaging to achieve a detectable peak, a sliding-window approach was adopted. EEG responses evoked by four consecutive stimuli with the same vibrator were averaged and fed into the decoder. Each stimulation round consisted of four distinct vibration stimuli (one stimulus per vibrator), delivered in a pseudorandom sequence, with a total duration of 1.6 s per round. Due to the requirement of trial averaging, the first recognition attempt occurred after the completion of the 18th trial. Thereafter, a decoding decision was made after each set of four consecutive trials, resulting in a total of 16 attempts per run. Each of the four vibrators corresponded to one BCI command. The classifier output for each vibrator fell into one of two categories: (i) a P300 was detected (triggering the command) or (ii) no P300 was detected (no command output). Online testing continued until one of three outcomes occurred: (i) the target was correctly classified (success), (ii) a non-target was identified (failure: false non-target recognition), or (iii) all 80 trials were finished (failure: missed recognition). After each run, participants were given a 7 s break before the next run. The number of recognition attempts required until the target P300 was detected was recorded to represent the response time, and the number of successful online tests out of 32 tests was recorded as the success rate, serving as a metric for evaluating the responsiveness of BCI control. This online evaluation phase modelled the simultaneous control of supernumerary effectors during ongoing motor activity. The experimental protocol was repeated on three separate days to assess potential learning effects on decoding accuracy and P300 waveform modulation. To explore the feasibility of continuous decoding, four out of ten participants completed an additional test in which sustained P300 detections across sliding windows were treated as continuous control inputs. Across 80 trials, participants were instructed to maintain attention on a single target and sustain recognition for as long as possible. The indicator light remained illuminated when consecutive trials were correctly decoded.

### Signal processing and BCI decoder training

#### EEG pre-processing

EEG from each run was pre-processed using a standard sequence. Data were epoched from −0.1 to 0.7 s relative to stimulus onset. A finite impulse response band-pass filter (1–10 Hz) and a notch filter (48–52 Hz) were applied to remove slow drifts and high-frequency noise. The signals were then re-referenced to the average of electrodes M1 and M2, placed on the mastoid processes, to reduce reference-dependent artefacts. After baseline removal, data were downsampled to 100 Hz. For each trial, the post-stimulus interval of 100–500 ms was extracted, as the P300 peaks typically appear around 300 ms^20^. This window emphasises the positive deflection while excluding later potentials. Due to substantial contamination from eye blinks and facial muscle artefacts, seven EEG channels over the frontal regions (Fp1, Fp2, AF7, AF3, AFz, AF4, and AF8) and two temporal electrodes (FT9, FT10) were excluded from the P300 analysis. M1, M2, and FCz, which are commonly used as references in EEG analysis, were also excluded from analysis. The remaining electrodes were all included for offline analysis, whereas those exhibiting observable P300 responses were used for online decoding.

#### BCI decoder calibration

At the single-trial level, the P300 ERP exhibits a low signal-to-noise ratio. To improve detectability, sliding-window averaging was applied where EEG responses from four consecutive stimuli at the same vibrator were averaged, thereby reducing random fluctuations. Feature extraction was then performed using the xDAWN algorithm^45^, which learns spatial filters that maximise the variance of target ERPs relative to overall variance, thereby enhancing the P300 response while suppressing background activity. The resulting spatio-temporal features were standardised and classified using a support vector machine, a method shown to perform reliably in P300 BCIs^46^. Classification was carried out on trial-averaged data to balance noise reduction and response latency.

#### P300 analysis

We analysed the P300 event-related potential. For each run, target and non-target trials were averaged respectively within participants and then pooled to generate grand averages across the group. Channel Cz was chosen for analysis because it showed the largest P300 amplitude across all ten participants during the first calibration phase on Day 1, serving as a baseline. Prior studies have reported that tactile P300 amplitudes increase with training^47^. In this work, we tested whether comparable neurophysiological changes would emerge when the paradigm is applied to movement augmentation.

### Statistics

Statistical analysis was performed using IBM SPSS Statistics and R. G*Power^48^ was used for a post hoc power analysis. For the online BCI performance evaluation, success rate and recognition attempt were analysed across different days and between single- and dual-task conditions. For the P300 characteristics, P300 peak amplitude and latency were analysed both across different days and between the single- and dual-task conditions. Furthermore, their CVs were compared between the two conditions. Natural movement performance was compared among the baseline, dual-task calibration test, and dual-task online test. When no differences were found among conditions, the evidence supporting the null hypothesis was quantified using Bayes factors. A one-way repeated-measures ANOVA with a Greenhouse-Geisser correction was applied with main effects of training on success rate, recognition attempt, P300 peak and latency, and natural movement performance, followed by post hoc analysis with Bonferroni correction. Two-tailed Wilcoxon signed-rank tests or two-tailed paired-sample *t*-tests were applied to compare BCI performance and P300 characteristics between single- and dual-task conditions with False Discovery Rate (FDR) correction for multiple comparisons, depending on whether the differences between paired observations met the assumption of normality. Pearson’s correlation analysis was performed to examine the relationship of P300 characteristics, including P300 peak, latency and their CV, with two-tailed tests of significance. The statistical significance level was set to 0.05.

### Ethics

Every experiment involving animals, human participants, or clinical samples have been carried out following a protocol approved by the Imperial College Research Ethics Committees. Each participant gave informed written consent.

### Reporting summary

Further information on research design is available in the Nature Portfolio Reporting Summary linked to this article.

## Supplementary information

Supplementary Information

Description of Additional Supplementary Files

Supplementary Movie 1

Supplementary Movie 2

Supplementary Movie 3

Supplementary Movie 4

Reporting Summary

Transparent Peer Review file

## Source data

Source Data

## Data Availability

All data supporting the findings of this study are available within the article and its supplementary files. Any additional requests for information can be directed to, and will be fulfilled by, the corresponding authors. The data for this study are publicly available at 10.5281/zenodo.17306307. Source data are provided with this paper.

## Code availability

The code for online EEG pre-processing, decoding as well as P300 signal plotting used in this study is available on GitHub at: https://github.com/jty940529/Tactile_P300_BCI.
