# Supplementary material for: Concurrent control of natural and robotic limbs through a tactile-encoded brain-computer interface
Source: Nat Commun. 2026 Jul 2;17:8229. doi: 10.1038/s41467-026-75213-3 (PMC13462973; doi:10.1038/s41467-026-75213-3)
Supplement: Supplementary file 1 [file 41467_2026_75213_MOESM1_ESM.pdf]

Supplementary Materials for

Concurrent control of natural and robotic limbs through a tactile-encoded brain-computer interface

Tianyu Jia, Xingchen Yang, Ciaran McGeady, Yifeng Li, Jinzhi Lin, Kit San Ho, Feiyu Pan, Linhong Ji, Chong Li, Dario Farina

This PDF file includes:

Supplementary Methods and Results

Supplementary Figures S1 to S15

Supplementary Tables S1 to S2

Captions for Supplementary Movies S1 to S4

Other Supplementary Materials for this manuscript:

Supplementary Movies S1 to S4

Supplementary Methods and Results

Statistical power analysis

To evaluate whether the sample size (n = 10) was adequate to support the main findings, we performed a post$f = 1.043$hoc power analysis using G*Power. The analysis was conducted on the primary outcome measure: the online success rate for dual-task runs across days 1–3. The analysis revealed a large effect size (Cohen's $\eta_p^2 = 0.521$, $\eta_p^2 = 0.521$), resulting in a statistical power exceeding 0.99 with the sample of 10 participants. This result demonstrates that the sample size is sufficiently large to detect the significant effects reported in this study.

Tactile vibrator setup

Four C2-HDLF vibrators (Engineering Acoustics Inc., Casselberry, FL, USA) were secured bilaterally to the participants' arms using hypoallergenic medical tape (supplementary Fig. S3). The vibrators were placed on upper limbs to correspond with the two robotic arms, rather than on the torso or lower limbs. The hands were excluded as potential placement sites to avoid interfering with natural finger movements. The vibrators were positioned symmetrically near the elbow and wrist joints on both sides to minimise proprioceptive bias and ensure balanced perception across both arms. Each vibrator operated at a fixed frequency of 60 Hz and delivered brief vibration bursts of 200-ms duration with 200-ms interstimulus intervals. Stimuli were presented in a pseudorandom order across the four sites to avoid anticipatory effects and ensure reliable elicitation of event-related potentials. At the beginning of each experimental session, the skin was cleaned with alcohol wipes, and the intended stimulation sites were marked with a skin-safe marker to support consistent placement across days. This procedure minimised day-to-day variability in tactile perception and ensured reproducibility of the evoked responses.

Online BCI testing and its performance evaluation

During the online decoding, feature extraction was performed using the xDAWN algorithm and then classified using a support vector machine (SVM). The classifier's output includes both the category and a confidence score. A confidence score greater than zero indicates the detection of a P300, while a score less than zero indicates no P300 detected. In cases where more than one outcome shows the detection of a P300, the outcome with the highest confidence score is selected as the recognised target.

In the online BCI test, only the four-target classification paradigm was implemented, with each vibrator corresponding to a distinct command. To provide a broader assessment of classifier performance, one-, two-, and three-target classification outcomes were retrospectively derived from the four-target online decoding output. This offline-analysis approach enabled performance to be interpreted across progressively reduced decision spaces, illustrating how online performance scales as the number of possible targets decreases. This analysis did not involve training separate classifiers but was performed solely as a post hoc robustness evaluation, with the four-target condition representing the actual online control setting used in online BCI experiments. Success rates (supplementary Fig. S6), false non-target recognition rates (supplementary Fig. S7), and missed-recognition rates (supplementary Fig. S8) were expressed for the one-, two-, three-, and four-target conditions.

Quantifying ball-balancing performance

Natural movement performance during the ball-balancing task was quantified using two complementary temporal metrics. The first metric, averaged effective rotation time, was calculated as the total task duration divided by the number of successfully completed rotations. A rotation was considered successful when the ball passed sequentially through all four corners of the tray. This metric reflects execution efficiency conditional on correct task completion. The second metric, averaged gross rotation time, was calculated as the total task duration divided by the total number of rotation attempts, including unsuccessful rotations. Because unsuccessful rotations reduced the effective completion rate, this measure captures the overall temporal stability of task performance and implicitly incorporates the impact of errors. Participants were instructed to perform the task at a steady and controlled pace rather than as quickly as possible. Under these conditions, the two metrics provide a complementary measure of coordination efficiency and stability during concurrent BCI use.

Safety constraints for robotic motion

For safety purposes, the robotic arms operated under predefined motion constraints during the functional demonstration tasks. These constraints included limits on joint velocity, acceleration, and workspace boundaries enforced by the robot's built-in control framework. In addition, commands issued by the BCI triggered only predefined motion primitives rather than continuous free-space control, ensuring that all executed movements followed validated trajectories. An emergency button was provided for both participants and experimenters to ensure safety. These safety constraints were applied uniformly across all experimental runs and did not alter the decoding process or the evaluation of BCI performance metrics.

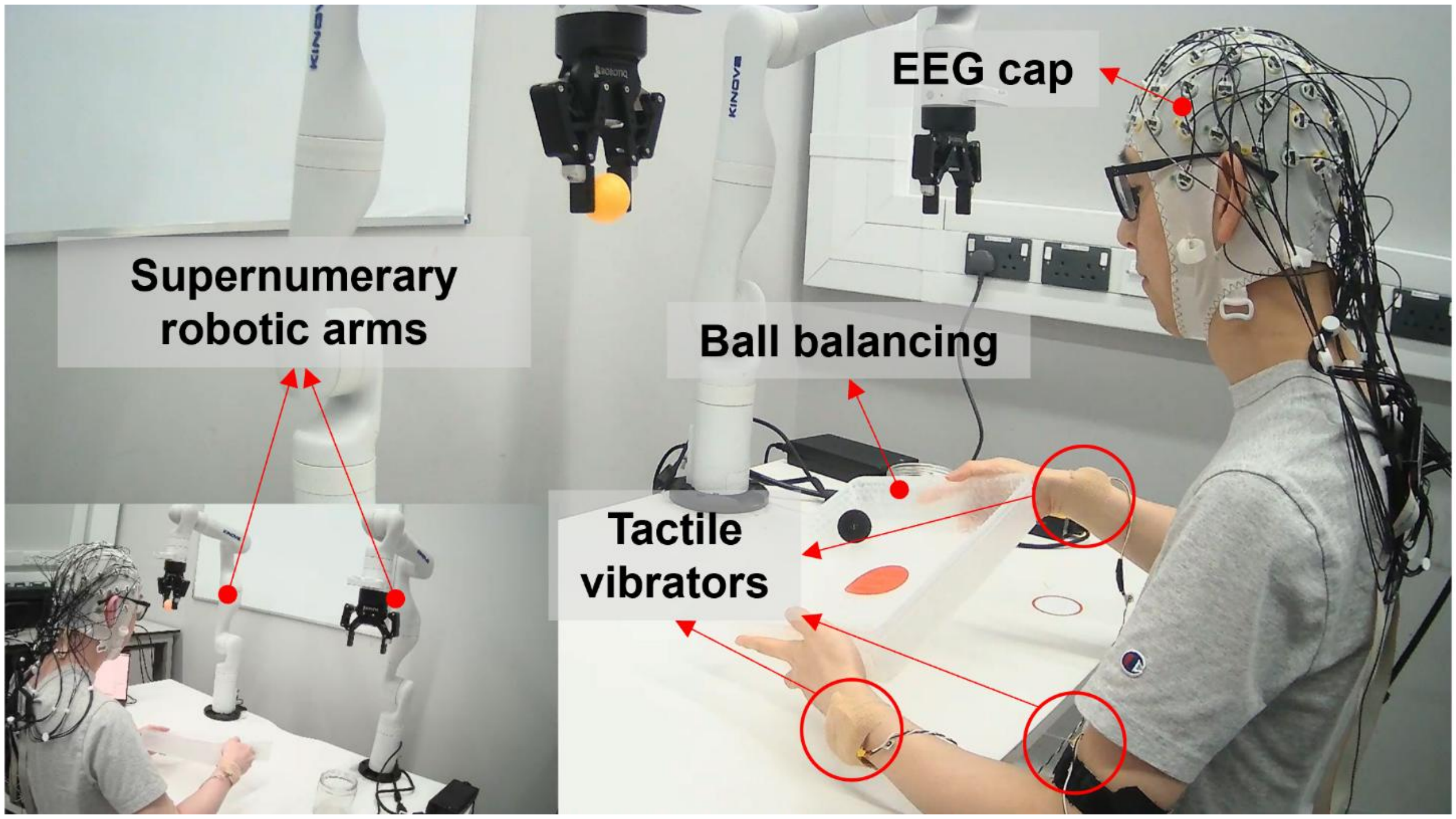

Fig. S1. Experiment setup for the control of supernumerary robotic arms. Participants wore an EEG cap (actiCAP, Brain Products GmbH, Gilching, Germany) for EEG acquisition while performing a concurrent ball-balancing task. Vibrators were attached to the upper and lower arms to deliver tactile stimuli, each corresponding to a specific robotic command. Supernumerary robotic arms (Kinova Gen3, Kinova Inc., Boisbriand QC, Canada) executed the decoded commands triggered by the tactile-encoded brain-computer interface in real time, enabling coordinated and augmented movement control.

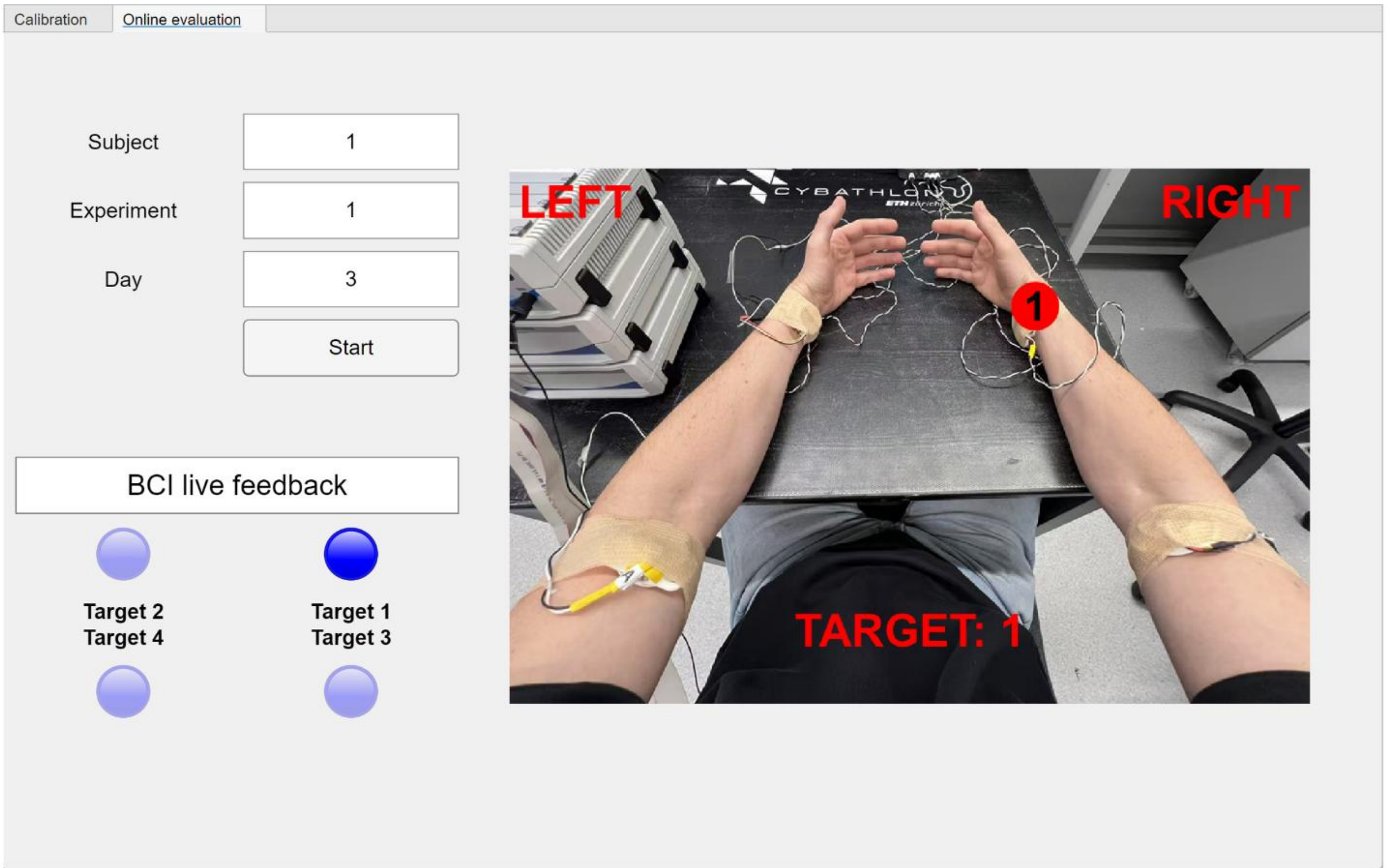

Fig. S2. Customised graphical user interface. Graphical user interface was implemented in MATLAB (2024a, The MathWorks Inc., Natick, MA, USA). The left panel displays subject ID, experiment number, and session day, along with a control button to start the run. The lower left panel provides online BCI feedback by illuminating the marker corresponding to the real-time decoding result. The right panel shows an on-screen cue (TARGET: 1) indicating which vibrator the participant should attend to during the run.

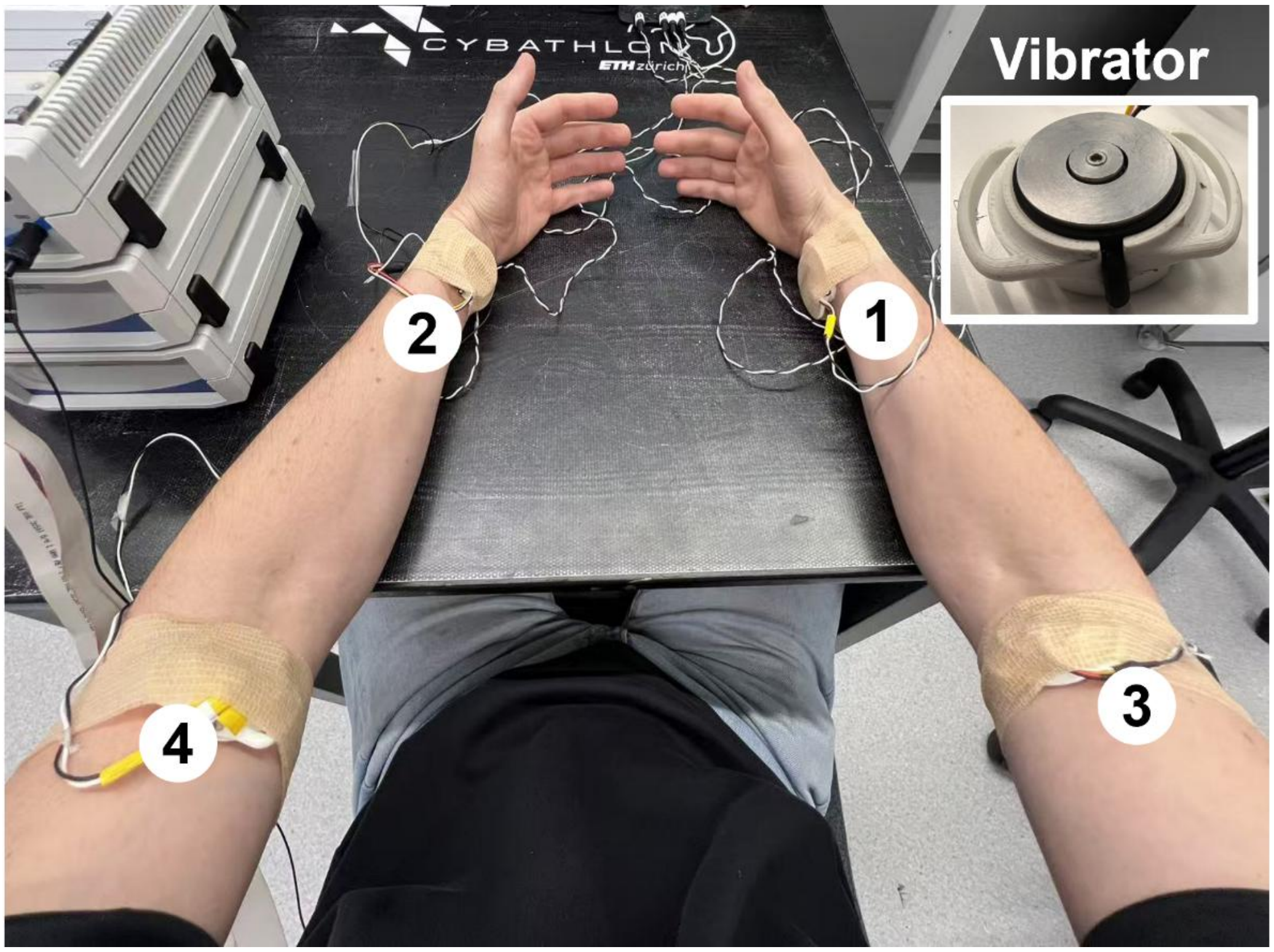

Fig. S3. Experiment setup for vibrotactile stimulation. Four C2-HDLF vibrators (Engineering Acoustics Inc., Casselberry, FL, USA) were attached bilaterally to the arms with medical tape. Each operated at 60 Hz and delivered 200-ms bursts with 200-ms interstimulus intervals in a pseudorandom order during the BCI task. The inset shows a close-up of the vibrator.

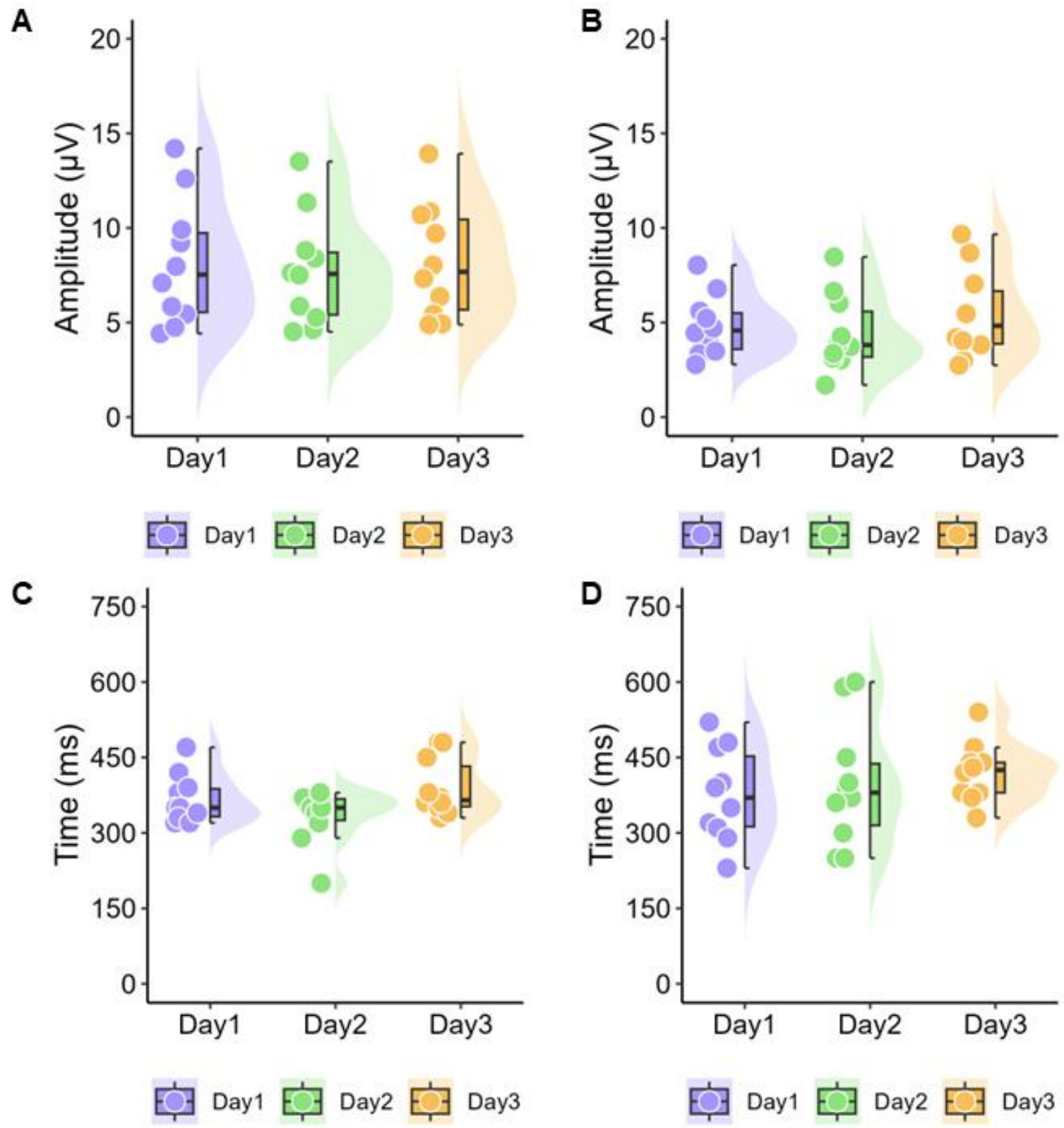

Fig. S4. P300 amplitude and latency over three days of training. P300 peak amplitude at Cz across days 1-3 under **(A)** single-task and **(B)** dual-task conditions (n = 10 participants). P300 peak latency across days 1-3 under **(C)** single-task and **(D)** dual-task conditions (n = 10 participants). Violin plots show participant-level distributions with embedded boxplots. The centre lines indicate the median values, box bounds represent the 25th and 75th percentiles, whiskers extend to the minimum and maximum values within 1.5 times the interquartile range. A one-way repeated-measures ANOVA with a Greenhouse-Geisser correction was applied with main effects of training on P300 amplitude and latency, followed by post hoc analysis with Bonferroni correction. No significant difference was found across days for either amplitude or latency.

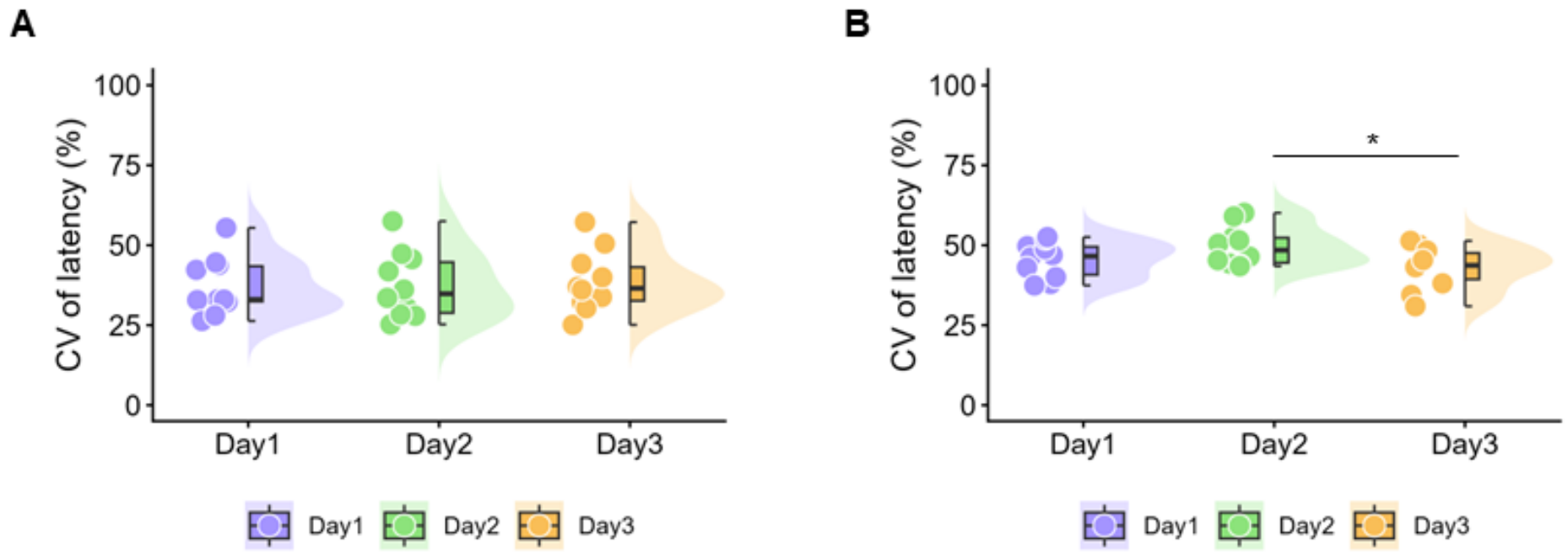

Fig. S5. Coefficient of variation (CV) of P300 latency over three days of training. (A) single-task and **(B)** dual-task conditions (n = 10 participants). Violin plots show participant-level distributions with embedded boxplots. The centre lines indicate the median values, box bounds represent the 25th and 75th percentiles, whiskers extend to the minimum and maximum values within 1.5 times the interquartile range. A one-way repeated-measures ANOVA with a Greenhouse-Geisser correction was applied with main effects of training on CV of P300 latency, followed by post hoc analysis with Bonferroni correction. *P*-value: 0.03 (**B**, Day 2-Day 3). * if $p < 0.05$.

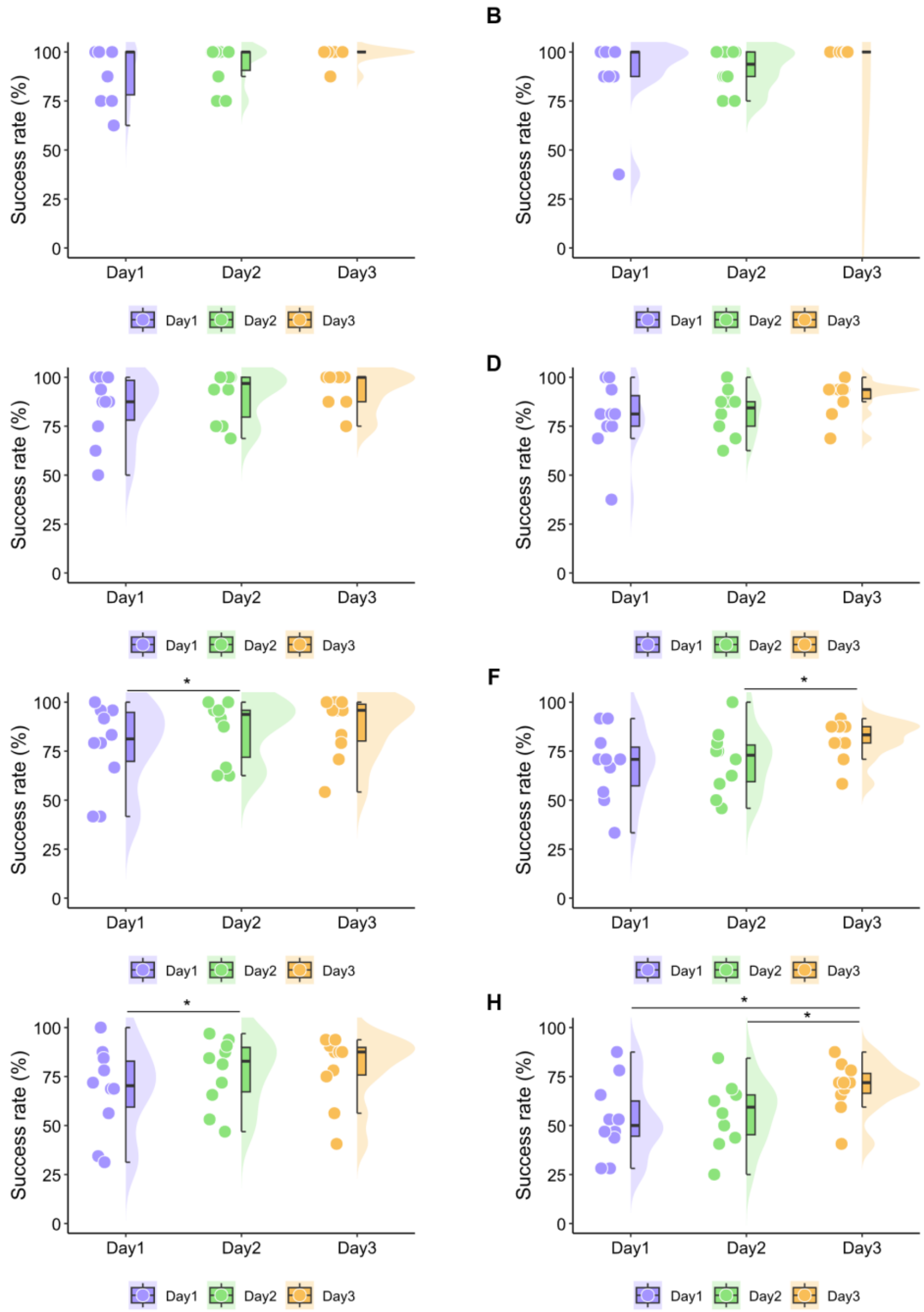

Fig. S6. Success rate distributions of the tactile-encoded brain-computer interface (BCI) across increasing task complexities over three days of training. Violin plots show participant-level success rates with embedded boxplots. Rows correspond to increasing number of classifiable targets: **(A, B)** one target, **(C, D)** two targets, **(E, F)** three targets, and **(G, H)** four targets (n = 10 participants). The first column shows single-task (BCI-only) results, and the second column shows dual-task (BCI with ball-balancing) results. Increasing the number of targets reduced performance initially but improvements were seen across days, with significant gains observed in several conditions. For all boxplots in the figure, the centre lines indicate the median values, box bounds represent the 25th and 75th percentiles, whiskers extend to the minimum and maximum values within 1.5 times the interquartile range. A one-way repeated-measures ANOVA with a Greenhouse-Geisser correction was applied with main effects of training on success rates, followed by post hoc analysis with Bonferroni correction. *P*-values: 0.035 (**E**, Day 1-Day 2), 0.049 (**F**, Day 2-Day 3), 0.0080 (**G**, Day 1-Day 2), 0.028 (**H**, Day 1-Day 3), 0.016 (**H**, Day 2-Day 3). * if $p < 0.05$.

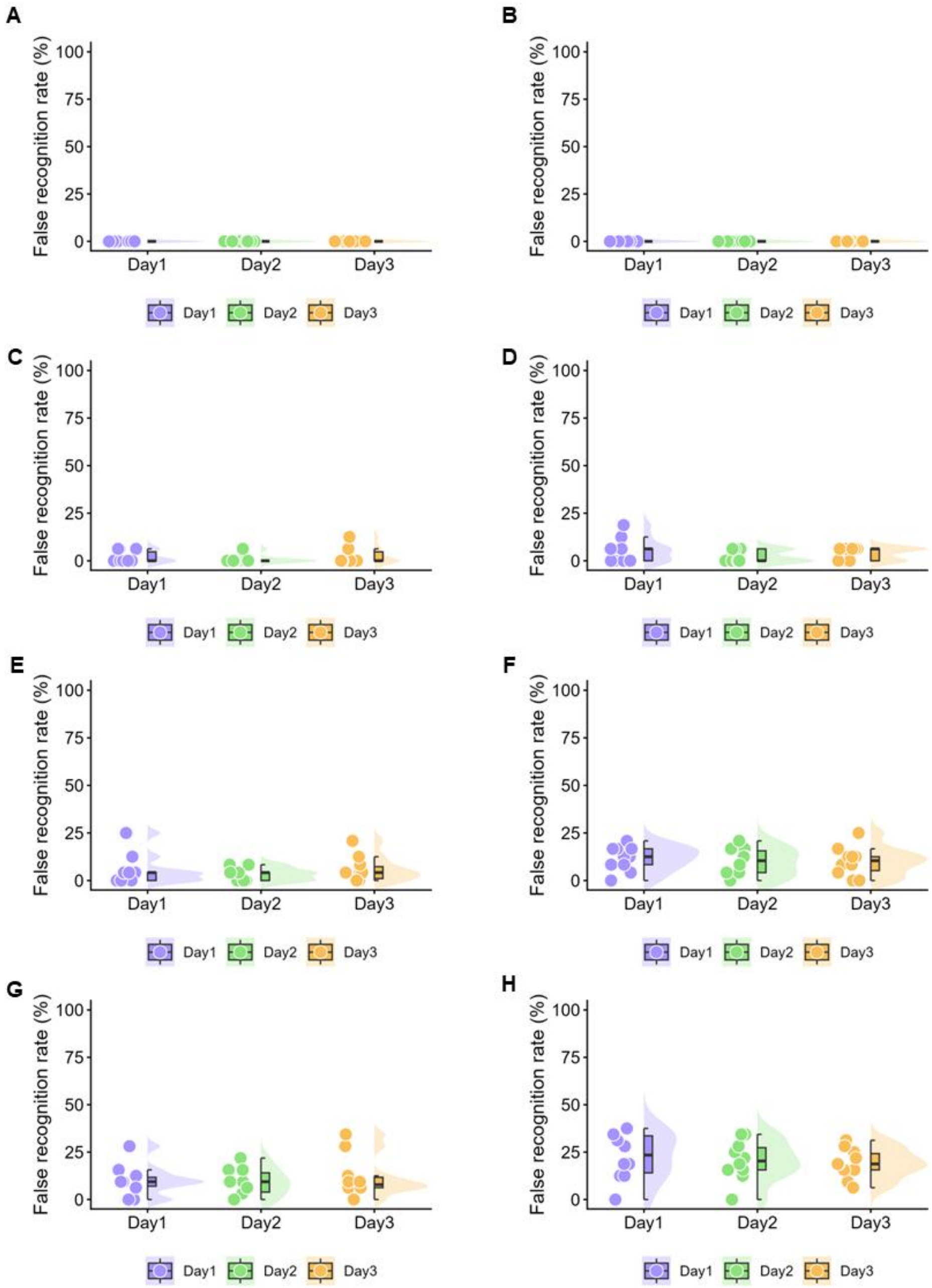

Fig. S7. Distributions of false non-target recognition rates for the tactile-encoded brain-computer interface (BCI) across increasing task complexity over three training days. Violin plots show participant-level false non-target recognition rates with embedded boxplots (n = 10 participants). The centre lines indicate the median values, box bounds represent the 25th and 75th percentiles, whiskers extend to the minimum and maximum values within 1.5 times the interquartile range. Rows correspond to increasing numbers of classifiable targets: (**A**, **B**) one target, (**C**, **D**) two targets, (**E**, **F**) three targets, and (**G**, **H**) four targets. Columns represent task conditions, with single-task (BCI-only) performance shown in the first column and dual-task performance (BCI combined with ball-balancing) shown in the second. A one-way repeated-measures ANOVA with a Greenhouse-Geisser correction was applied with main effects of training on false non-target recognition rates, followed by post hoc analysis with Bonferroni correction. No significant difference was found across days.

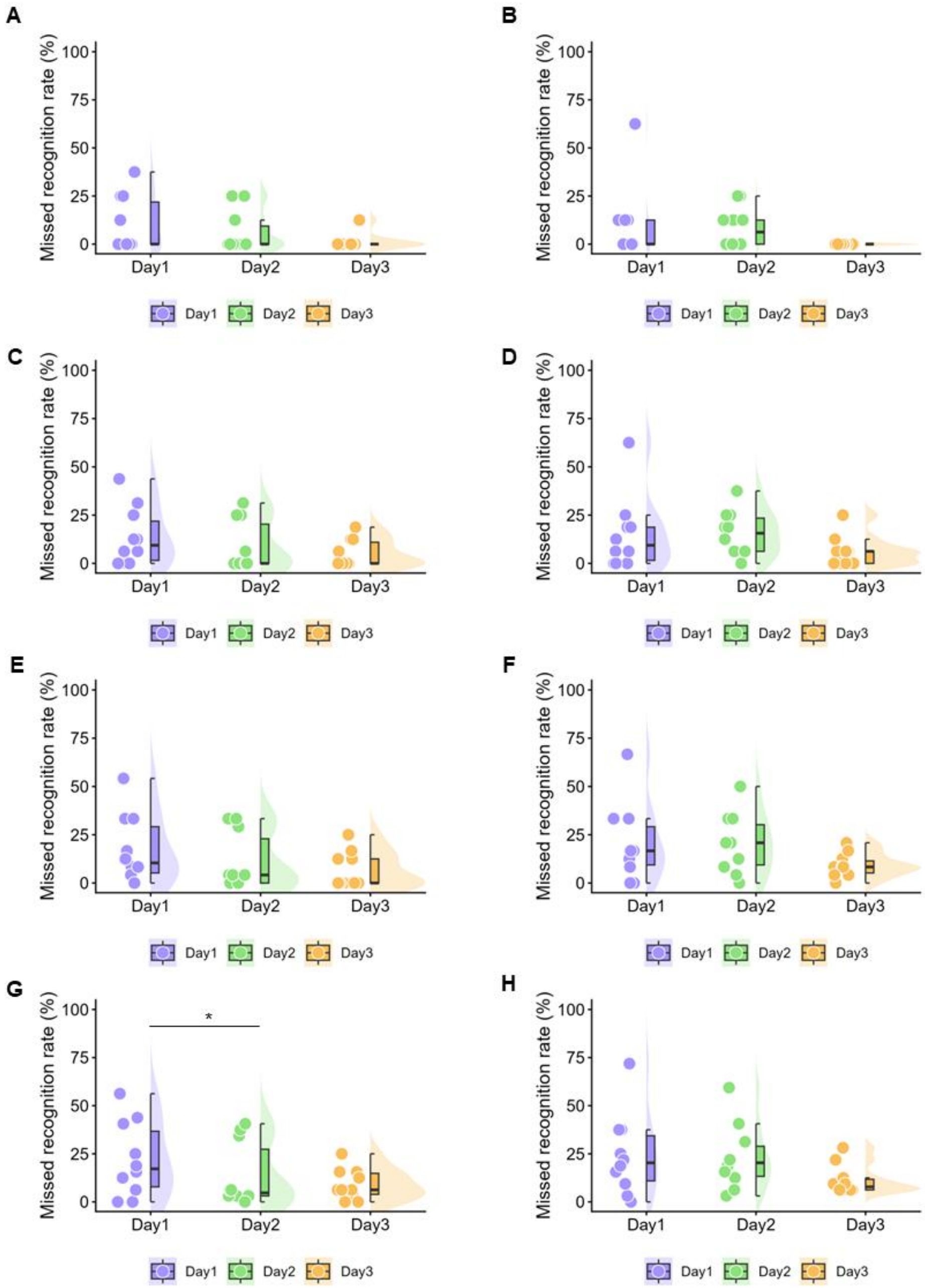

Fig. S8. Distributions of missed-recognition rates for the tactile-encoded brain-computer interface (BCI) across increasing task complexity over three training days. Violin plots show participant-level missed-recognition rates with embedded boxplots (n = 10 participants). The centre lines indicate the median values, box bounds represent the 25th and 75th percentiles, whiskers extend to the minimum and maximum values within 1.5 times the interquartile range. Rows correspond to increasing numbers of classifiable targets: (**A**, **B**) one target, (**C**, **D**) two targets, (**E**, **F**) three targets, and (**G**, **H**) four targets. Columns represent task conditions, with single-task (BCI-only) performance shown in the first column and dual-task performance (BCI combined with ball-balancing) shown in the second. A one-way repeated-measures ANOVA with a Greenhouse-Geisser correction was applied with main effects of training on missed-recognition rates, followed by post hoc analysis with Bonferroni correction. *P*-value: 0.026 (**G**, Day 1-Day 2). * if $p < 0.05$.

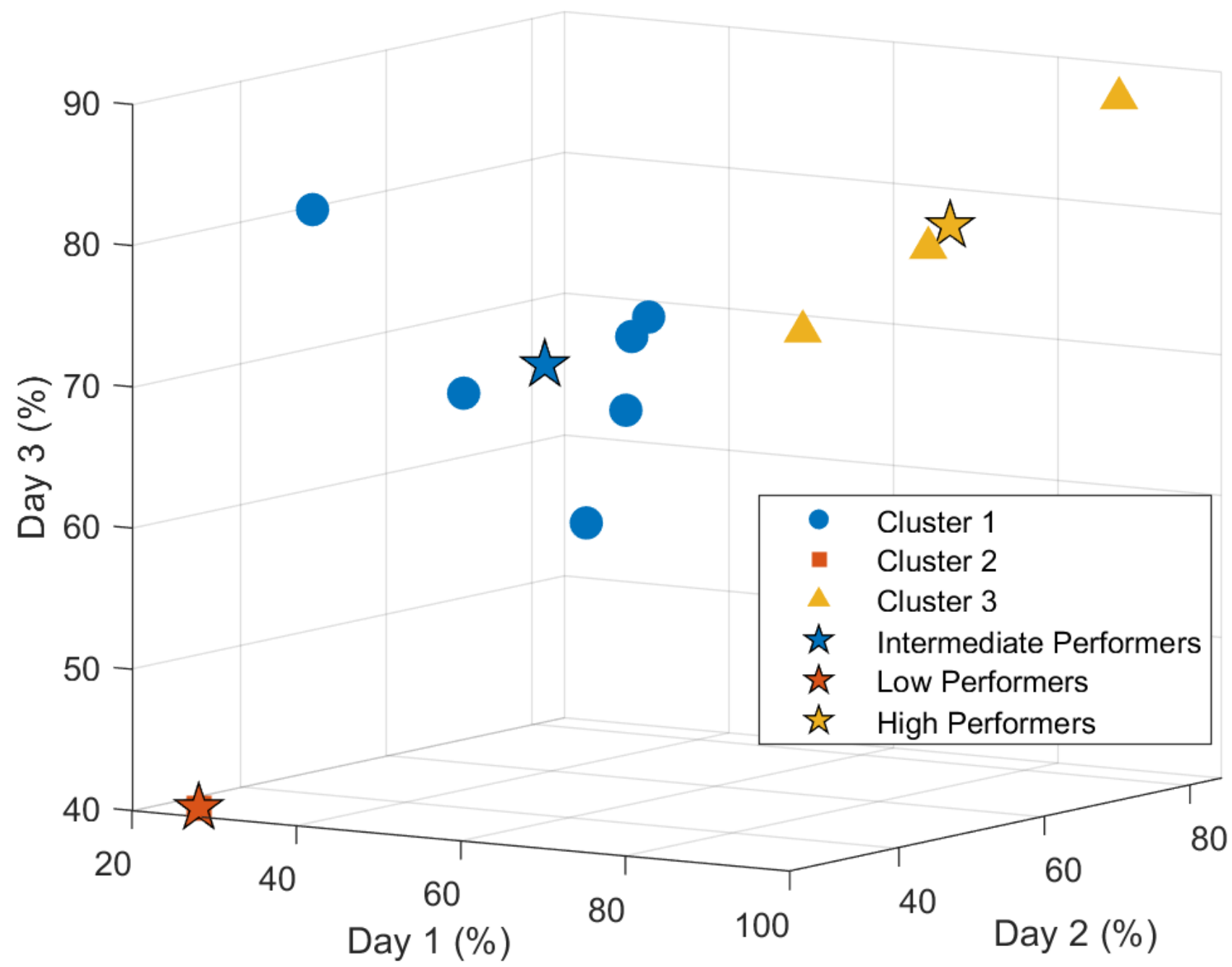

Fig. S9. Individual difference analysis using K-means clustering based on success rates of dual-task runs across days 1-3. Three-dimensional scatter plot shows the distribution of 10 participants' success rates on days 1, 2, and 3. Data points are color-coded by cluster membership (blue: Cluster 1, Intermediate Performer; red: Cluster 2, Low Performer; yellow: Cluster 3, High Performers).

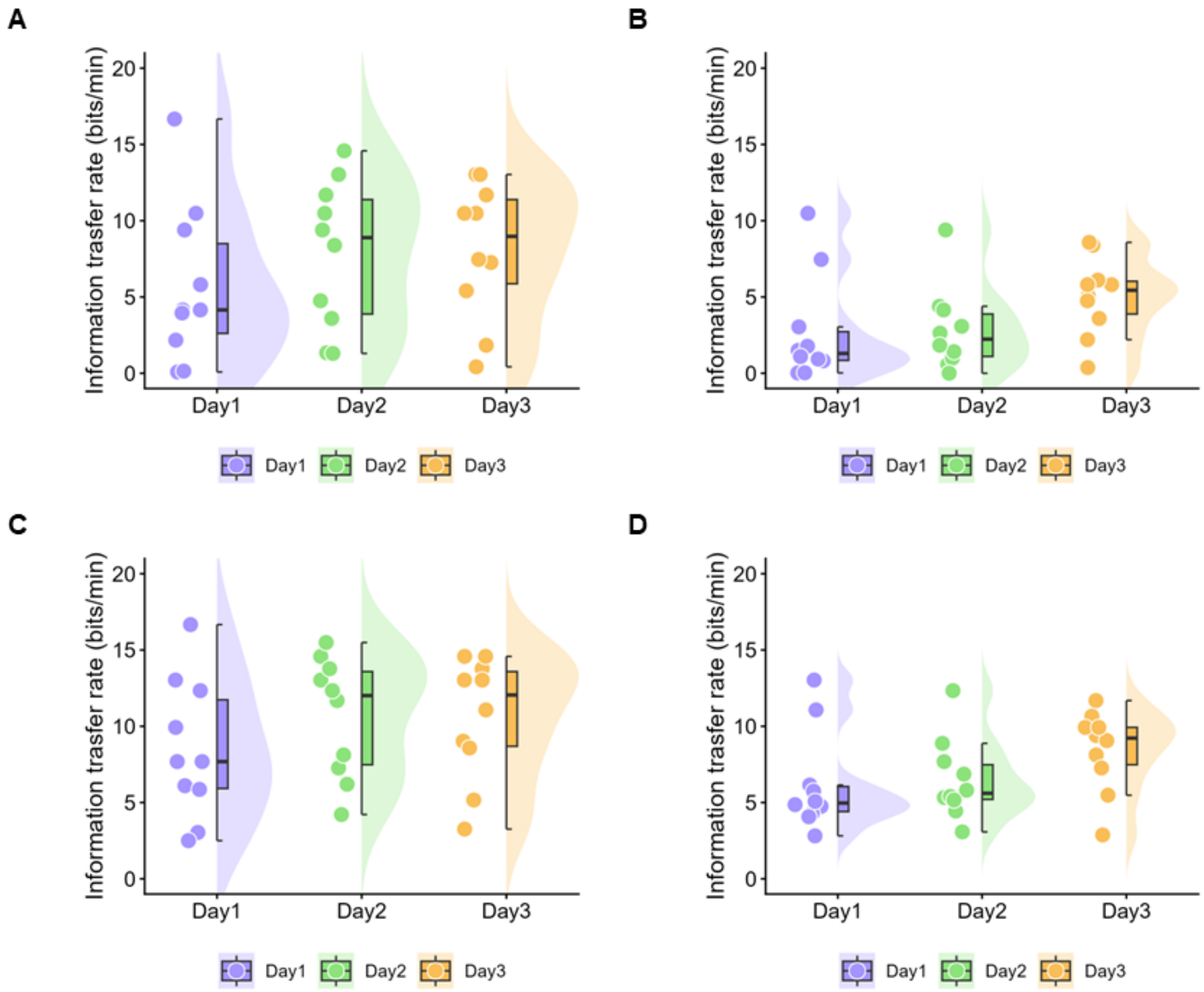

Fig. S10. Distributions of information transfer rate (ITR) for the tactile-encoded brain-computer interface over three training days. Violin plots show participant-level ITR with embedded boxplots (n = 10 participants). The centre lines indicate the median values, box bounds represent the 25th and 75th percentiles, whiskers extend to the minimum and maximum values within 1.5 times the interquartile range. ITR was calculated based on online success rate in (**A**) single-task and (**B**) dual-task conditions, and based on accuracy in (**C**) single-task and (**D**) dual-task conditions. A one-way repeated-measures ANOVA with a Greenhouse-Geisser correction was applied with main effects of training on ITR, followed by post hoc analysis with Bonferroni correction. No significant difference was found across days.

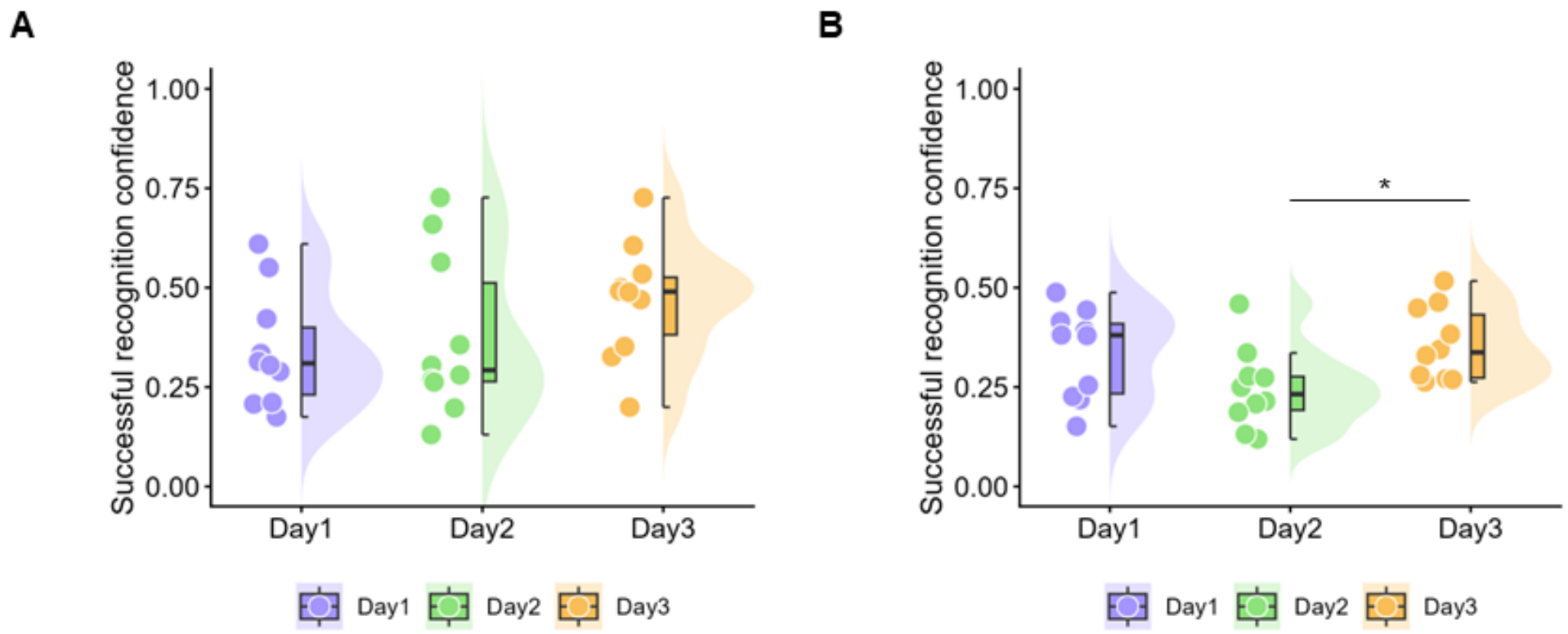

Fig. S11. Distributions of successful recognition confidence for the tactile-encoded brain-computer interface (BCI) over three training days. Violin plots show participant-level recognition confidence with embedded boxplots (n = 10 participants). The centre lines indicate the median values, box bounds represent the 25th and 75th percentiles, whiskers extend to the minimum and maximum values within 1.5 times the interquartile range. (**A**) Single-task and (**B**) dual-task conditions. A one-way repeated-measures ANOVA with a Greenhouse-Geisser correction was applied with main effects of training on successful recognition confidence, followed by post hoc analysis with Bonferroni correction. *P*-value: 0.004 (**B**, Day 2-Day 3). * if $p < 0.05$.

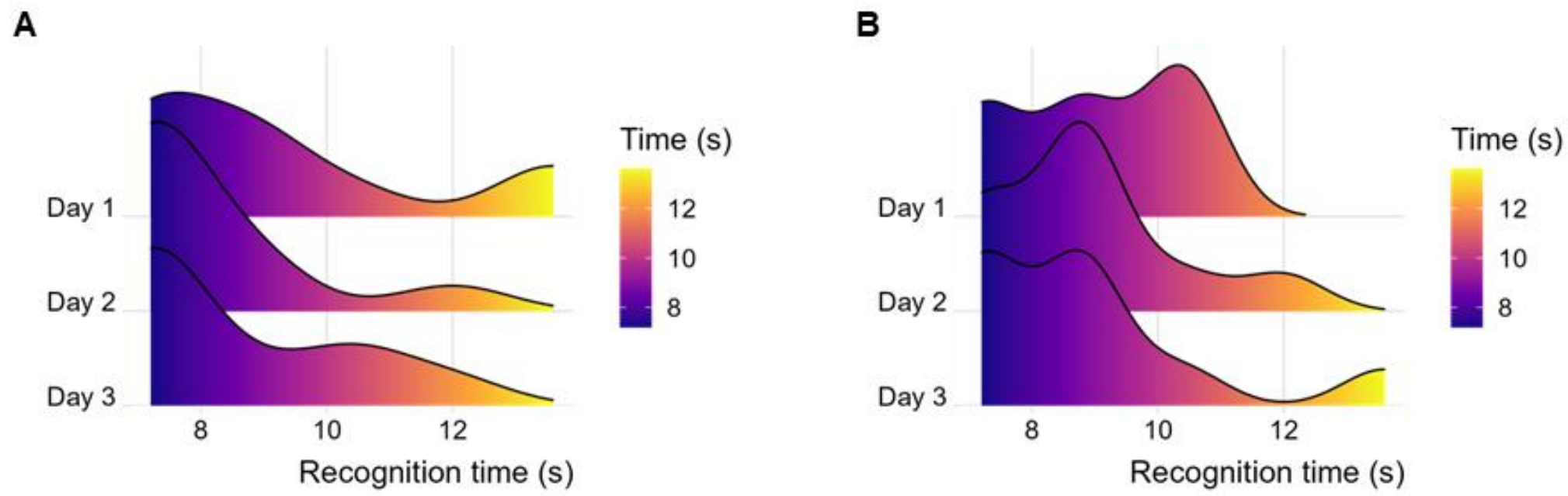

Fig. S12. Participant-level distribution of the correct recognition time (s) of the tactile-encoded brain-computer interface (BCI) over three days of training. (A) Single-task and **(B)** dual-task conditions (n = 10 participants).

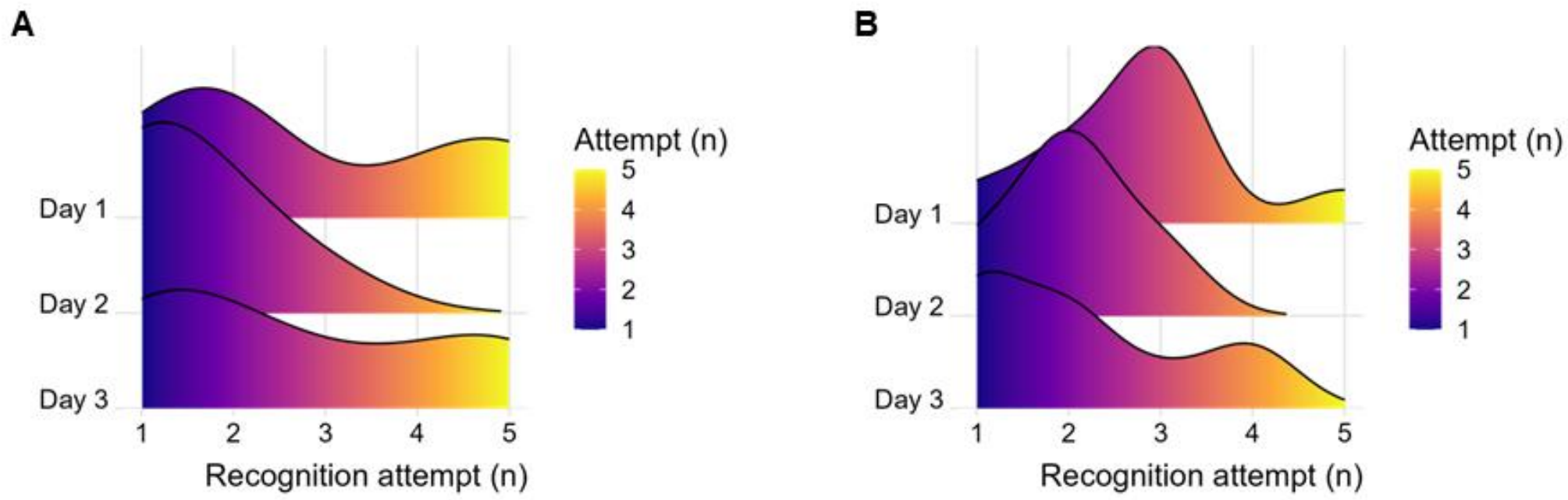

Fig. S13. Participant-level distribution of the number of recognition attempts before first false non-target recognition for the false positive runs of tactile-encoded brain-computer interface (BCI) over three days of training. (A) Single-task and **(B)** dual-task conditions (n = 10 participants).

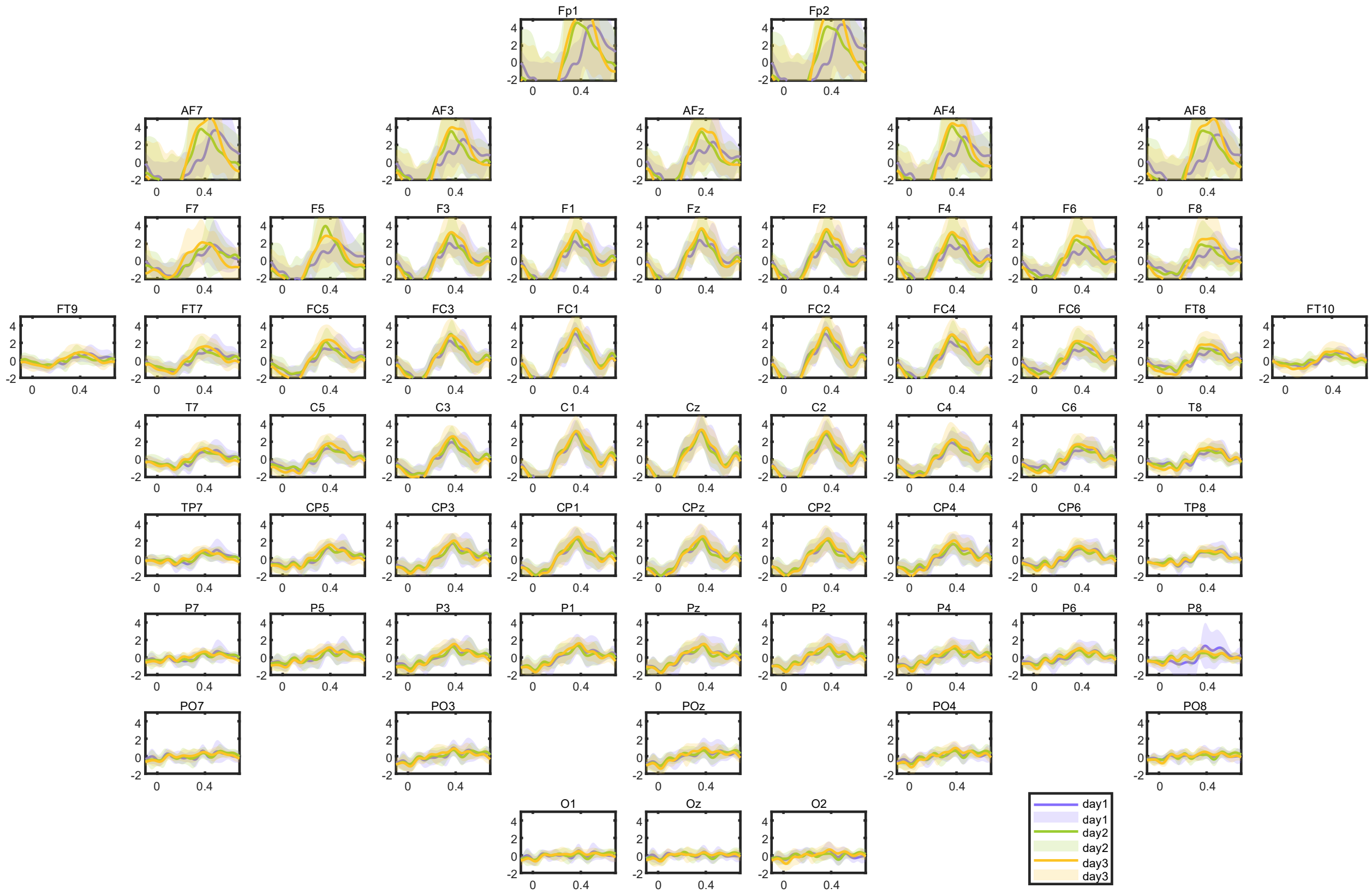

Fig. S14. Evoked P300 underlying the tactile-encoded brain-computer interface (BCI) tasks over three days of experiment across 10 participants for the single-task condition. The solid lines represent the mean amplitude of the P300, and the shaded boundary represents the standard deviation across participants.

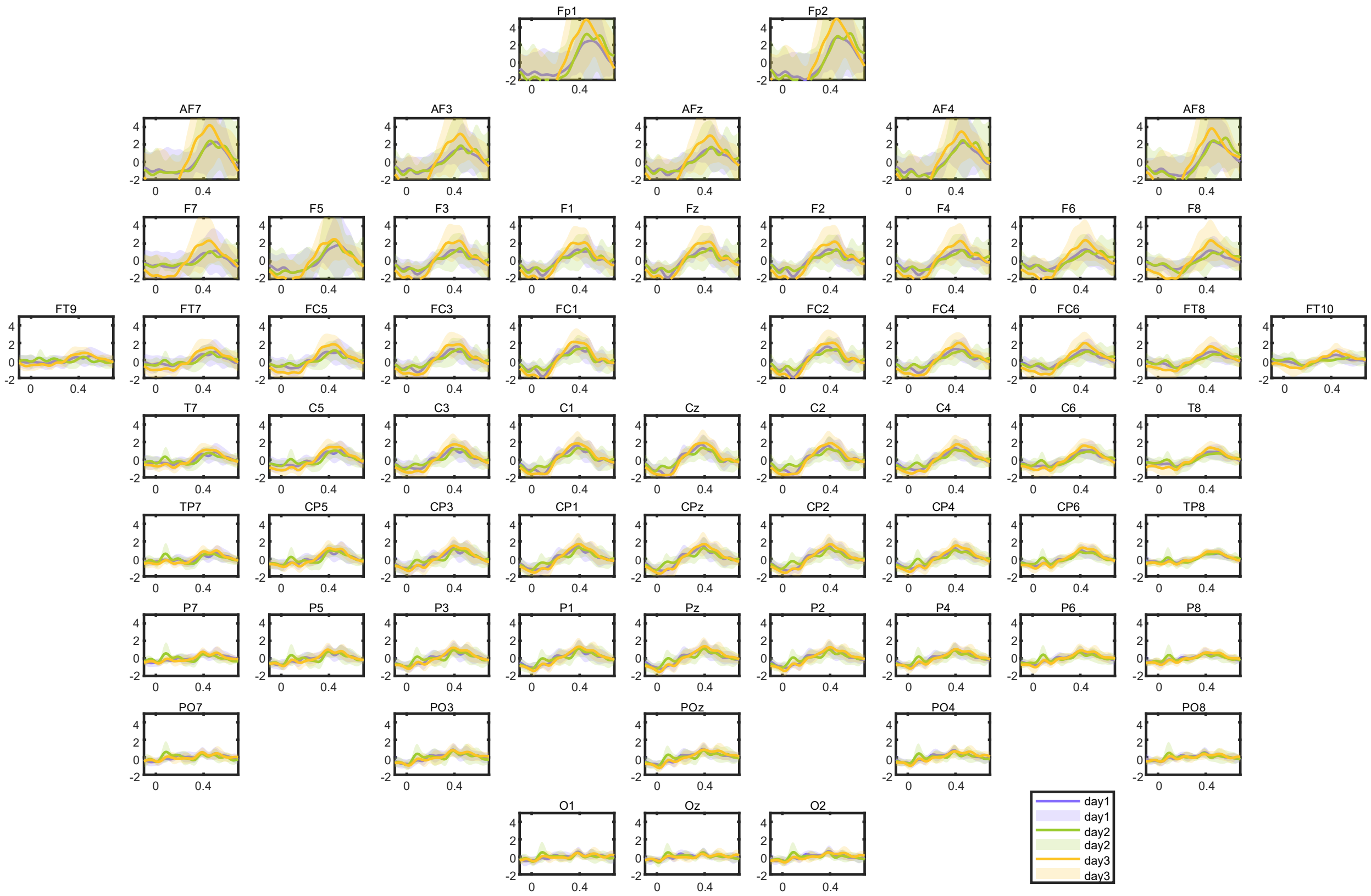

Fig. S15. Evoked P300 underlying the tactile-encoded brain-computer interface (BCI) tasks over three days of experiment across 10 participants for the dual-task condition. The solid lines represent the mean amplitude of the P300, and the shaded boundary represents the standard deviation across participants.

Table S1. Time intervals between experimental sessions for each participant, expressed in days relative to Day 1.

Participant	Day 2 (+ days)	Day 3 (+ days)
1	+2	+10
2	+7	+13
3	+1	+3
4	+4	+5
5	+8	+9
6	+7	+14
7	+8	+15
8	+1	+7
9	+1	+3
10	+3	+6
mean ± std	+4.2±3.0	+8.5±4.4

Table S2. Performance evaluation for the supplementary movie demonstrations.

Demonstration No.	Metrics	C1→C2	C2→C3
Movie S1	[1]Command-switch interval 1 (s)	11.2	N/A
	[2]Command-switch interval 2 (s)	8.2	N/A
	[3]Command-stop delay (s)	1.6	N/A
Movie S2	[1]Command-switch interval 1 (s)	16	27.2
	[2]Command-switch interval 2 (s)	8.3	12.6
	[3]Command-stop delay (s)	1.6	1.6
Movie S3	[1]Command-switch interval 1 (s)	12.8	11.2
	[2]Command-switch interval 2 (s)	4.8	5.6
	[3]Command-stop delay (s)	1.6	9.6
Movie S4	[1]Command-switch interval 1 (s)	3.2	4.8
	[2]Command-switch interval 2 (s)	2.1	3.7
	[3]Command-stop delay (s)	1.6	0

[1]Command-switch interval 1 refers to the interval from the first recognition of the previous command to the first recognition of the next command.
[2]Command-switch interval 2 is defined as Command-switch interval 1 minus the time taken by the robotic arm to execute the task.
[3]Command-stop delay refers to the duration of the delay before the recognition of the previous command concludes.

Movie S1. Demonstration of single-robotic arm control for a ball grasping task. In this demonstration, both Kinova Gen3 robotic arms first initialise to their predefined home poses. The user can then use the interface to command either Arm 1 or Arm 2 to perform two possible movements: (i) move to the pickup location, and (ii) grasp the ball and return to the home position. This added option increases task difficulty, as correct arm selection is required in addition to sequential command execution. The demo illustrates the principle of independent decoding and stepwise control, showing how discrete commands can be translated into movement and providing an example for more complex dual-arm coordination.

Movie S2. Demonstration of dual-arm coordination for a straw-cup drinking task. In this demonstration, both Kinova Gen3 robotic arms start from their home positions and respond to a sequence of three external control commands to complete a straw-cup drinking task. Responding to input from the neural interface, Arm 1 first moves to the cup pickup pose, grasps the cup, and brings it in front of the user. Next, Arm 2 moves to the straw pickup pose, grasps the straw, positions itself above the cup, and releases the straw into the cup. Finally, once the drinking action is completed, Arm 1 returns the cup to its starting location and places it down. This demonstration showcases sequential command execution and dual-arm coordination, where the actions of one arm complement the other to achieve a shared functional outcome.

Movie S3. Demonstration of dual-arm coordination with repeatable control. In this demonstration, both Kinova Gen3 robotic arms begin from the home position and execute a cyclic sequence of coordinated actions under repeated interface control signals. Arm 1 first receives a command to pick up a ball from the tray and return with it to the home pose. Arm 2 then executes a second command to transport the ball to the drop-off location before returning home. A third command, identical to the first, is subsequently sent to Arm 1, which repeats the pickup action to retrieve another ball and bring it back home. This demonstration illustrates dual-arm coordination with repeated task cycles, emphasising the system's ability to handle successive commands in the correct order.

Movie S4. Demonstration of continuous directional control of a single robotic arm on a two-dimensional plane. In this demonstration, a single Kinova Gen3 robotic arm is guided across a two-dimensional workspace using repeated directional commands decoded from the brain-computer interface (BCI). Four target stimuli are mapped to four planar movement directions (forward, backward, left, and right), enabling the user to progressively steer the end-effector. Accurate path following requires sustained attention and multiple successive decoding events, as the arm advances incrementally with each recognised command. This example demonstrates how discrete BCI outputs can be composed over time to support continuous spatial control of a robotic end-effector.
