# Supplementary material for: Concurrent control of natural and robotic limbs through a tactile-encoded brain-computer interface
Source: Nat Commun. 2026 Jul 2;17:8229. doi: 10.1038/s41467-026-75213-3 (PMC13462973; doi:10.1038/s41467-026-75213-3)
Supplement: Supplementary file 2 [file 41467_2026_75213_MOESM2_ESM.pdf]

Description of Additional Supplementary Files

Supplementary Movie 1. Demonstration of single-robotic arm control for a ball grasping task.

Supplementary Movie 2. Demonstration of dual-arm coordination for a straw-cup drinking task.

Supplementary Movie 3. Demonstration of dual-arm coordination with repeatable control.

Supplementary Movie 4. Demonstration of continuous directional control of a single robotic arm on a two-dimensional plane.
