# Supplementary material for: Concurrent control of natural and robotic limbs through a tactile-encoded brain-computer interface
Source: Nat Commun. 2026 Jul 2;17:8229. doi: 10.1038/s41467-026-75213-3 (PMC13462973; doi:10.1038/s41467-026-75213-3)
Supplement: Supplementary file 3 [file 41467_2026_75213_MOESM7_ESM.pdf]

nature portfolio

Corresponding author(s): Dario Farina

Last updated by author(s): Jun 1, 2026

Reporting Summary

Nature Portfolio wishes to improve the reproducibility of the work that we publish. This form provides structure for consistency and transparency in reporting. For further information on Nature Portfolio policies, see our Editorial Policies and the Editorial Policy Checklist.

Statistics

For all statistical analyses, confirm that the following items are present in the figure legend, table legend, main text, or Methods section.

n/a	Confirmed	
☐	☒	The exact sample size (*n*) for each experimental group/condition, given as a discrete number and unit of measurement
☐	☒	A statement on whether measurements were taken from distinct samples or whether the same sample was measured repeatedly
☐	☒	The statistical test(s) used AND whether they are one- or two-sided<br>*Only common tests should be described solely by name; describe more complex techniques in the Methods section.*
☐	☒	A description of all covariates tested
☐	☒	A description of any assumptions or corrections, such as tests of normality and adjustment for multiple comparisons
☐	☒	A full description of the statistical parameters including central tendency (e.g. means) or other basic estimates (e.g. regression coefficient) AND variation (e.g. standard deviation) or associated estimates of uncertainty (e.g. confidence intervals)
☐	☒	For null hypothesis testing, the test statistic (e.g. F, t, r) with confidence intervals, effect sizes, degrees of freedom and P value noted<br>*Give P values as exact values whenever suitable.*
☐	☒	For Bayesian analysis, information on the choice of priors and Markov chain Monte Carlo settings
☒	☐	For hierarchical and complex designs, identification of the appropriate level for tests and full reporting of outcomes
☐	☒	Estimates of effect sizes (e.g. Cohen's d, Pearson's r), indicating how they were calculated

Our web collection on statistics for biologists contains articles on many of the points above.

Software and code

Policy information about availability of computer code

Data collection	Electroencephalography signals were collected using actiCHamp Plus amplifier (Brain Products GmbH, Gilching, Germany) and MATLAB (2024a, The MathWorks Inc., Natick, MA, USA).
Data analysis	Data analysis was based on IBM SPSS Statistics 26, R 4.5.2, G*Power 3.1.9.7, and MATLAB (2024a, The MathWorks Inc., Natick, MA, USA). The codes for online EEG preprocessing, decoding as well as P300 signal plotting used in this study are available on GitHub at: https://github.com/jty940529/Tactile_P300_BCI.

For manuscripts utilizing custom algorithms or software that are central to the research but not yet described in published literature, software must be made available to editors and reviewers. We strongly encourage code deposition in a community repository (e.g. GitHub). See the Nature Portfolio guidelines for submitting code & software for further information.

Data

Policy information about availability of data

All manuscripts must include a data availability statement. This statement should provide the following information, where applicable:

- Accession codes, unique identifiers, or web links for publicly available datasets
- A description of any restrictions on data availability
- For clinical datasets or third party data, please ensure that the statement adheres to our policy

All data supporting the findings of this study are available within the article and its supplementary files. Any additional requests for information can be directed to,

and will be fulfilled by, the corresponding authors. Source data are provided with this paper. The data for this study are publicly available at https://doi.org/10.5281/zenodo.17306307.

Research involving human participants, their data, or biological material

Policy information about studies with human participants or human data. See also policy information about sex, gender (identity/presentation), and sexual orientation and race, ethnicity and racism.

Reporting on sex and gender	Ten able-bodied participants (six males, four females) volunteered for this study. Sex and gender were not variables of focus. Participants were not stratified or analyzed based on sex or gender. Sex-related differences were not among the research hypothesis.
Reporting on race, ethnicity, or other socially relevant groupings	No groupings by race, ethnicity, or socioeconomic status were performed.
Population characteristics	Ten able-bodied participants (six males, four females, aged between 23 and 37 years) volunteered for this study. All participants had no history of neurological disorders. No genotypic information or additional diagnostic categories were collected.
Recruitment	All able-bodied participants were recruited publicly at Imperial College London, London, UK.
Ethics oversight	This study was approved by the Imperial College Research Ethics Committees (ICREC reference 18IC4685). Prior to the experiment, participants received an explanation of the experiment procedure and provided signed informed consent. Potential video recording during the experiment for publications was also approved by all participants.

Note that full information on the approval of the study protocol must also be provided in the manuscript.

Field-specific reporting

Please select the one below that is the best fit for your research. If you are not sure, read the appropriate sections before making your selection.

☒ Life sciences ☐ Behavioural & social sciences ☐ Ecological, evolutionary & environmental sciences

For a reference copy of the document with all sections, see nature.com/documents/nr-reporting-summary-flat.pdf

Life sciences study design

All studies must disclose on these points even when the disclosure is negative.

Sample size	To evaluate whether the sample size (N = 10) was adequate to support the main findings in this study, we performed a post-hoc power analysis using G*Power. The analysis was conducted on the primary outcome measure: the online success rate for dual-task runs across days 1–3. The analysis revealed a large effect size, resulting in a statistical power exceeding 0.99 with the sample of 10 participants. This result demonstrates that the sample size is sufficiently large to detect the significant effects reported in this study.
Data exclusions	No data were excluded from the analysis.
Replication	Experiments were repeated across 10 participants and across 3 days. Each participant on each day underwent 32 repeated tests under both the single-task and dual-task conditions.
Randomization	As this study did not design experimental and control groups, no randomization was performed in participant grouping.
Blinding	As this study did not design experimental and control groups, no blinding was performed.

Reporting for specific materials, systems and methods

We require information from authors about some types of materials, experimental systems and methods used in many studies. Here, indicate whether each material, system or method listed is relevant to your study. If you are not sure if a list item applies to your research, read the appropriate section before selecting a response.

Materials & experimental systems

n/a	Involved in the study
☒	☐ Antibodies
☒	☐ Eukaryotic cell lines
☒	☐ Palaeontology and archaeology
☒	☐ Animals and other organisms
☒	☐ Clinical data
☒	☐ Dual use research of concern
☒	☐ Plants

Methods

n/a	Involved in the study
☒	☐ ChIP-seq
☒	☐ Flow cytometry
☒	☐ MRI-based neuroimaging

Plants

Seed stocks	No experiments related to plants.
Novel plant genotypes	No experiments related to plants.
Authentication	No experiments related to plants.
