# Supplementary material for: Concurrent control of natural and robotic limbs through a tactile-encoded brain-computer interface
Source: Nat Commun. 2026 Jul 2;17:8229. doi: 10.1038/s41467-026-75213-3 (PMC13462973; doi:10.1038/s41467-026-75213-3)
Supplement: Supplementary file 4 [file 41467_2026_75213_MOESM8_ESM.pdf]

nature portfolio

Peer Review File

Concurrent control of natural and robotic limbs through a tactile-encoded brain-computer interface

Corresponding Author: Professor Dario Farina

This file contains all reviewer reports in order by version, followed by all author rebuttals in order by version.

Version 0:

Reviewer comments:

Reviewer #1

(Remarks to the Author)
This paper investigates whether a tactile-evoked P300 can serve as a practical control channel for supernumerary robotic degrees of freedom during human movement augmentation. The core idea is ingenious: the authors use vibrotactile stimulation in an oddball-style paradigm, decode the participant's (10 consented through approved ethics framework) attention-driven P300 response from EEG, and translate that into discrete robot commands (specifically selecting among actions for assistive robotic arms). A central emphasis is that this channel can be robust, quick to calibrate, and minimally disruptive because it does not rely on muscle activation (which could interfere with natural movement) nor on intensive visual attention (which could compete with the user's visual workload). The study evaluates single-BCI versus dual-task conditions across multiple training days to test stability and whether performance improves with practice while the user simultaneously performs natural motor tasks.

This paper is very well written and the data are compelling. Most of the recommended comments and edits I have are straightforward to address, and I hope they will be constructive to the authors. One note of caution is that the framing around "supernumerary DoFs" would benefit from more precise language—what's demonstrated is centered around discrete supervisory command selection, not exactly continuous multi-DoF control. The last part of the discussion section goes into this, however the 'continuous' signal is essentially a time-continuous confidence/detection of a class, not a direct decode of continuous amplitude levels that would naturally map to proportional DoF control. As for the potential for training-related performance gains, it would be useful to more directly connect improvements to measurable factors beyond peak amplitude/latency (e.g., classifier confidence, ITR/throughput, single-trial SNR, artifact rates, or trial-to-trial variability), and avoid metrics that are somewhat orthogonal, such are CV, which is an indicator of dispersion, and leptokurtosis, which should be supported by kurtosis results and is an indicator of the behavior at the tails. Having worked on robotic applications, I would also like the authors to explicitly address if there were any safety constraints associated with the robotic motion for safety purposes: it sounds like if there were, they didn't influence the results, but it would be good to be explicit about this (possibly in the supplementary information). Finally, a brief discussion on how tactile adaptation might affect long-duration, real-world use outside the lab.

In fig. 1, the first vignette on the bottom right is blank. I'd have all 4 commands here, i.e., add the yellow bracelet version of this.

Reviewer #2

(Remarks to the Author)
I co-reviewed this manuscript with one of the reviewers who provided the listed reports. This is part of the Nature Communications initiative to facilitate training in peer review and to provide appropriate recognition for Early Career Researchers who co-review manuscripts.

Reviewer #3

(Remarks to the Author)
This study proposes a novel paradigm for neural interface-based movement augmentation and presents highly encouraging findings. The proposed tactile-encoded BCI is an efficient solution for addressing the key challenge in BCI research of

directly decoding superimposed neural activities. By using tactile encoding, this BCI system significantly expands the controllable DoFs for movement augmentation. The online experiment results confirm that tactile-encoded BCI control can operate simultaneously with the user's natural movement and exhibits a learning effect, which is compelling and encouraging. The functional task validation is also impressive. From my point of view, this study is rigorously designed, comprehensively validated, and presents clear interpretation of the results. I recommend acceptance for publication in Nature Communications after moderate revisions. Below are a few suggestions that could further strengthen the manuscript:

(1) The Introduction part can be further expanded to include state-of-the-art in BCI for augmentation by extension. Incorporating several key recent studies would help readers more clearly understand the recent advances, current research level and the specific challenge this study aims to address.

(2) In Figure 1, the P300 component is labeled as "sensory-evoked." If I understand correctly, I think that "sensory-encoded" or "attention-evoked" may be more accurate. Sensory input should be also present for non-target stimuli but P300 is not evoked when the participant does not allocate attention to such stimuli. I therefore suggest that the authors reconsider which label is more appropriate.

(3) The performance evaluation of the ball-balancing task serves as essential evidence supporting the concept of movement augmentation. However, the reasons for selecting the two evaluation metrics presented in Figure 4, as well as the detailed calculation methods, are not clear enough. I recommend adding a brief explanation to clarify this part.

(4) I notice that the Bayes factor of the time per successful rotation on Day 1 is over commonly-used standard line. While the null effects can be supported following training, the results on Day 1 still need appropriate explanation.

(5) I notice that the decoding of P300 relied on commonly-used algorithms. The authors could consider adding an appropriate discussion on advances in P300 recognition algorithms. This would provide valuable references for researchers who are inspired by this work and wish to further investigate this novel paradigm. For motor imagery-based BCIs, it has been reported that a certain proportion of subjects are BCI illiteracy. For the novel BCI paradigm proposed in this study, have similar phenomena been observed in the experiments? I suggest adding the discussion on individual performance differences.

(6) The online performance demonstrated significant improvement after training. Notably, a significant performance gain was sustained from Day 2 to Day 3, which may indicate that the dual-task performance has not yet reached its ceiling. It is recommended that the authors include discussion regarding this potential performance ceiling.

(7) Please define the abbreviation before its first use, such as "SNR". Please ensure all abbreviations used in the manuscript follow this convention. Please standardize the spelling throughout the manuscript. For instance, both the British English variant "artefacts" and the American English variant "artifacts" are used in the manuscript to denote EEG noise.

Reviewer #4

(Remarks to the Author)
The paper presents an intriguing study on movement augmentation through the concurrent control of supernumerary robotic DoFs via tactile P300-based BCIs. The authors successfully demonstrated that controlling four additional DoFs is possible without compromising natural bimanual object manipulation. The study advances the field by extending from single to multi-DoF augmentation, and, crucially, doing so without interfering with natural limb movement. The results enhance the plausibility of applying BCIs for movement augmentation in able-bodied people.

While the data support key findings of the study and overall experimental designs and analytic methods are solid, there remain several critical issues in the manuscript that should be addressed further to bolster the study' rigor.

Major comments
- Choice of sensory modality. It is critical to clarify why tactile modality is chosen over auditory modality. The suggested movement augmentation paradigm could also be achieved by auditory P300-based BCIs. Perhaps, auditory stimuli can offer a more independent control channel than tactile stimuli because of innate coupling between tactile perception and arm movement. But tactile modality may have other advantages, too. So, please provide rationale to choose tactile modality in this study.
- Sample size. The number of participants (N=10) in the current study appears to be too small to validate statistical significance of the results. Please assess the number of participants (sample size) to achieve meaningful statistical power using appropriate measures (e.g., G*Power) and increase the sample size up to a desired figure by additionally recruiting participants.
- Sense of embodiment. One of the critical points in movement augmentation is the emerging sense of embodiment. It would be important to find whether the sense of embodiment emerged or increased by training. Did the authors perform post-hoc surveys to assess subjective experiences of controlling supernumerary DoFs?
- Effect of training on neural responses. In Fig 2F, CV of P300 latency was similar between single- and dual tasks. Authors claimed that this evidences the training effect on neural responses. But no difference between the tasks could be due to increases in CV of single task. In that sense, the result of Fig 2F alone just partially supports the training effect on P300. Instead, the main effect of TIME (day) on CV of P300 latency followed by post-hoc comparisons (as in Fig 3B) should be done to fully support the authors' claim.

- Neural substrates of decoding performance improvement. Changes in P300 characteristics over training, implying training effects, are analyzed only at Cz (line 520). But decoding involves all channels except for 18 frontal ones, as far as I understand. So, using single-channel P300 characteristics to explain improvement in decoding performance would not be sufficient. It is recommended to explore P300 characteristics at all channels involved in decoding to illuminate neural substrates of decoding improvement.
- Natural movement performance evaluation. Since a major contribution of this study is to show that augmented movement does not compromise natural movement, the assessment of natural movement would be crucial. The authors employed the time per ball rotation as a measure of natural movement performance. But why was this measure used? It appears that participants performed the ball balancing task at their own pace. So, it may suggest that individual variations in the time per ball rotation within a condition would be as big as variations across conditions, yielding no statistical difference across conditions (Fig. 4). Since no instruction was given to perform the ball balancing task as quickly as possible, using the time per ball rotation only might not be sufficient. Rather, the authors may consider adding other performance measures. One example can be a success rate of ball rotation.
- Analysis of individual differences. It is essential to analyze individual differences. For instance, there can be good performers versus poor performers. If so, knowing what contributed to poor performance would be important. For instance, the analysis of EEG data to assess attentional load in individuals might be helpful.
- BCI performance metrics. One of the BCI performance metrics is the response time. But in fact, this metric counts the number of attempts until the target P300 was detected. It is more natural to measure the response time as the time elapsed until the target P300 was detected in the unit of seconds. For instance, if I understand correctly, when n = 1, the target P300 was detected at the first attempt after the fourth round, which would take 6.4 seconds. Then, the response time should be 6.4 seconds if we ignore the time taken for decoding. Accordingly, while the authors claim that reliable movement augmentation could be achieved with minimal latency (line 376), the minimal latency actually spans 6.4 – 8.0 seconds (first or second attempts), which seems not to be short.

Minor comments
- In the title and one-sentence summary, it would be recommended to change “neural interface” to “brain-computer interface” as neural interfaces can describe a broader range of systems than the proposed P300-based BCI.
- Line 60-62. Current limitations are not clear and need rewriting. Do these illustrate limitations of augmentation by extension via non-invasive signals? How come does redundancy in muscle activity lead to limited effectiveness and difficulties in control? Please consider revising the text.
- Line 70-72. Since SMRs are well known, it may not be necessary to explain SMRs in details. Consider removing this part.
- Line 78-82. “While MI offers a reliable source of multidimensional neural signals that can be internally modulated, its application in human augmentation is impractical as it requires deciphering supernumerary control intentions that are superimposed on the neural activity associated with natural movement.” But Bashford et al. (JNE 2018) demonstrated that humans could learn to dissociate cortical signals associated with the control of natural limb movements and the control supernumerary effectors. Hence, would it be possible for humans to modulate SMRs for both SE control and natural limb control via learning? Please discuss this possibility.
- Line 85-94. Explanation of the P300 speller is too long. This part can be shortened.
- Please provide rationale to choose body locations for tactile stimulation.
- Line 125. Is it correct to say that tactile P300 BCI is used to recognize motor intention? In fact, this description is found across the manuscript. I agree that the tactile P300 BCI in this study is used for recognizing the user’s intention of robot control. But motor intention is somewhat different, typically meaning the intention of movement encoded in relevant EEG signatures, not P300 per se. So, using the term, “motor intention” in tactile P300 BCIs can be misleading. Please consider revising the corresponding descriptions.
- Line 126-127. Another important question of this study would be whether the tactile-encoded BCI interferes with natural movement. Consider adding this question.
- Was the mapping between stimuli and commands randomized across participants? Please clarify.
- The P300 amplitudes in Fig. 2A are near 4 uV while those for the single task in Fig. 2B are around 7uV. Please explain this discrepancy.
- Please indicate the sample size inside Fig. 2 or in the caption.
- Please indicate the EEG channel for Fig. 2A, D in the caption.
- What do dots in Fig. 2G-H represent?
- In Fig. 2A, D, EEG channels showing high amplitudes in the topology are distributed in the frontocentral middle area. Typically, Pz is a key channel eliciting P300. Please discuss why Pz exhibits relatively low amplitudes in this study.
- Line 164-165. “the comparable P300 latency observed between single- and dual-BCI tasks on the last training day essentially revealed that both tasks exhibit similar performance after training.” The comparable P300 latency between the two tasks was observed on all training days (Fig. 2E). So, why this result reflect similar performance after training?
- In Fig. 3D-E, the distribution of the number of attempts extends to 0. Although these distribution curves may be obtained by smoothing histograms (I am not sure though because no explanation is given), zero attempt does not make a sense. So, consider drawing histograms instead or modifying the distribution curves.
- Line 282-284. “By combining the evoked P300 with prior tactile encoding, the proposed BCI augmented the controllable DoFs as many as the number of tactile vibrators, …” I am not sure about this. There are four vibrators but the number of commands (controllable DoFs) is three (see Fig. 1). Did the authors count “do nothing” as an independent controllable DoF? If so, please explain it.
- Line 479-481. It read that the classification of ERPs in response to each stimulus was conducted individually, yielding four classification outcomes. Then, it is possible that more than one outcome can be the detection of P300. In such cases, how to decide which stimulus was a target?
- Line 485-486. “The number of recognition attempts required until the target P300 was detected was recorded as the response time.” But in an unsuccessful test, P300 would be elicited by non-target stimuli. In this case, the response time was

not recorded? In other words, the response time was recorded only for successful tests? Then, what was the average response time for unsuccessful tests with false non-target recognition?
- Since there could be two cases when the test failed, including false non-target recognition and missed recognition, it is essential to report the rates of each failure separately.
- Why only four out of ten participants performed the additional test for continuous decoding? Why not everyone?
- What was the maximum number of attempts? Since there could be 80 trials per run and one round consisted of 4 trials, there could be as many as 20 rounds. Since the first attempt began after the 4th round, the maximum number of attempts would be 17? Please indicate it.
- What was the average time interval between experimental sessions (days)?
- Line 520-522. Channel Cz was chosen for the P300 analysis. Then, is it necessary to show the topographies in Fig. 2A, D?
- 18 EEG channels in the frontal regions were excluded from the P300 analysis. Were these channels also excluded from decoding? But, in Fig. 2A, D, frontal channels showed the largest P300 amplitudes. So, these channels needed to be included in decoding. Please clarify which channels were excluded from the P300 analysis and decoding, respectively.
- It is recommended to report the performance evaluation results for the four-limb coordination tasks, even though these tasks are designed for the demonstration purpose.
- Reporting the BCI performance in terms of the information transfer rate (ITR) would be helpful to compare the performance with existing tactile P300 BCIs.

Version 1:

Reviewer comments:

Reviewer #1

(Remarks to the Author)
I appreciate the edits to the manuscript made by the authors and have no further questions nor suggestions.

Reviewer #2

(Remarks to the Author)
I co-reviewed this manuscript with one of the reviewers who provided the listed reports. This is part of the Nature Communications initiative to facilitate training in peer review and to provide appropriate recognition for Early Career Researchers who co-review manuscripts.

Reviewer #3

(Remarks to the Author)
The authors have fully responded to all the comments.

Reviewer #4

(Remarks to the Author)
I truly appreciate the authors' tremendous efforts in revising the manuscript. I believe the manuscript has now improved significantly. While the authors have thoroughly and satisfactorily addressed all the comments I raised, I would like to suggest a few minor additional points as follows.

- Regarding the sense of embodiment, I fully understand that recall bias can affect the results of the survey conducted six months after the original experiment and that it would therefore not be appropriate to include these data in the manuscript. However, as the sense of embodiment may present an important question in the context of movement augmentation, I would suggest mentioning it as a topic for future investigation in the Discussion, so that readers are made aware of this open issue.
- Since Figures R2 and R3 are indeed informative regarding the ERP responses underlying the proposed BCI, I would suggest including them in the Supplementary Materials.
- Regarding the mapping between stimuli and commands, it is now clear that the mapping was kept consistent across participants, which is entirely appropriate. However, I still do not fully understand how keeping the consistent mapping across participants "facilitates learning across sessions". It is clear that keeping a certain mapping within a given participant across sessions would facilitate learning for that individual. What remains unclear is why keeping a consistent mapping across participants would facilitate learning across sessions within each participant.

Open Access This Peer Review File is licensed under a Creative Commons Attribution 4.0 International License, which permits use, sharing, adaptation, distribution and reproduction in any medium or format, as long as you give appropriate credit to the original author(s) and the source, provide a link to the Creative Commons license, and indicate if changes were made.
In cases where reviewers are anonymous, credit should be given to 'Anonymous Referee' and the source.
The images or other third party material in this Peer Review File are included in the article's Creative Commons license, unless indicated otherwise in a credit line to the material. If material is not included in the article's Creative Commons license and your intended use is not permitted by statutory regulation or exceeds the permitted use, you will need to obtain permission directly from the copyright holder.
To view a copy of this license, visit https://creativecommons.org/licenses/by/4.0/

Response to Reviewers' Comments

Nature Communications

Manuscript ID: NCOMMS-26-004391

Concurrent control of natural and robotic limbs through a tactile-encoded brain-computer interface

Tianyu Jia, Xingchen Yang, Ciaran McGeady, Yifeng Li, Jinzhi Lin, Kit San Ho, Feiyu Pan, Linhong Ji, Chong Li, Dario Farina

To Editors and Reviewers

Thank you for giving us the opportunity to submit a revised version of our manuscript entitled "Concurrent control of natural and robotic limbs through a tactile-encoded brain-computer interface" for potential publication in *Nature Communications*. We are grateful for the positive feedback and insightful comments to our manuscript. We appreciate the time and effort the editor and the reviewers dedicated to review our manuscript. We have incorporated all the suggestions made by the reviewers. The corresponding changes are highlighted within the manuscript. Please see below, for a point-by-point response to the comments of the four Reviewers.

Reviewer #1

The Reviewer's Comment #1

One note of caution is that the framing around "supernumerary DoFs" would benefit from more precise language—what's demonstrated is centered around discrete supervisory command selection, not exactly continuous multi-DoF control. The last part of the discussion section goes into this, however the 'continuous' signal is essentially a time-continuous confidence/detection of a class, not a direct decode of continuous amplitude levels that would naturally map to proportional DoF control.

Response:

Thank you for this helpful comment. We agree that precise language is important for conveying our work. To reduce the ambiguity surrounding the term "supernumerary DoF," we have added the word "discrete" to clarify that the proposed control is not linked to the continuous amplitude of an endogenously modulated neural signal. The revised sentence now reads:

> "We hypothesise that the tactile-evoked P300 could serve as an effective signal for discrete control of artificial supernumerary DoFs for human movement augmentation."

Based on your comment, we have also revised the final paragraph of the Discussion to focus on *continuous* rather than *proportional* control; the latter is defined as control in which the output of the command interface scales with the magnitude of the neural control signal.

As noted in the paragraph, such *proportional* control is not feasible with P300 decoding. Instead, we propose a method in which sustained generation of the P300 signal is interpreted by the command interface as a continuous control command.

> "Accordingly, it is difficult to design neural feedback that would allow subjects to voluntarily modulate P300 characteristics for proportional control. To address this limitation and aim at continuous P300 control, we proposed to replace the modulation of P300 characteristics with a continuous detection of P300. This approach translates the sustained, uninterrupted detection of P300s into a continuous control signal (e.g., Movie S4: moving a robotic arm between two positions) rather than event-based commands, allowing users to navigate the supernumerary workspace."

The Reviewer's Comment #2

As for the potential for training-related performance gains, it would be useful to more directly connect improvements to measurable factors beyond peak amplitude/latency (e.g., classifier confidence, ITR/throughput, single-trial SNR, artifact rates, or trial-to-trial variability), and avoid metrics that are somewhat orthogonal, such are CV, which is an indicator of dispersion, and leptokurtosis, which should be supported by kurtosis results and is an indicator of the behavior at the tails.

Response:

We agree that from the perspective of robotic control, the metrics you mentioned, such as ITR and classifier confidence, are indeed more direct and meaningful indicators of practical system performance. Following your recommendation, we have added Figures S10 and S11 to the revised manuscript to show the ITR and classifier confidence respectively to provide a more comprehensive and functionally relevant evaluation of BCI control performance. ITR was computed based on the four-degree-of-freedom online recognition success rates reported in Figure 3A and B, together with the corresponding recognition time (in terms of recognition attempt reported in Figure 3D and E). We would like to highlight an important point regarding the ITR calculation: we used the online success rate, rather than the classification accuracy, as the basis for computing ITR. This is because the online task involves the recognition of four degrees of freedom, and while the theoretical classification accuracy must be higher, the success rate more faithfully reflects the functional performance of the system in a real-time control context. To ensure consistency and mutual validation across all reported results, we deliberately adopted the online success rate for ITR estimation. To facilitate comparisons of the results in this study with other existing studies, the ITR calculated based on classification accuracy results have also been included.

While we have added new metrics ITR and classifier confidence, we would prefer to also keep the previously provided coefficient of variation (CV) metrics. We indeed believe that trial-to-trial variability is an important metric when evaluating P300 responses. In this study, we used CV to quantify such variability. Because our classification approach relies on the

consistency of P300 features, particularly the stability of amplitude and latency over time, a robust feature space is important for performance. The coefficient of variation was used to quantify this potentially consistency across days. A lower CV indicates that the P300 responses are more consistent across time. We expected that higher CV values would be more commonly observed in naïve users and would decrease with training, as participants become more familiar with the experimental paradigm. The use of this statistical metric is motivated by previous work in event-related potential analysis. For example, Perez et al. (2017) compared CV values between pure tone and speech stimuli in an auditory-evoked P300 BCI paradigm, reporting higher accuracy for speech stimuli, which was attributed to lower CV (i.e., reduced relative signal dispersion and improved stability). Molina et al. (2024) also used CV to analyse trial-level dispersion of P300 components. We have added reference to this paper. We believe this addition strengthens the connection between neural signal improvements and the actual usability of the BCI system.

Having added the suggested additional metrics, we realise that, as you mention, concluding a learning effect in BCI control based solely on changes in CV of P300 latency would be overly strong, as BCI learning effects are likely driven by multiple factors beyond this single measure. Accordingly, in the revised manuscript, we have also modified other corresponding text to temper the original conclusion.

"The decoding performance of the BCI relies on distinguished features, such as the higher P300 peak amplitude and their greater similarity across trials. To quantify this similarity, we introduced CV as a metric to evaluate the trial-to-trial variability *(32, 33)*. Lower CV values indicate a leptokurtic distribution, reflecting greater consistency in P300 responses across trials, which may enhance decoding performance.

However, it was not possible to infer a learning effect in BCI control solely from changes in the CV of the P300 latency, especially because other metrics of performance, such as information transfer rate (ITR), did not fully correlate with CV.

References:

32. M. Molina, L. J. Tardón, A. M. Barbancho, I. De-Torres, I. Barbancho, Enhanced average for event-related potential analysis using dynamic time warping. *Biomed. Signal Proces.* 87 (2024).
33. A. P. Perez, K. Ziliotto, L. D. Pereira, Test-retest of long latency auditory evoked potentials (P300) with pure tone and speech stimuli. *Int. Arch. Otorhinolar.* 21, 134-139 (2017)."

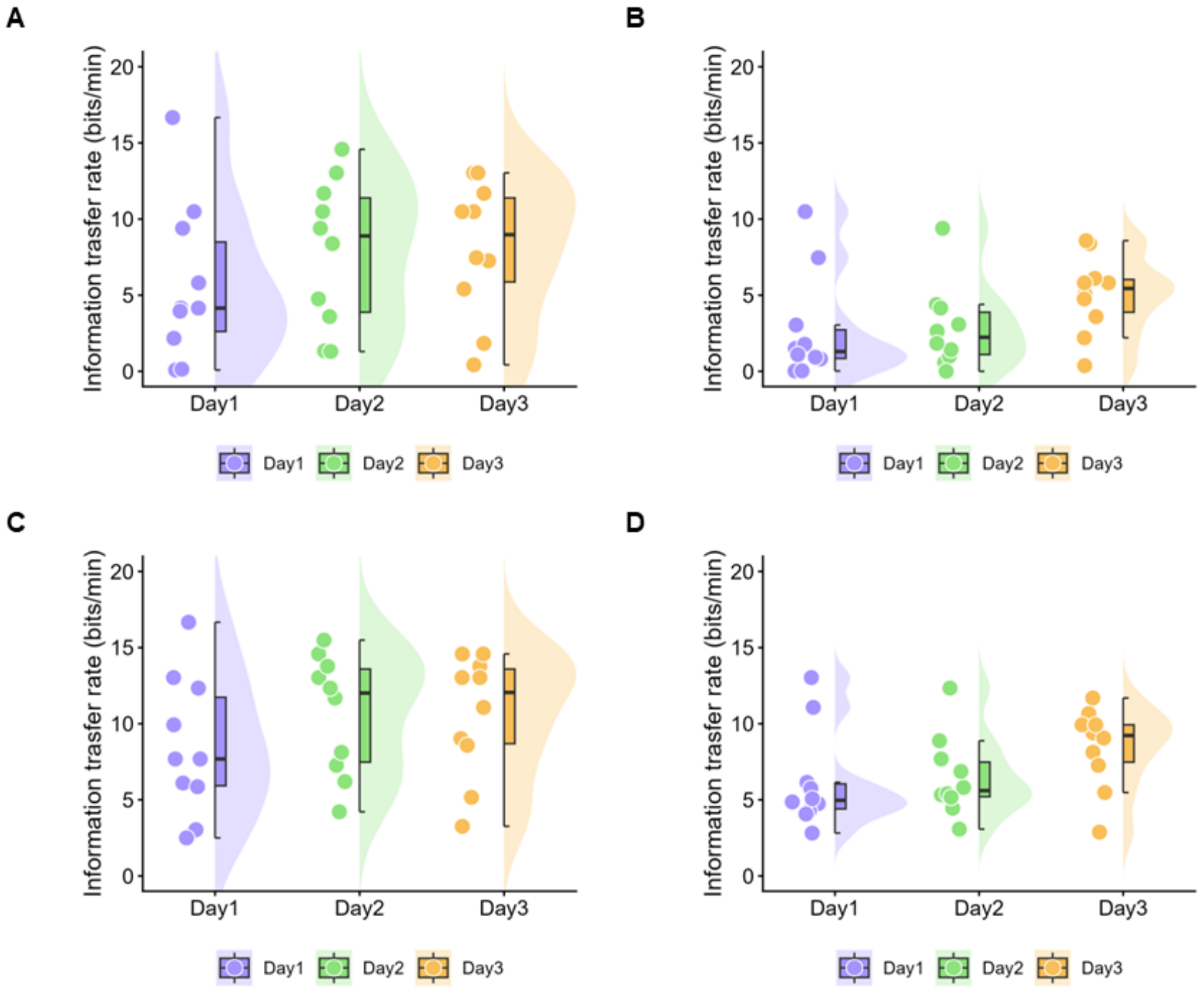

Figure S10. Distributions of information transfer rate (ITR) for the tactile-encoded brain–computer interface (BCI) over three training days. Violin plots show participant-level ITR with embedded boxplots. ITR was calculated based on online success rate in (A) single-task and (B) dual-task conditions, and based on accuracy in (C) single-task and (D) dual-task conditions.

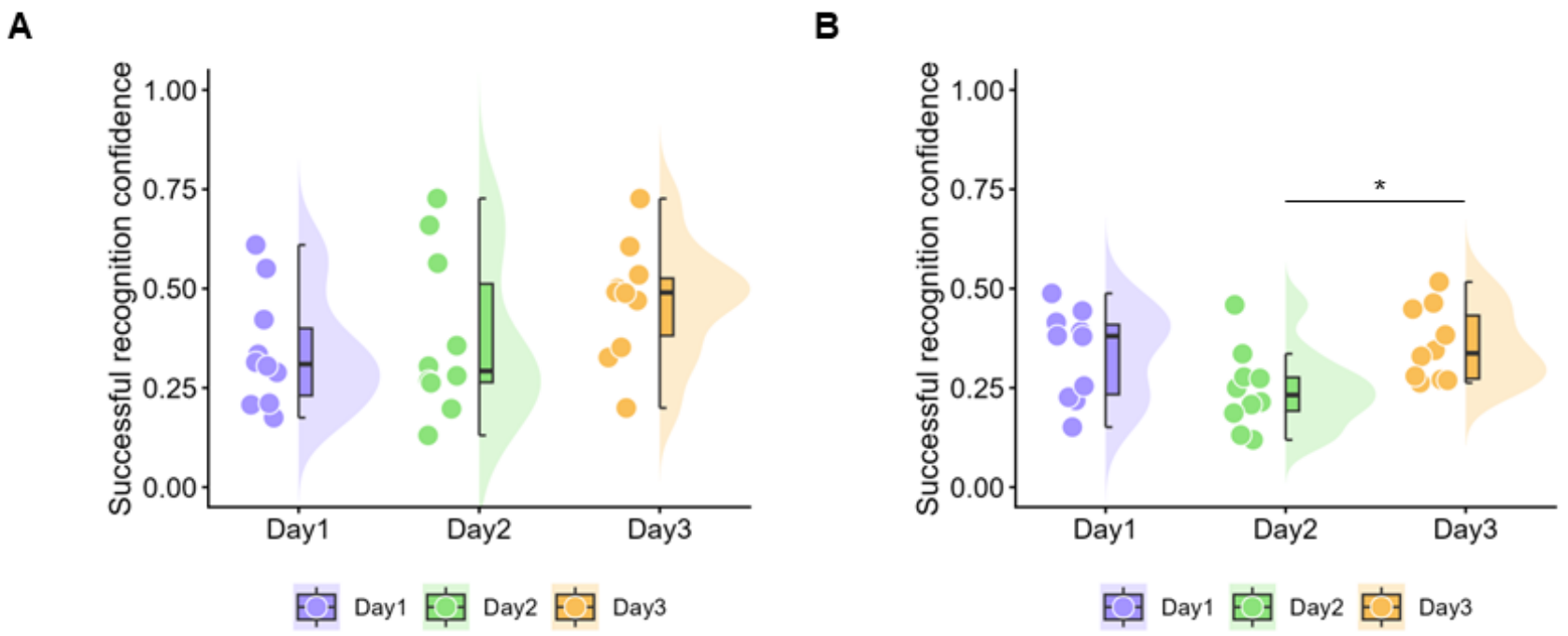

Figure S11. Distributions of successful recognition confidence for the tactile-encoded brain–computer interface (BCI) over three training days. Violin plots show participant-level recognition confidence with embedded boxplots. (A) single-task and (B) dual-task conditions. (* if $p < 0.05$; one-way repeated-measures ANOVA).

The Reviewer's Comment #3

Having worked on robotic applications, I would also like the authors to explicitly address if there were any safety constraints associated with the robotic motion for safety purposes: it sounds like if there were, they didn't influence the results, but it would be good to be explicit about this (possibly in the supplementary information).

Response:

We thank the reviewer for raising this point. For safety purposes, the system operated under predefined motion constraints, including limits on velocity and workspace boundaries, and BCI outputs triggered only validated motion primitives rather than unrestricted control. Commands were not artificially constrained to occur in a fixed sequence, and participants could in principle trigger any command at any time. However, to prevent unsafe or nonsensical transitions, the task logic included safety interlocks such that if an out-of-sequence command was triggered, the workflow was terminated rather than executing an invalid action. These safeguards were applied consistently across sessions and did not affect the decoding algorithm or the reported performance metrics. Moreover, an emergency button was provided for both participants and experimenters to ensure safety. A description of these safety measures has been added to the Supplementary Information.

"**Supplementary Materials: Safety constraints for robotic motion**

For safety purposes, the robotic arms operated under predefined motion constraints during the functional demonstration tasks. These constraints included limits on joint velocity, acceleration, and workspace boundaries enforced by the robot's built-in control framework. In addition, commands issued by the BCI triggered only predefined motion primitives rather than continuous free-space control, ensuring that all executed movements followed validated trajectories. An emergency button was provided for both participants and experimenters to ensure safety. Importantly, these safety constraints were applied uniformly across all experimental runs and did not alter the decoding process or the evaluation of BCI performance metrics."

The Reviewer's Comment #4

Finally, a brief discussion on how tactile adaptation might affect long-duration, real-world use outside the lab.

Response:

Thank you for raising this important point. We have added a brief discussion addressing the potential tactile adaptation during prolonged use and possible mitigation strategies in real-world applications.

"In addition to decoding speed and computational efficiency, tactile adaptation to repeated vibrotactile stimulation may also affect real-world applications. Repeated exposure may reduce the salience of the stimulus and thereby lessen the attention allocated to it, which could contribute to a weaker P300 response over time. Although this effect was not systematically evaluated in the present study, it may influence BCI performance during extended real-world operation. Future work should investigate how prolonged tactile stimulation influences BCI performance and user experience during extended use."

The Reviewer's Comment #5

In fig. 1, the first vignette on the bottom right is blank. I'd have all 4 commands here, i.e., add the yellow bracelet version of this.

Response:

Thank you very much for your valuable suggestion. We realize that the previous version of Figure 1 could be misleading for the readers. In the revised version, we have added a new vignette showing the yellow bracelet version. Additionally, we maintain the first blank vignette to demonstrate that when no vibrator is attended to, no P300 is generated, and consequently, the robotic system remains idle. This serves as a control condition, visually representing the "no-command" state.

Additionally, we have realised that the different coloured P300 waveforms presented in the lower panels of Fig. 1 may erroneously lead readers to a misunderstanding, specifically, that different vibrators elicit distinct P300 patterns, and that the algorithm identifies commands based on these differences. This is not the case, and we appreciate the opportunity to clarify. Actually, we do not distinguish which vibrator elicited a specific P300. All P300 responses are treated as the target class, while all non-P300 responses are treated as the other class. The identification of the attended vibrator relies on the time-locked nature of the P300 potential. That is, a P300 is expected to appear approximately 300 ms after the onset of the target vibration stimulus. The classifier detects the presence of P300 across vibrators, and the system determines the intended command based on which vibrator's onset time corresponds to the detected P300. Thus, the classifier does not differentiate the P300 waveforms from different vibrators, it only detects the occurrence of a P300 event. We have further clarified the above by slightly editing the text as follows:

"The expansion of DoFs is attributable to the tactile-encoded P300 paradigm, which does not require distinguishing P300 patterns across vibrators. Extending to four DoFs still remains binary, not four-class, classification. The classifier simply detects the presence of a P300, and the system identifies the intended command by the corresponding vibration onset time. This greatly reduces decoding complexity while increasing control dimensionality.

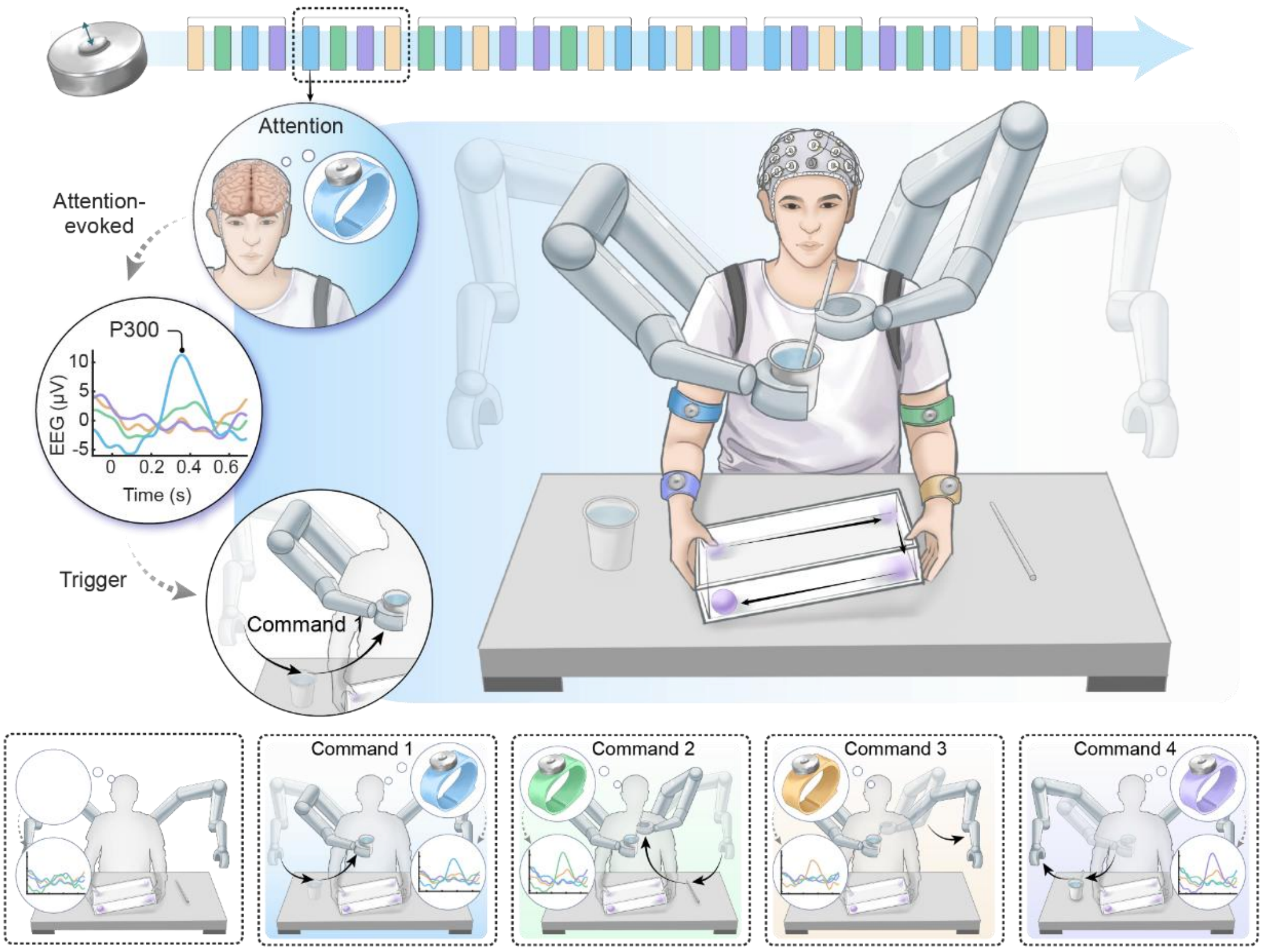

Fig. 1. Experimental setup for tactile-encoded BCI control of supernumerary robotic arms. Four tactile vibrators were attached bilaterally to the upper and lower arms, each delivering 200-ms bursts of vibration with 200-ms interstimulus intervals in a pseudorandom order. Each vibrator corresponded to one of four selectable control states, with symmetric placement to avoid proprioceptive bias. Attended stimuli evoked P300 responses in the EEG, which were decoded online to trigger corresponding robotic commands. A transparent tray was used for the ball-balancing task in the dual-task condition."

Reviewer #2

The Reviewer's Comment #1
I co-reviewed this manuscript with one of the reviewers who provided the listed reports. This is part of the Nature Communications initiative to facilitate training in peer review and to provide appropriate recognition for Early Career Researchers who co-review manuscripts.

Response:
We sincerely appreciate your time and effort dedicated to reviewing our manuscript. We greatly appreciate the constructive feedback and the insightful comments, which have helped us improve our work. We have carefully considered all the suggestions and have incorporated the corresponding changes into the manuscript. All revisions are highlighted within the manuscript for your convenience.

Reviewer #3

The Reviewer's Comment #1
The Introduction part can be further expanded to include state-of-the-art in BCI for augmentation by extension. Incorporating several key recent studies would help readers more clearly understand the recent advances, current research level and the specific challenge this study aims to address.

Response:
Thank you very much for this comment. To the best of our knowledge, there are currently no recent BCI studies that clearly achieve augmentation by extension. A key limitation we have identified is that, although several studies demonstrated the BCI control of supernumerary effectors, this control is often achieved at the expense of functionality loss in other areas. As such, these approaches are better characterised as augmentation by transfer rather than extension. For example, Xie et al. (2024) employed classification of steady-state visually evoked potentials (SSVEPs) to infer user intent and trigger pre-programmed commands for a robotic third arm. While this approach enables control via motor-unrelated neural pathways, it relies intensely on the visual system, thereby imposing a competing demand on visual resources. We argue that preservation of visual function is critical, particularly for enabling bimanual tasks that are supplemented by supernumerary effector control. To clarify these challenges for the reader, we have incorporated a summary of the study by Xie et al., along with additional examples (studies Leeb et al. (2013) and Cheung et al. 2012)). These works highlight the fundamental constraints of augmentation-by-transfer paradigms and motivate the need for new approaches, such as ours, that move toward increasing DoF with no detriment to body function.

> "Sensorimotor rhythms (SMRs) from EEG have been widely used for user command recognition in individuals with severe motor disabilities *(14, 15)*. In paradigms involving ongoing movements, SMR activity is typically modulated using motor imagery (MI) of a task-irrelevant body part *(16, 17)*. Similarly, Xie et al. employed classification of steady-state visually evoked potentials (SSVEPs) from occipital EEG channels to infer user intent and trigger pre-programmed commands for a robotic third arm *(18)*. While compelling, these approaches highlight the central challenge of augmentation: the redistribution of cognitive resources or detriment of natural motor function. More closely aligned with the augmentation by extension, Penaloza & Nishio demonstrated a SMR-based BCI, where able-bodied participants activated a robotic arm through grasping MI while performing a bimanual ball-balancing task concurrently *(2)*. However, their system functioned as a single discrete brain switch, and the extension to more DoFs faces significant challenges."

References:

2. C. I. Penaloza, S. Nishio, BMI control of a third arm for multitasking. Sci. Robot. 3, eaat1228 (2018).
14. J. R. Wolpaw, D. J. McFarland, G. W. Neat, C. A. Forneris, An EEG-based brain-computer

interface for cursor control. Electroencephalogr. Clin. Neurophysiol. 78, 252-259 (1991).
15. A. Biasiucci, R. Leeb, I. Iturrate, S. Perdikis, A. Al-Khodairy, T. Corbet, A. Schnider, T. Schmidlin, H. Zhang, M. Bassolino, D. Viceic, P. Vuadens, A. G. Guggisberg, J. D. R. Millán, Brain-actuated functional electrical stimulation elicits lasting arm motor recovery after stroke. Nat. Commun. 9, 2421 (2018).
16. R. Leeb, M. Lancelle, V. Kaiser, D. W. Fellner, G. Pfurtscheller, Thinking Penguin: Multimodal Brain-Computer Interface Control of a VR Game. IEEE Trans. Comput. Intell. AI Games 5, 117-128 (2013).
17. W. Cheung, D. Sarma, R. Scherer, R. P. N. Rao, Ieee, in 34th Annual International Conference of the IEEE Engineering-in-Medicine-and-Biology-Society (EMBS). (IEEE, San Diego, CA, 2012), pp. 6715-6718.
18. P. Xie, Y. Men, J. Zhen, X. Shao, J. Zhao, X. Chen, The supernumerary robotic limbs of brain-computer interface based on asynchronous steady-state visual evoked potential. J. Biomed. Eng. 41, 664-672 (2024).

The Reviewer's Comment #2

In Figure 1, the P300 component is labeled as "sensory-evoked." If I understand correctly, I think that "sensory-encoded" or "attention-evoked" may be more accurate. Sensory input should be also present for non-target stimuli but P300 is not evoked when the participant does not allocate attention to such stimuli. I therefore suggest that the authors reconsider which label is more appropriate.

Response:

We sincerely appreciate your insightful comment. We agree that "attention-evoked" is a more appropriate term. We have revised Fig. 1 accordingly.

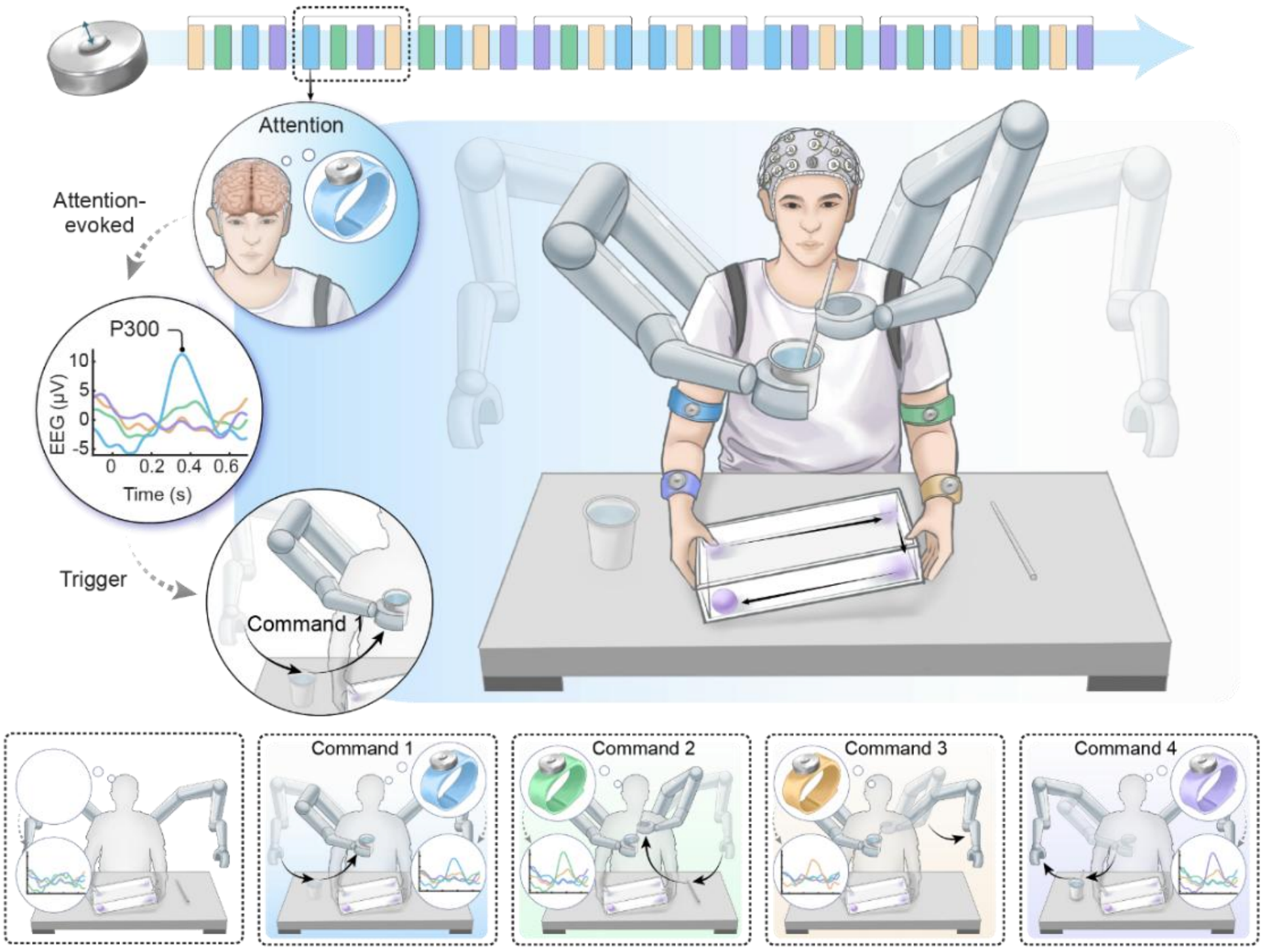

Fig. 1. Experimental setup for tactile-encoded BCI control of supernumerary robotic arms. Four tactile vibrators were attached bilaterally to the upper and lower arms, each delivering 200-ms bursts of vibration with 200-ms interstimulus intervals in a pseudorandom order. Each vibrator corresponded to one of four selectable control states, with symmetric placement to avoid proprioceptive bias. Attended stimuli evoked P300 responses in the EEG, which were decoded online to trigger corresponding robotic commands. A transparent tray was used for the ball-balancing task in the dual-task condition.

The Reviewer's Comment #3

The performance evaluation of the ball-balancing task serves as essential evidence supporting the concept of movement augmentation. However, the reasons for selecting the two evaluation metrics presented in Figure 4, as well as the detailed calculation methods, are not clear enough. I recommend adding a brief explanation to clarify this part.

Response:

We thank the reviewer for this suggestion. The two metrics used to evaluate the ball-balancing task addressed the following task characteristics: (i) time per successful rotation, and (ii) time per rotation across all attempts, successful and not. These two metrics captured complementary aspects of natural motor performance during the task. Specifically, the first metric reflects execution efficiency conditional on successful task completion, while the second metric includes unsuccessful attempts and therefore captures the overall temporal stability of performance. We hope these clarifications can improve the clarity of the performance evaluation. To clarify their rationale and calculation, we have added a brief explanation in the manuscript and provided a detailed description in the Supplementary Materials, as follows:

"We evaluated two complementary performance metrics: (i) averaged effective rotation time, calculated as the number of successful rotations divided by total task time, and (ii) averaged gross rotation time, calculated as the total number of rotation attempts, including unsuccessful ones, divided by total task time. Detailed definitions and rationale for these metrics are provided in Supplementary Materials. Participants were instructed to perform the task at a steady and controlled pace rather than maximise speed, thereby minimising speed-accuracy trade-offs.

Supplementary Materials: Quantifying ball-balancing performance

Natural movement performance during the ball-balancing task was quantified using two complementary temporal metrics. The first metric, time per successful rotation, was calculated as the total task duration divided by the number of successfully completed rotations. A rotation was considered successful when the ball passed sequentially through all four corners of the tray. This metric reflects execution efficiency conditional on correct task completion. The second metric, time per rotation across all attempts, was calculated as the total task duration divided by the total number of rotation attempts, including

unsuccessful rotations. Because unsuccessful rotations reduced the effective completion rate, this measure captures the overall temporal stability of task performance and implicitly incorporates the impact of errors. Participants were instructed to perform the task at a steady and controlled pace rather than as quickly as possible. Under these conditions, the two metrics provide a complementary measure of coordination efficiency and stability during concurrent BCI use."

The Reviewer's Comment #4

I notice that the Bayes factor of the time per successful rotation on Day 1 is over commonly-used standard . While the null effects can be supported following training, the results on Day 1 still need appropriate explanation.

Response:

Thank you for your thoughtful observation. It is true that while the ANOVA indicated no significant differences across conditions, the Bayes factor for this specific measure on Day 1 did not reach the conventional threshold to strongly support the null hypothesis. We agree that this result warrants an explanation. It is important to note that for all participants, Day 1 represented their first exposure to both the balancing task and the tactile-encoded P300 control. Although the experimental procedure included a practice phase, we suspect that the relatively weaker evidence for the null on Day 1 reflects an initial learning or adaptation effect. Participants were likely still familiarizing themselves with the novel multitask paradigm, which may have introduced slight variability in their initial performance. Crucially, this effect appears to be transient. As shown on day 2 and day 3, the Bayes factor analysis strongly supports the conclusion that the tactile-encoded BCI does not interfere with natural human movement. This pattern, initial variability followed by stabilization, further reinforces the robustness of our main finding. We have now added a brief explanation in the revised manuscript to provide readers with a more complete interpretation of the Day 1 results.

"The BF_{10} for day1 showed inconclusive evidence for the null hypothesis, which could be attributed to participants' first exposure to the novel ball-balancing and tactile-encoded P300 control tasks. However, following training, the results of day2 and day3 supported the observation that use of the tactile-encoded BCI did not affect the efficiency or temporal stability of natural bimanual coordination."

The Reviewer's Comment #5

I notice that the decoding of P300 relied on commonly-used algorithms. The authors could consider adding an appropriate discussion on advances in P300 recognition algorithms. This would provide valuable references for researchers who are inspired by this work and wish to further investigate this novel paradigm. For motor imagery-based BCIs, it has been reported that a certain proportion of subjects are BCI illiteracy. For the novel BCI paradigm proposed in this study, have similar phenomena been observed in the experiments? I suggest adding the discussion on individual performance differences.

Response:

We thank the reviewer for this suggestion. In the revised manuscript, we have refined the discussion section to more clearly highlight representative developments in P300 recognition, including single-trial decoding, deep-learning approaches, calibration-reduction methods, and wearable implementations, and to better explain their relevance for future improvements of the present tactile-encoded paradigm.

We also thank the reviewer for the insightful suggestion on exploring BCI illiteracy regarding our proposed novel BCI paradigm. In response, we used K-means clustering to identify the subjects classified as the poorest performers. Specifically, to identify distinct performance subgroups, we performed K-means clustering on the three-dimensional data (Day 1, Day 2, and Day 3 success rates) for all 10 participants. Data were z-score normalized prior to clustering to ensure equal weighting across days. As shown in Figure R1, the optimal number of clusters (k = 3) was determined using the elbow method, which revealed a clear inflection point at three clusters. Clustering was repeated 50 times with different initializations to avoid local optima, and the solution with the lowest within-cluster sum of squares was selected. The stability of the clustering solution was confirmed by a perfect adjusted Rand index (ARI = 1.00) across multiple runs.

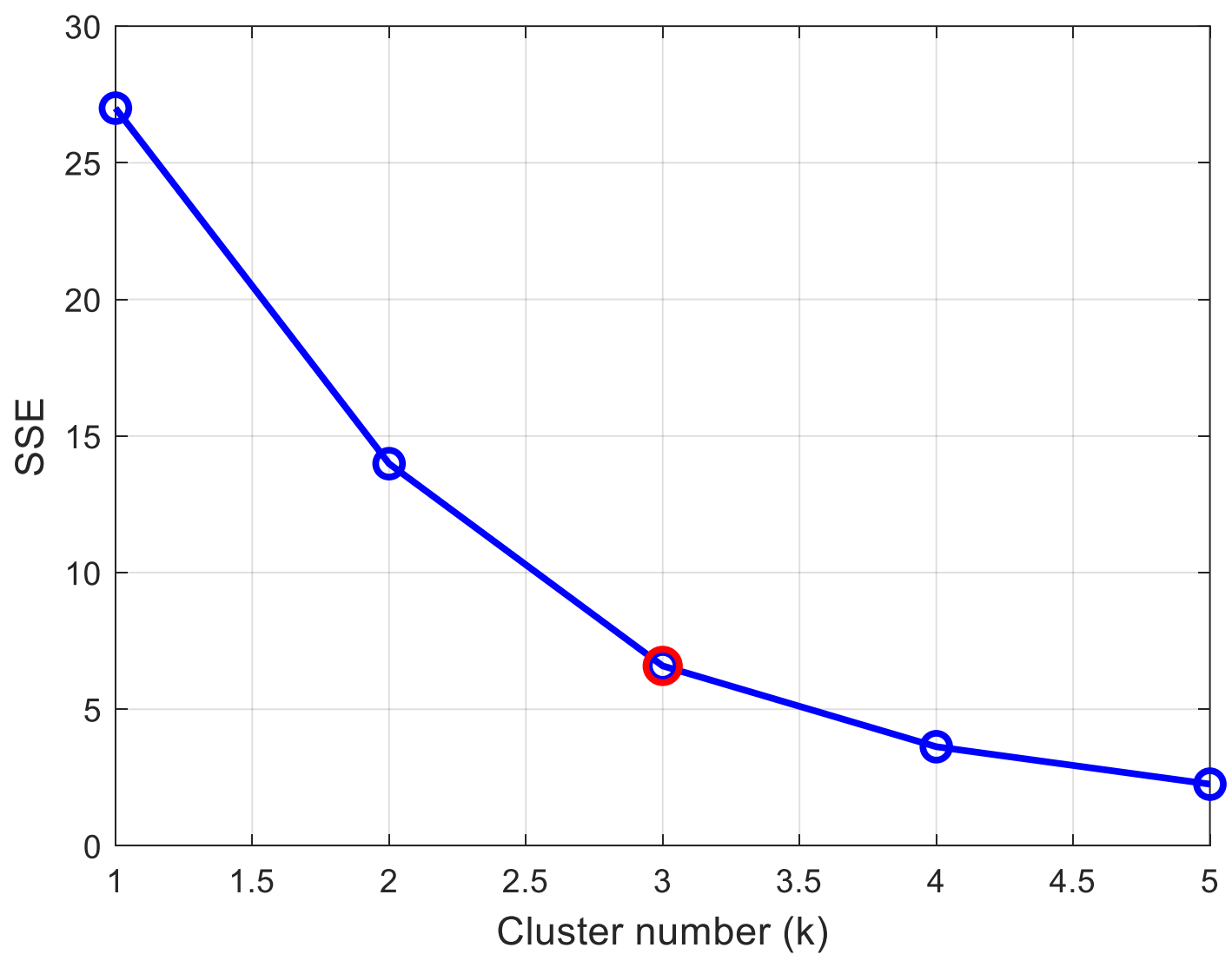

Figure R1. Elbow method to determine the optimal number of clusters.

The K-means clustering analysis revealed three distinct performance profiles, as summarized in Table R1 and Figure S9. High Performers (Cluster 3): Subjects 2, 5, and 6 exhibited consistently high success rates across all three days (overall mean = 76.4%). Notably, this group showed minimal improvement over time (2.1%), suggesting they had reached a performance ceiling from the outset. Intermediate Learners (Cluster 1): Most participants (n = 6) fell into this cluster, characterized by moderate initial performance (Day 1: 45.3%) and substantial improvement across sessions (24.5% improvement), eventually reaching levels comparable to the high performers by Day 3 (69.80%). Low Performers (Cluster 2): Subject 9 was identified as an outlier with persistently low performance (overall mean = 31.3%). This subject's improvement trajectory (12.50% over three days) was substantially shallower than that of the intermediate performers (24.48%), despite starting

from a much lower baseline.

As noted by the reviewer, BCI illiteracy has been reported in motor imagery (MI)-based BCIs. In a MI BCI with binary choice, a correct response rate (accuracy) of 70% is necessary for communication (Perelmouter and Birbaumer, 2000). For four-class classification, the chance accuracy level is 25%. The threshold for defining BCI illiteracy in four-class BCIs is less standardized than in two-class, but a commonly adopted operational threshold is chance level (25% for four classes) + 2 standard deviations above chance. Therefore, approximately, minimally acceptable performance for four-class BCI tasks as ≥ 45–50% accuracy. Users falling consistently below 40% are usually classified as BCI illiterate. In alignment with this, our clustering analysis revealed a similar phenomenon within the proposed tactile-encoded BCI paradigm, a characteristic exhibited specifically by Subject 9.

Table R1. Cluster characteristics based on three-day success rates (Mean ± SD)

Cluster No.	Subjects	Success rates (%)				
		Day 1	Day 2	Day 3	Mean	Improvement (Day 3-Day1)
1	1,3,4,7,8,10	45.3±9.2	53.1±10.1	69.8±7.3	56.1	24.5
2	9	28.1	25.0	40.63	31.3	12.5
3	2,5,6	77.1±11.0	72.9±10.0	79.2±7.9	76.4	2.1

The following has been added to the revised manuscript:

"Further improvements in responsiveness will depend on the advances in single-trial P300 decoding, although such approaches must balance decoding accuracy, computational cost, and real-time feasibility. De Venuto et al. developed an embedded single-trial P300 decoder that increased the BCI responsiveness, while the validation was limited to only speller data *(34)*. Leoni et al. introduced an explainable deep learning pipeline that improved single-trial decoding accuracy with increased computational cost *(35)*. Du et al. reported that fusing multi-subject CNN features boosted the accuracy of single-trial P300 decoding to above 80%, despite requiring large datasets and training overhead *(36)*. Kim et al. demonstrated a calibration-free "plug-and-play" single-trial BCI using pre-trained spatial filters, showing promising real-time use *(37)*. In parallel, Eidel et al. observed that tactile P300 could be recorded using ear-mounted electrodes, highlighting the practical trade-off between improved wearability and reduced signal quality *(38)*. Collectively, these developments suggest that improving single-trial decoding while maintaining high computational efficiency will be important for enhancing the practical performance of our proposed tactile-encoded BCIs.

References:

34. D. De Venuto, G. Mezzina, A single-trial P300 detector based on symbolized EEG and autoencoded-(1D)CNN to improve ITR performance in BCIs. Sensors 21, 3961 (2021).
35. J. Leoni, S. C. Strada, M. Tanelli, A. Brusa, A. M. Proverbio, Single-trial stimuli

classification from detected P300 for augmented brain-computer interface: A deep learning approach. Mach. Learn. Appl. 9, 100393 (2022).
36. P. Du, P. H. Li, L. L. Cheng, X. Q. Li, J. X. Su, Single-trial P300 classification algorithm based on centralized multi-person data fusion CNN. Front. Neurosci. 17, 1132290 (2023).
37. J. Kim, S. P. Kim, A plug-and-play P300-based BCI with zero-training application. IEEE Trans. Neural Syst. Rehabil. Eng. 33, 3443-3454 (2025).
38. M. Eidel, M. Pfeiffer, P. Ziebell, A. Kuebler, Recording the tactile P300 with the cEEGrid for potential use in a brain-computer interface. Front. Hum. Neurosci. 18, 1371631 (2024).

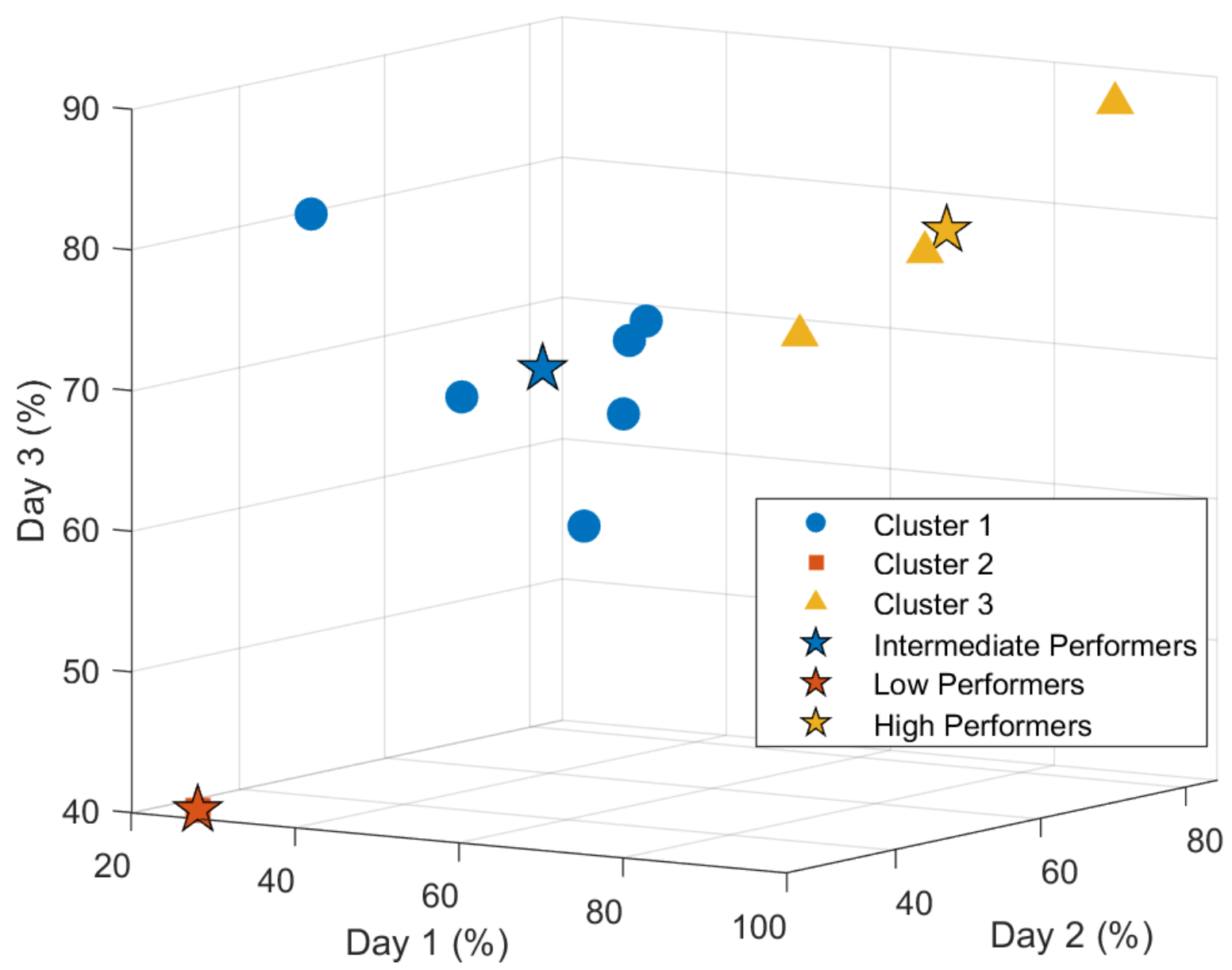

Figure S9. Individual difference analysis using K-means clustering based on success rates of dual-task runs across days 1-3. Three-dimensional scatter plot shows the distribution of 10 participants' success rates on Days 1, 2, and 3. Data points are color-coded by cluster membership (blue: Cluster 1, Intermediate Performer; red: Cluster 2, Low Performer; yellow: Cluster 3, High Performers). One low performer can be regarded as BCI illiteracy of the tactile-encoded BCI."

The Reviewer's Comment #6
The online performance demonstrated significant improvement after training. Notably, a significant performance gain was sustained from Day 2 to Day 3, which may indicate that the dual-task performance has not yet reached its ceiling. It is recommended that the authors include discussion regarding this potential performance ceiling.

Response:
We thank the reviewer for this insightful suggestion. The continued improvement observed in the dual-task condition between Day 2 and Day 3 indeed suggests that performance may not yet have reached its ceiling under concurrent task demands. By contrast, no

significant learning effect was observed in the single-task condition, which may indicate that the single-task performance approached the effective performance ceiling for this paradigm. We have added a brief discussion in the revised manuscript to clarify this interpretation and to note that further training could potentially lead to additional improvements in the dual-task performance.

"We thus hypothesise that through training, participants learned to effectively allocate attention during the dual BCI tasks, leading to more stabilised P300 latency. However, it was not possible to infer a learning effect in BCI control solely from changes in the CV of the P300 latency, especially because other metrics of performance, such as information transfer rate (ITR), did not fully correlate with CV. Interestingly, while performance improvements were observed across sessions in the dual-task condition, no significant learning effect was detected in the single-task condition. This pattern may indicate that performance in the single-task condition approached the effective ceiling for this paradigm, whereas the additional attentional and coordination demands in the dual-task condition likely left greater room for improvement. The continuous performance improvement observed on day 2 and day 3 suggests that dual-task performance may not have reached its ceiling. Future studies with longer training time could help determine whether further improvements can be achieved under dual-task conditions."

The Reviewer's Comment #7

Please define the abbreviation before its first use, such as "SNR". Please ensure all abbreviations used in the manuscript follow this convention. Please standardize the spelling throughout the manuscript. For instance, both the British English variant "artefacts" and the American English variant "artifacts" are used in the manuscript to denote EEG noise.

Response:

We thank the reviewer for this suggestion. The manuscript has been carefully reviewed to ensure that all abbreviations are defined prior to their first use. In addition, British English spelling has now been applied consistently throughout the manuscript in accordance with Nature Portfolio conventions.

"Artifacts" to "artefacts", "Minimize" to "minimise". Etc.

Reviewer #4

The Reviewer's Comment #1
Choice of sensory modality. It is critical to clarify why tactile modality is chosen over auditory modality. The suggested movement augmentation paradigm could also be achieved by auditory P300-based BCIs. Perhaps, auditory stimuli can offer a more independent control channel than tactile stimuli because of innate coupling between tactile perception and arm movement. But tactile modality may have other advantages, too. So, please provide rationale to choose tactile modality in this study.

Response:
We thank the reviewer for this comment. Indeed, tactile and auditory P300 spellers are both feasible options for communicating commands through a BCI channel, but tactile systems tend to produce slightly higher accuracies, and higher information transfer rates. Critically for movement augmentation where there is a concurrent movement task, tactile systems have lower subjective workload than auditory systems in matched paradigms, especially after some training (Ziebell et al., 2020). This motivated our choice. There may be use cases in which auditory stimuli are preferable, for example when somatosensation is impaired, however, in the spirit of human augmentation, we see our system primarily as an extension of the DoF of able-bodied users, and therefore aim to preserve as many sensory resources as possible in order to respect the principles of augmentation by extension. We have added the following sentence to the Introduction of the revised manuscript to explain this distinction:

"Auditory implementations have also been proposed, but they typically achieve lower accuracies and, critically, for movement augmentation, are associated with increased subjective workload *(23)*."

References:
23. P. Ziebell, J. Stuempfig, M. Eidel, S. C. Kleih, A. Kuebler, M. E. Latoschik, S. Halder, Stimulus modality influences session-to-session transfer of training effects in auditory and tactile streaming-based P300 brain-computer interfaces. *Sci. Rep.* 10 (2020).

The Reviewer's Comment #2
Sample size. The number of participants (N=10) in the current study appears to be too small to validate statistical significance of the results. Please assess the number of participants (sample size) to achieve meaningful statistical power using appropriate measures (e.g., G*Power) and increase the sample size up to a desired figure by additionally recruiting participants.

Response:
We thank the reviewer for raising the important issue on sample size. To address this

concern, we conducted a post-hoc power analysis using G*Power (Faul et al., 2007) focused on our primary outcome measure: the online success rate for dual-task runs across days 1–3. The analysis revealed a large effect size (Cohen's $f = 1.043$, $\eta_p^2 = 0.521$), resulting in a statistical power exceeding 0.99 with our sample of 10 participants. This result demonstrates that our sample was sufficiently large to detect the significant effects reported in the manuscript. We have now included this justification in the revised Supplementary Materials.

"G*Power *(48)* was used for a post-hoc power analysis.

Supplementary Materials: Statistical power analysis

To evaluate whether the sample size (N = 10) was adequate to support the main findings, we performed a post-hoc power analysis using G*Power. The analysis was conducted on the primary outcome measure: the online success rate for dual-task runs across days 1–3. The analysis revealed a large effect size (Cohen's $f = 1.043$, $\eta_p^2 = 0.521$), resulting in a statistical power exceeding 0.99 with the sample of 10 participants. This result demonstrates that the sample size is sufficiently large to detect the significant effects reported in this study."

References:

48. F. Faul, E. Erdfelder, A. G. Lang, A. Buchner, G*Power 3: A flexible statistical power analysis program for the social, behavioral, and biomedical sciences. Behav. Res. Methods 39, 175-191 (2007).

The Reviewer's Comment #3

Sense of embodiment. One of the critical points in movement augmentation is the emerging sense of embodiment. It would be important to find whether the sense of embodiment emerged or increased by training. Did the authors perform post-hoc surveys to assess subjective experiences of controlling supernumerary DoFs?

Response:

We thank the reviewer for highlighting the importance of embodiment and subjective experience in movement augmentation. The original experimental protocol did not include a pre-planned questionnaire specifically designed to assess embodiment or agency during the study sessions. Following the reviewer's suggestion, we conducted a retrospective post-hoc follow-up questionnaire to explore participants' subjective experience. Participants were asked to rate statements describing their experience during the experimental sessions using a 10-point Likert scale (1 = strongly disagree, 10 = strongly agree). Overall, the responses suggested a moderate sense of agency and perceived improvement in ease of use across sessions. However, this survey was conducted more than six months after completion of the experiment and was not part of the original experimental design. We therefore consider these results to be exploratory and potentially affected by recall bias. For this reason, we chose not to include these data in the revised

manuscript, in order to avoid overinterpreting retrospective subjective reports as formal experimental evidence. For transparency, we provide the questionnaire items and the aggregate results as follows:

1. I felt that I was in control of the supernumerary DoFs.

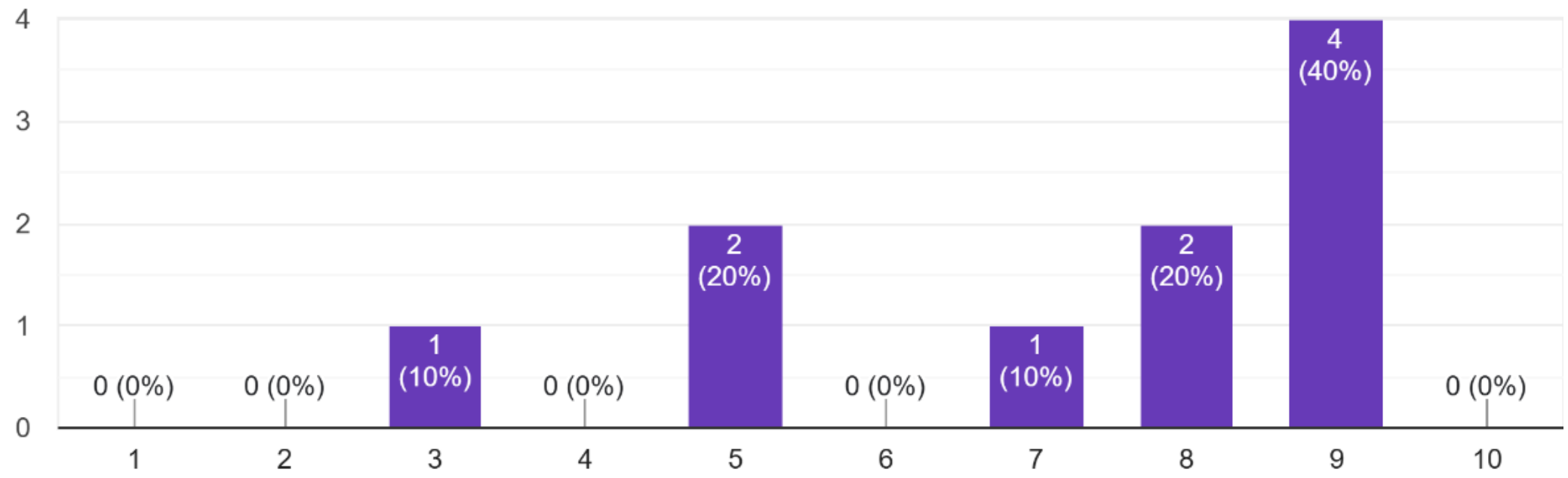

2. I could reliably predict what action would occur after I focused on a tactile cue.

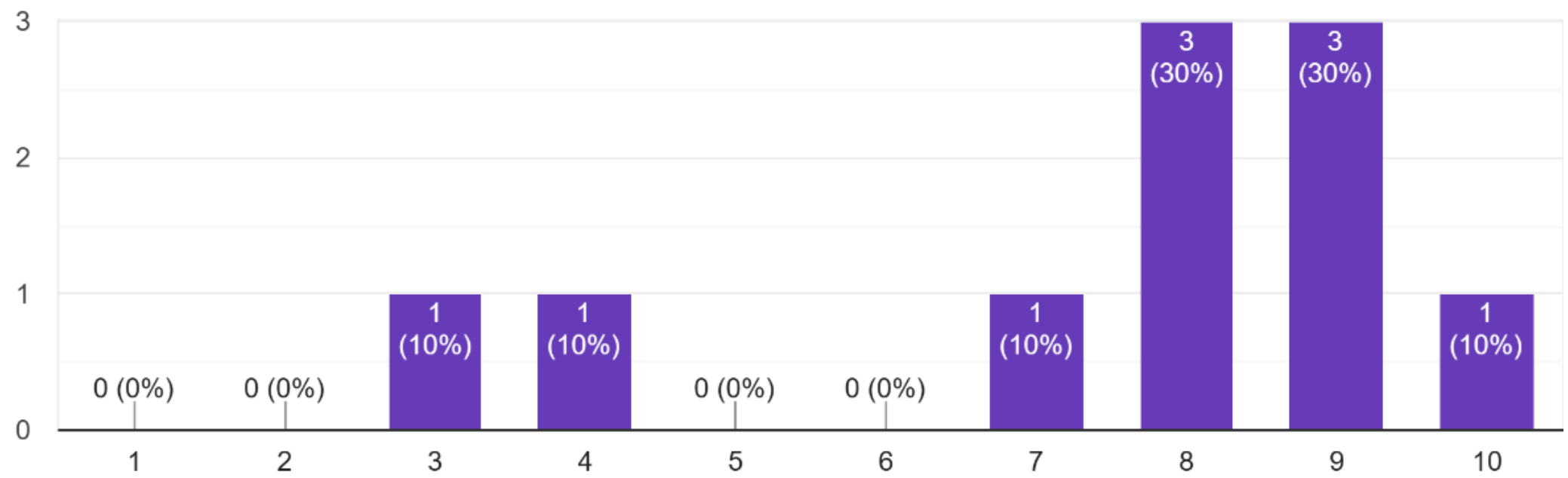

3. The system responded to my intention in a timely manner.

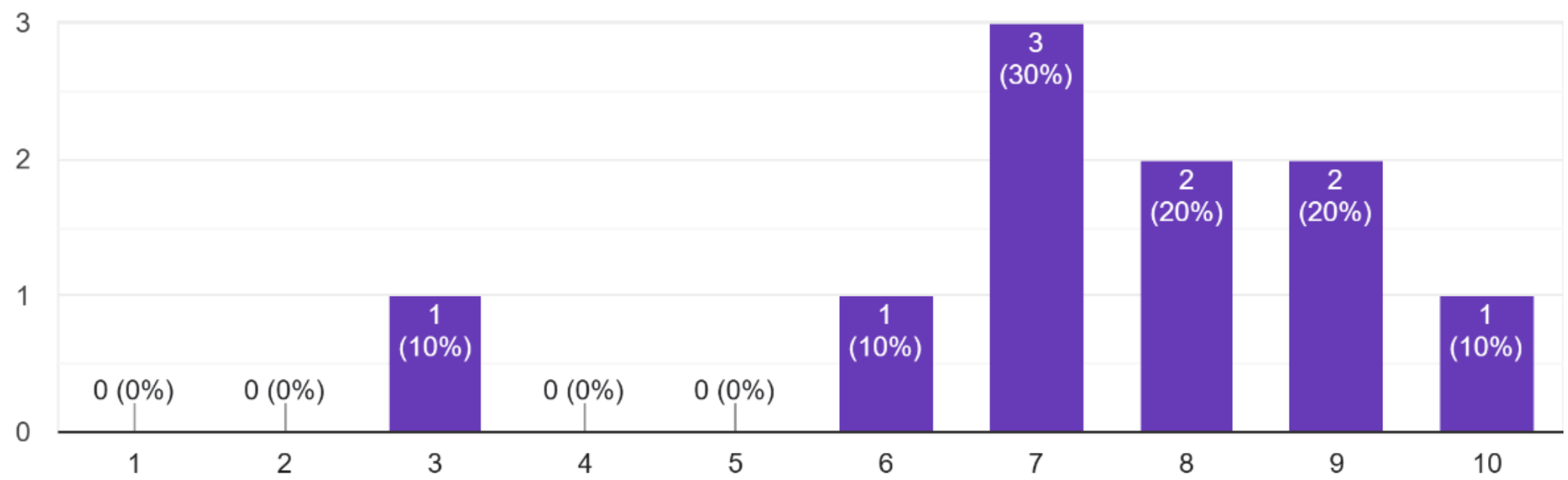

4. The system felt natural and intuitive to use.

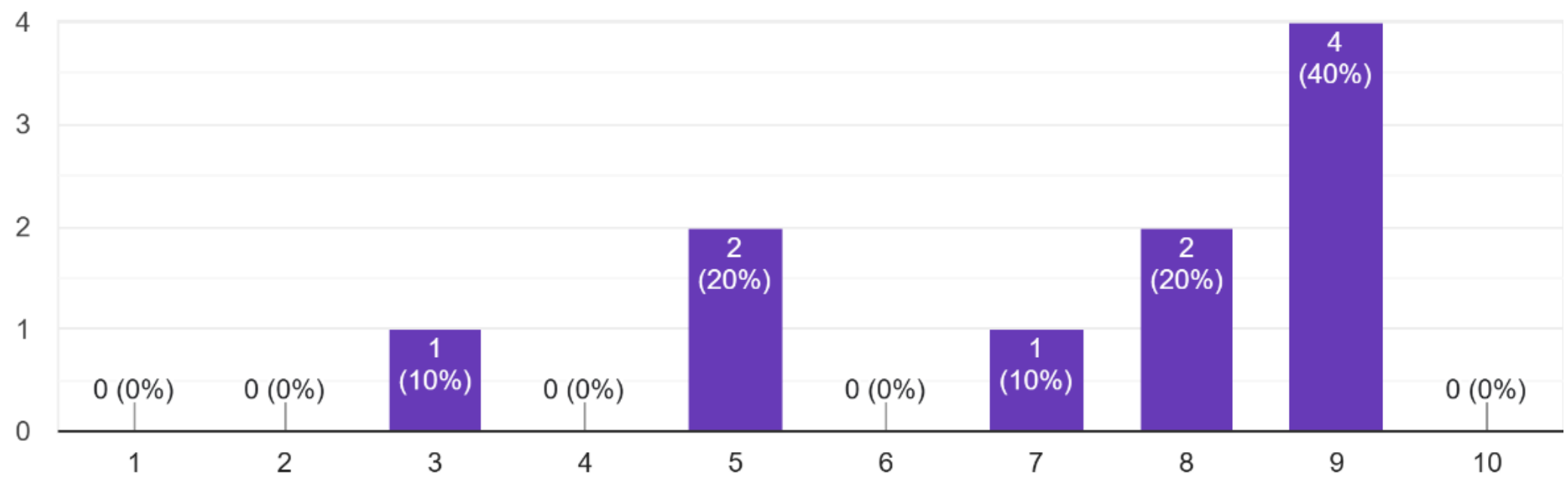

5. The tactile cues were easy to attend to while doing the motor task.

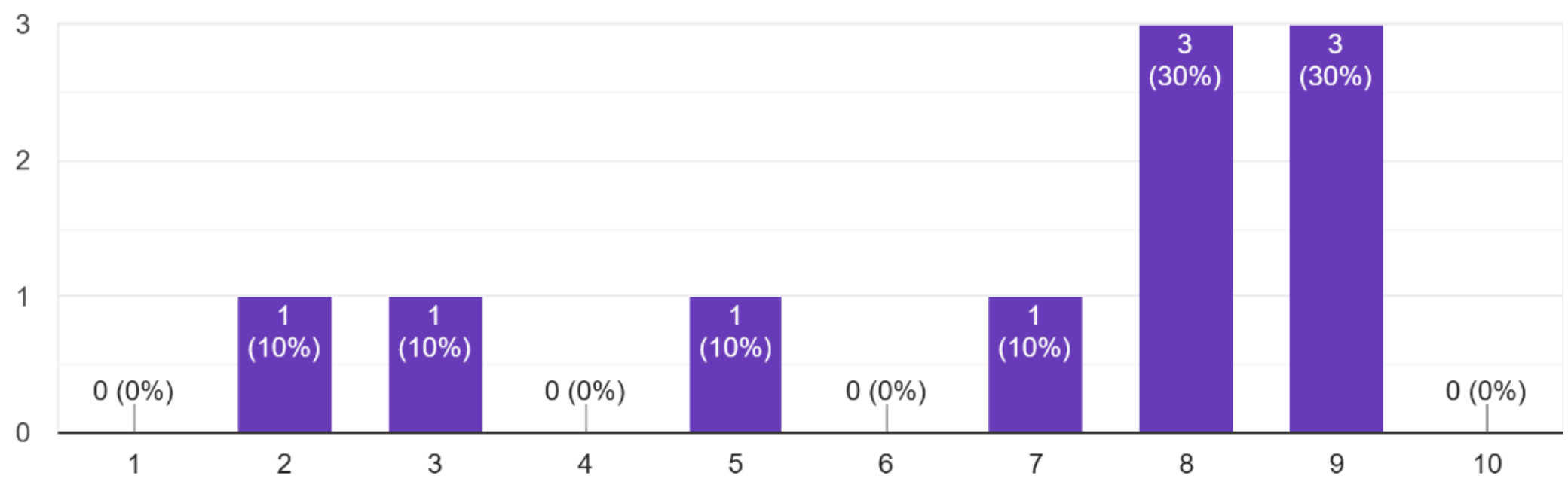

6. My ability to control the system improved across sessions/days.

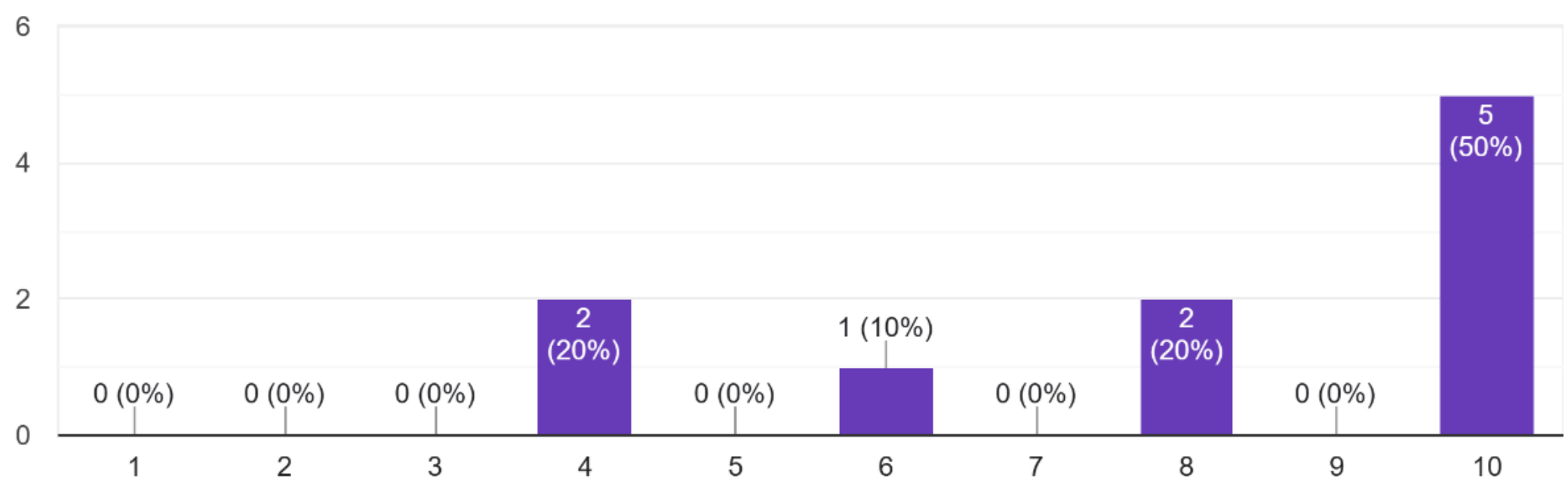

7. I experienced tactile adaptation/fatigue (e.g., vibration felt less noticeable over time).

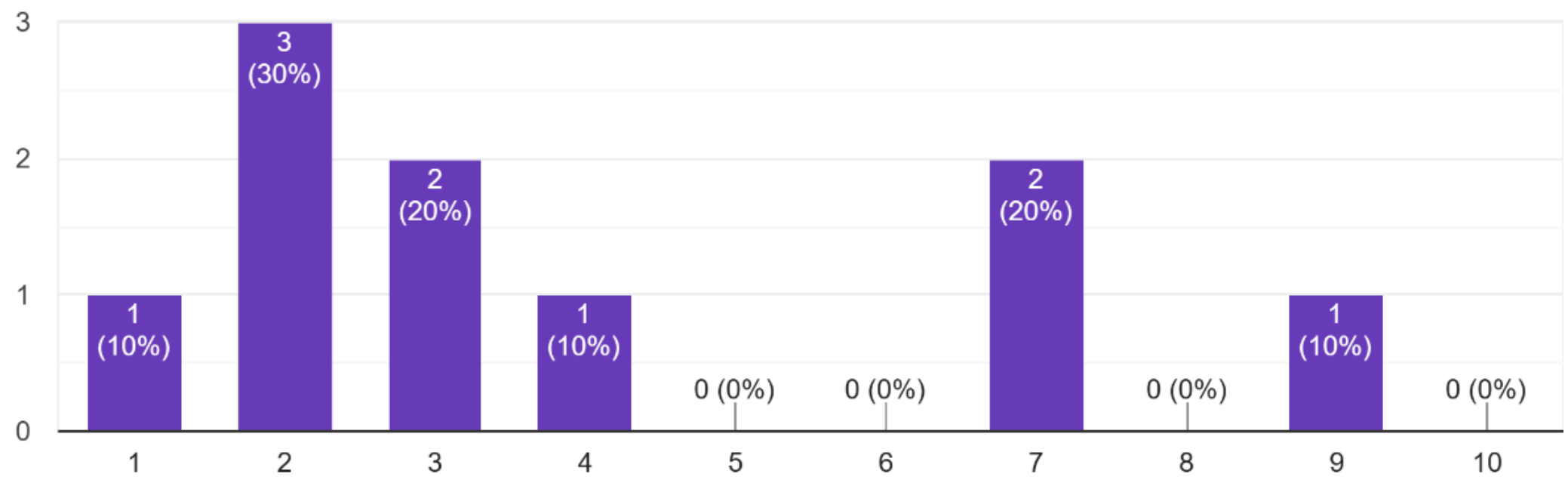

The Reviewer's Comment #4

Effect of training on neural responses. In Fig 2F, CV of P300 latency was similar between single- and dual tasks. Authors claimed that this evidences the training effect on neural responses. But no difference between the tasks could be due to increases in CV of single task. In that sense, the result of Fig 2F alone just partially supports the training effect on P300. Instead, the main effect of TIME (day) on CV of P300 latency followed by post-hoc comparisons (as in Fig 3B) should be done to fully support the authors' claim.

Response:

We sincerely appreciate your valuable suggestions. We have added further analysis to explore the main effect of TIME (day) on coefficient of variation (CV) of P300 latency using a repeated measures ANOVA with a Greenhouse-Geisser correction. The results are now shown in Figure S5. No significant changes in CV of P300 latency were observed over days for the single-task condition (Fig. S5A; $F_{1.59,14.29} = 0.22$, $p = 0.758$) (day1: 37.2±2.9 %; day2: 37.4±3.3 %; day3: 38.6±3.1 %). However, significant changes in CV of P300 latency were observed over days for the dual-task condition (Fig. S5B; $F_{1.75,15.76} = 7.11$, $p = 0.008$) (day1: 45.47±1.77 %; day2: 49.70±1.95 %; day3: 42.92±2.1 %). Post hoc analysis with a Bonferroni adjustment revealed that the CV significantly decreased from day 2 to day 3 [6.78 (95% CI, 0.63 to 12.94) %, $p = 0.031$], while not from day 1 to day 3 [2.55 (95% CI, -1.85 to 6.95) %, $p = 0.37$].

These results indicate that while no significant differences were observed across the three days for the single-task condition, the dual-task condition exhibited a significant reduction in trial-by-trial variability from day 2 to day 3. Although the difference between day 1 and day 3 did not reach statistical significance, the magnitude of change was comparatively larger than that observed in the single-task condition. Nevertheless, we realized that concluding a learning effect in BCI control based solely on changes in CV of P300 latency would be overly strong, as BCI learning effects are likely driven by multiple factors beyond this single metric. Accordingly, in the revised manuscript, we have modified the corresponding contexts to tone down the original conclusion. Figure S5 has also been

added to the Supplementary Materials. We thank you again for your insightful comments, which have helped us improve the rigor of our analysis and the clarity of our claims.

"However, it was not possible to infer a learning effect in BCI control solely from changes in the CV of the P300 latency, especially because other metrics of performance, such as ITR, did not fully correlate with CV.

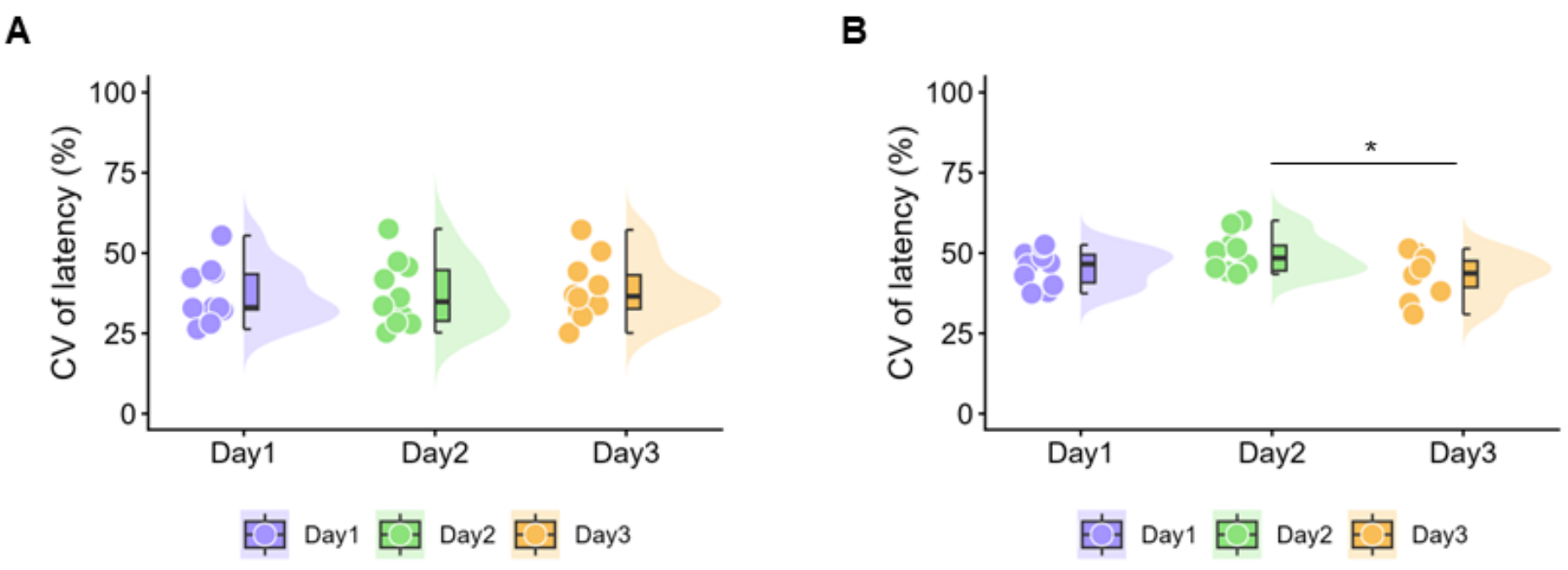

Figure S5. Coefficient of variation (CV) of P300 latency over three days of training. (A) single-task and **(B)** dual-task conditions. Violin plots show participant-level distributions with embedded boxplots. (* if $p < 0.05$; one-way repeated-measures ANOVA) "

The Reviewer's Comment #5

Neural substrates of decoding performance improvement. Changes in P300 characteristics over training, implying training effects, are analyzed only at Cz (line 520). But decoding involves all channels except for 18 frontal ones, as far as I understand. So, using single-channel P300 characteristics to explain improvement in decoding performance would not be sufficient. It is recommended to explore P300 characteristics at all channels involved in decoding to illuminate neural substrates of decoding improvement.

Response:

Thank you very much for raising this question, which helps us better clarify the data analysis part. To address this concern, we added Figures R2 and R3 to show the EEG P300 of all electrodes.

It can be observed that the standard deviation of the P300 amplitude recorded by electrodes in the prefrontal region is very high, indicating that it is extremely sensitive to noise. Therefore, in our online BCI tests, the first two rows of electrodes located over the frontal regions were entirely excluded. These excluded electrodes include: Fp1, Fp2, AF7, AF3, AFz, AF4, and AF8. Additionally, the two outermost temporal electrodes, FT9 and FT10, were also excluded from both online and offline analyses. Regarding the third-row electrodes (Fz and F1–F7), these were found to be less affected by these artifacts. Notably, during eye blinks, no significant artifacts were observed in these channels. Furthermore, as the P300 features are predominantly below 10 Hz, we applied a 10 Hz low-pass filter to

the EEG signals in both online testing and offline analysis to minimize the influence of such artifacts as much as possible. Therefore, in this round of revision, electrodes Fz and F1–F7 were included in the data analysis, as these electrodes were also used for online decoding during the experiment. After incorporating these channels, the results remained consistent with our previous conclusions: electrode Cz continued to exhibit the largest P300 amplitude across all ten participants during the first calibration phase on day 1, thereby serving as a baseline.

Although we ultimately selected the Cz electrode for further analysis, this decision was made only after analyzing the topographic maps, which revealed that Cz was the most representative channel (Fig.2A, D). This approach follows the common practice in EEG studies, where brain topographies are first presented to identify representative electrodes, followed by detailed time-frequency or waveform plots of those selected channels. Given the impracticality of displaying all 64 or 128 channels within the limited space of a journal article, such selective reporting is a good solution. For instance, studies on movement-related cortical activity typically report statistical results from the C3 and C4 electrodes, such as Zhong et al. (2022), and Shu et al. (2018). Therefore, the primary value of these two topographies in Fig 2 is to provide a visual justification for our selection of the Cz channel. This supports the neurophysiological analysis of focusing our statistical analysis on Cz channel. As shown in Fig. 2A and 2D, the brain topographies serve as a presentation of the results from all channels regarding this question.

References:

Zhong, Yucun, et al. "Tactile sensation assisted motor imagery training for enhanced BCI performance: A randomized controlled study." IEEE Transactions on Biomedical Engineering 70.2 (2022): 694-702.

Shu, Xiaokang, et al. "Tactile stimulation improves sensorimotor rhythm-based BCI performance in stroke patients." IEEE Transactions on Biomedical Engineering 66.7 (2018): 1987-1995.

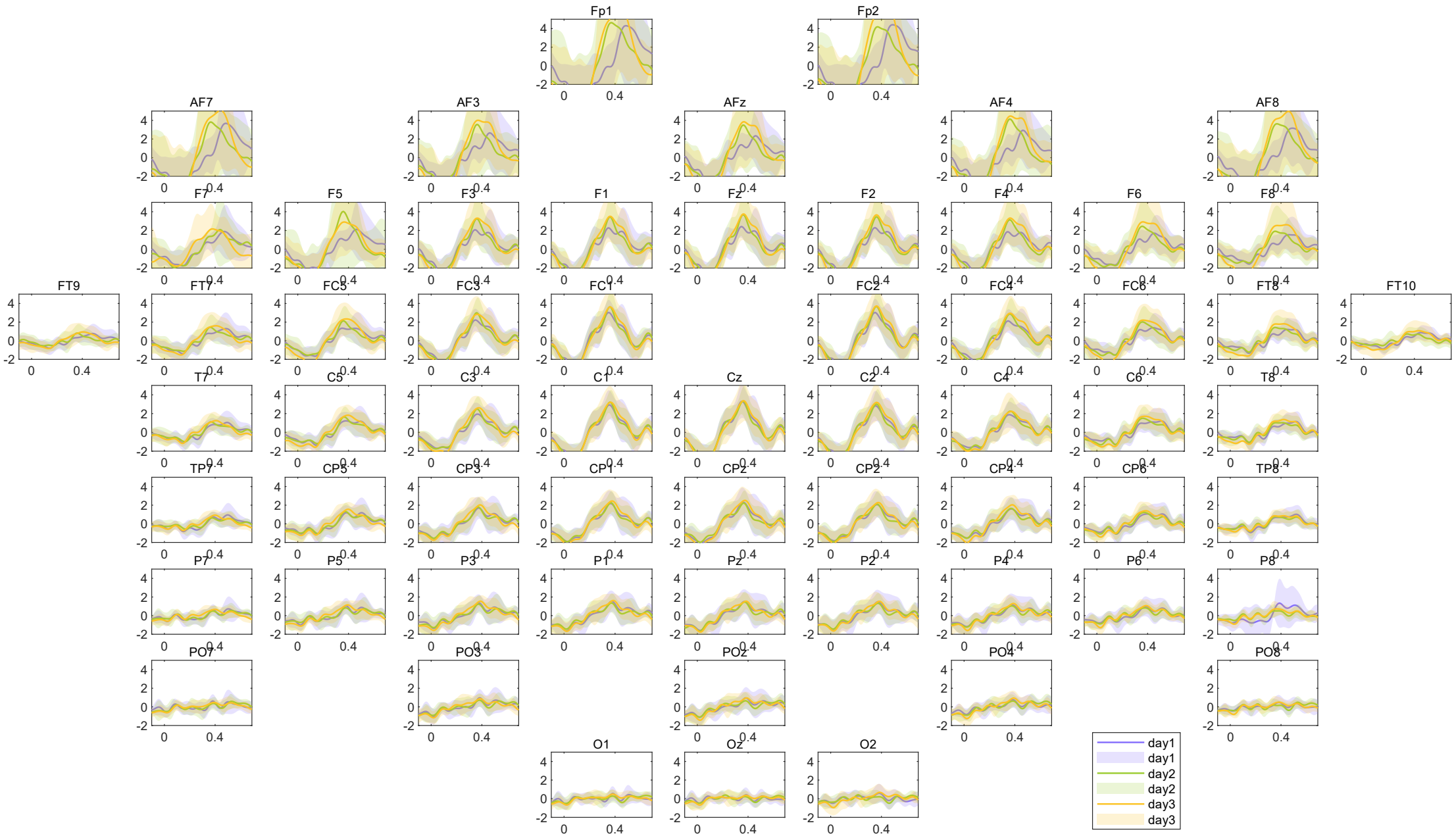

Figure R2. Evoked P300 underlying the tactile-encoded BCI tasks over three days of experiment across 10 participants for single-task condition.

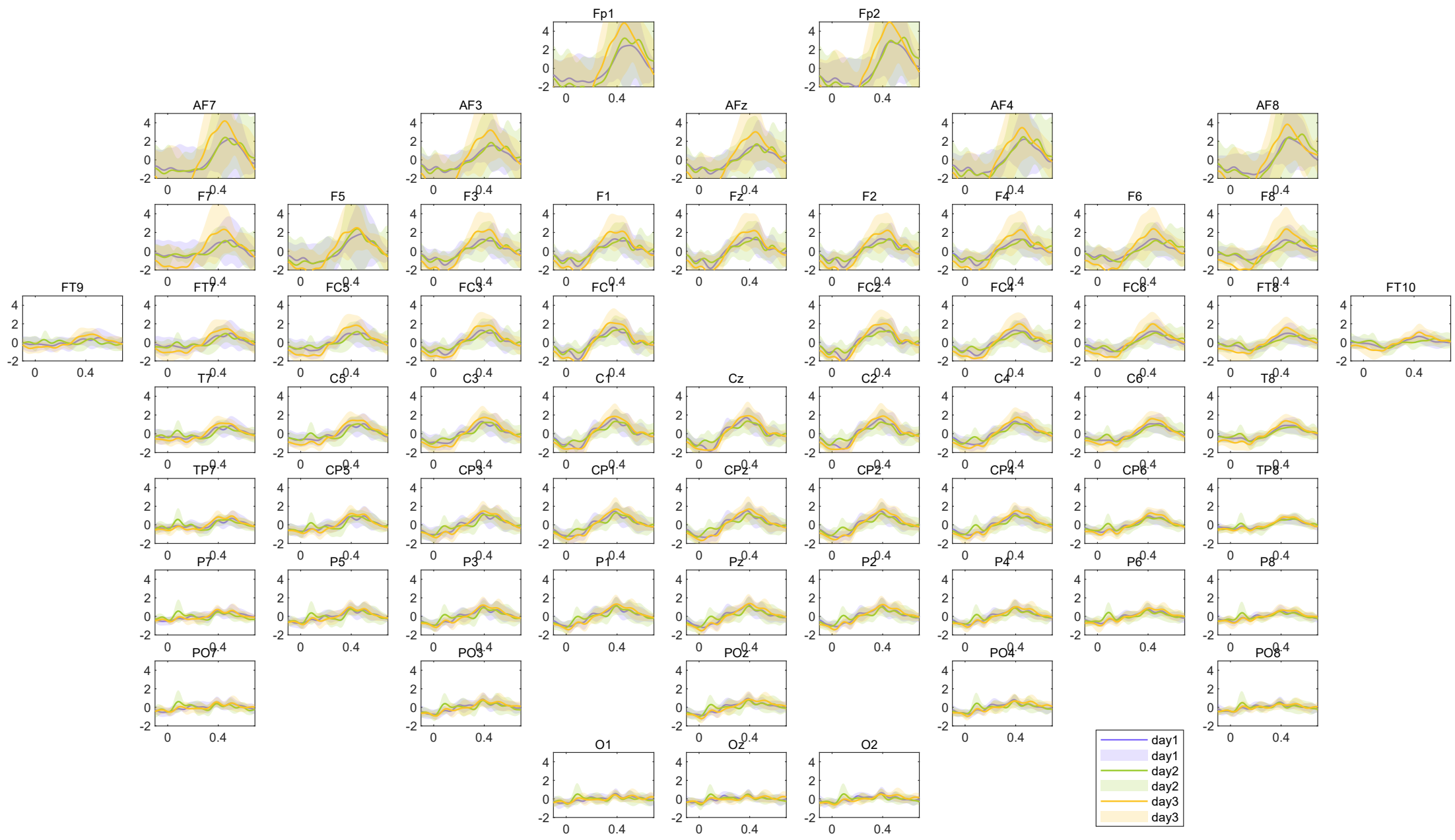

Figure R3. Evoked P300 underlying the tactile-encoded BCI tasks over three days of experiment across 10 participants for dual-task condition.

“

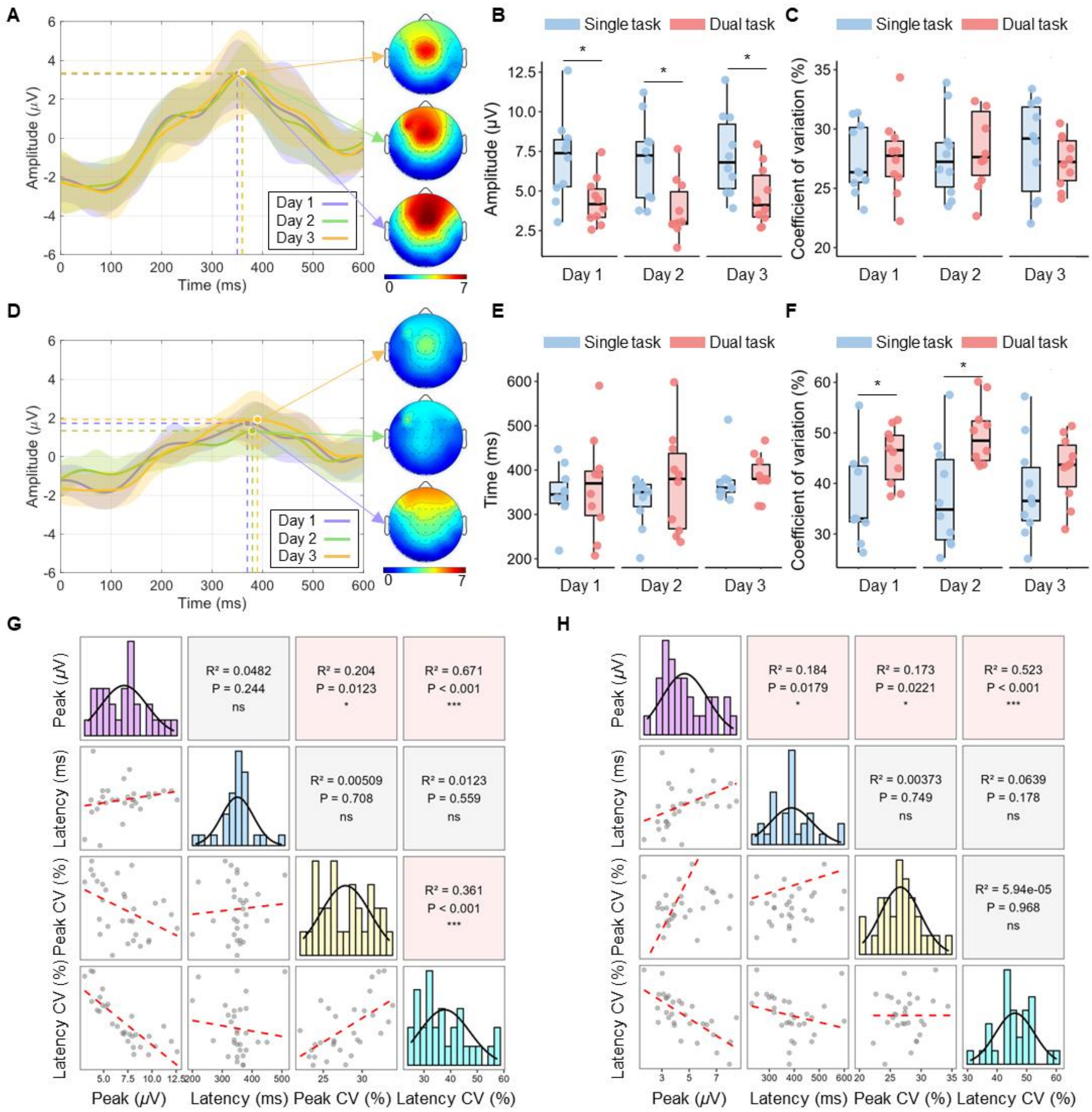

Fig. 2. Evoked P300 underlying the tactile-encoded BCI tasks over three days of experiment across 10 participants. Grand-averaged P300 of Cz for **(A)** single-task and **(D)** dual-task across days 1-3, with corresponding topographic maps illustrating the P300 peak distribution. The solid lines represent the mean amplitude of the P300, and the shaded boundary represents the standard deviation across participants. Boxplots of P300 **(B)** peak amplitude, **(C)** peak coefficient of variation (CV), **(E)** latency, and **(F)** latency CV across days. Correlation matrices of P300 features for **(G)** single-task and **(H)** dual-task conditions, with each dot representing individual data points from each participant per day. With an outlier of the peak CV omitted from the figure but included in statistical analysis. (*if $p < 0.05$).

"

The Reviewer's Comment #6

Natural movement performance evaluation. Since a major contribution of this study is to show that augmented movement does not compromise natural movement, the assessment of natural movement would be crucial. The authors employed the time per ball rotation as a measure of natural movement performance. But why was this measure used? It appears that participants performed the ball balancing task at their own pace. So, it may suggest that individual variations in the time per ball rotation within a condition would be as big as variations across conditions, yielding no statistical difference across conditions (Fig. 4). Since no instruction was given to perform the ball balancing task as quickly as possible, using the time per ball rotation only might not be sufficient. Rather, the authors may consider adding other performance measures. One example can be a success rate of ball rotation.

Response:

We thank the reviewer for highlighting the importance of thoroughly evaluating natural motor performance. We agree that performance measures reflecting successful completion are important in this context. In the ball-balancing task, performance was quantified using two complementary metrics: (i) averaged effective rotation time, calculated as the number of successful rotations divided by total task time, and (ii) averaged gross rotation time, calculated as the total number of rotation attempts, including unsuccessful ones, divided by total task time. The first metric specifically captures the rate of successful ball rotations. Therefore, success rate of ball rotation has been considered and shown using this metric. The second metric additionally incorporates unsuccessful attempts and therefore reflects the overall pace of task execution irrespective of outcome. Together, these two measures provide complementary information on task performance under concurrent BCI use.

More importantly, participants were instructed to perform the task at a steady and controlled pace rather than as quickly as possible, in order to minimize speed–accuracy trade-offs during dual-task conditions. To clarify the rationale and calculation of these metrics, we have expanded the description in the revised manuscript and Supplementary Materials.

"We evaluated two complementary performance metrics: (i) averaged effective rotation time, calculated as the number of successful rotations divided by total task time, and (ii) averaged gross rotation time, calculated as the total number of rotation attempts, including unsuccessful ones, divided by total task time. Detailed definitions and rationale for these metrics are provided in Supplementary Materials. Participants were instructed to perform the task at a steady and controlled pace rather than maximise speed, thereby minimising speed-accuracy trade-offs.

Supplementary Materials: Quantifying ball-balancing performance

Natural movement performance during the ball-balancing task was quantified using two complementary temporal metrics. The first metric, averaged effective rotation time, was

calculated as the total task duration divided by the number of successfully completed rotations. A rotation was considered successful when the ball passed sequentially through all four corners of the tray. This metric reflects execution efficiency conditional on correct task completion. The second metric, averaged gross rotation time, was calculated as the total task duration divided by the total number of rotation attempts, including unsuccessful rotations. Because unsuccessful rotations reduced the effective completion rate, this measure captures the overall temporal stability of task performance and implicitly incorporates the impact of errors. Participants were instructed to perform the task at a steady and controlled pace rather than as quickly as possible. Under these conditions, the two metrics provide a complementary measure of coordination efficiency and stability during concurrent BCI use."

The Reviewer's Comment #7

Analysis of individual differences. It is essential to analyze individual differences. For instance, there can be good performers versus poor performers. If so, knowing what contributed to poor performance would be important. For instance, the analysis of EEG data to assess attentional load in individuals might be helpful.

Response:

We thank the reviewer for the insightful suggestion regarding the analysis of individual differences. We agree that examining performance variability across participants is meaningful. In response, we have conducted a comprehensive individual difference analysis using K-means clustering based on success rates for dual-task runs across days 1-3.

To identify distinct performance subgroups, we performed K-means clustering on the three-dimensional data (Day 1, Day 2, and Day 3 success rates) for all 10 participants. Data were z-score normalized prior to clustering to ensure equal weighting across days. As shown in Figure R1, the optimal number of clusters (k = 3) was determined using the elbow method, which revealed a clear inflection point at three clusters. Clustering was repeated 50 times with different initializations to avoid local optima, and the solution with the lowest within-cluster sum of squares was selected. The stability of the clustering solution was confirmed by a perfect adjusted Rand index (ARI = 1.00) across multiple runs.

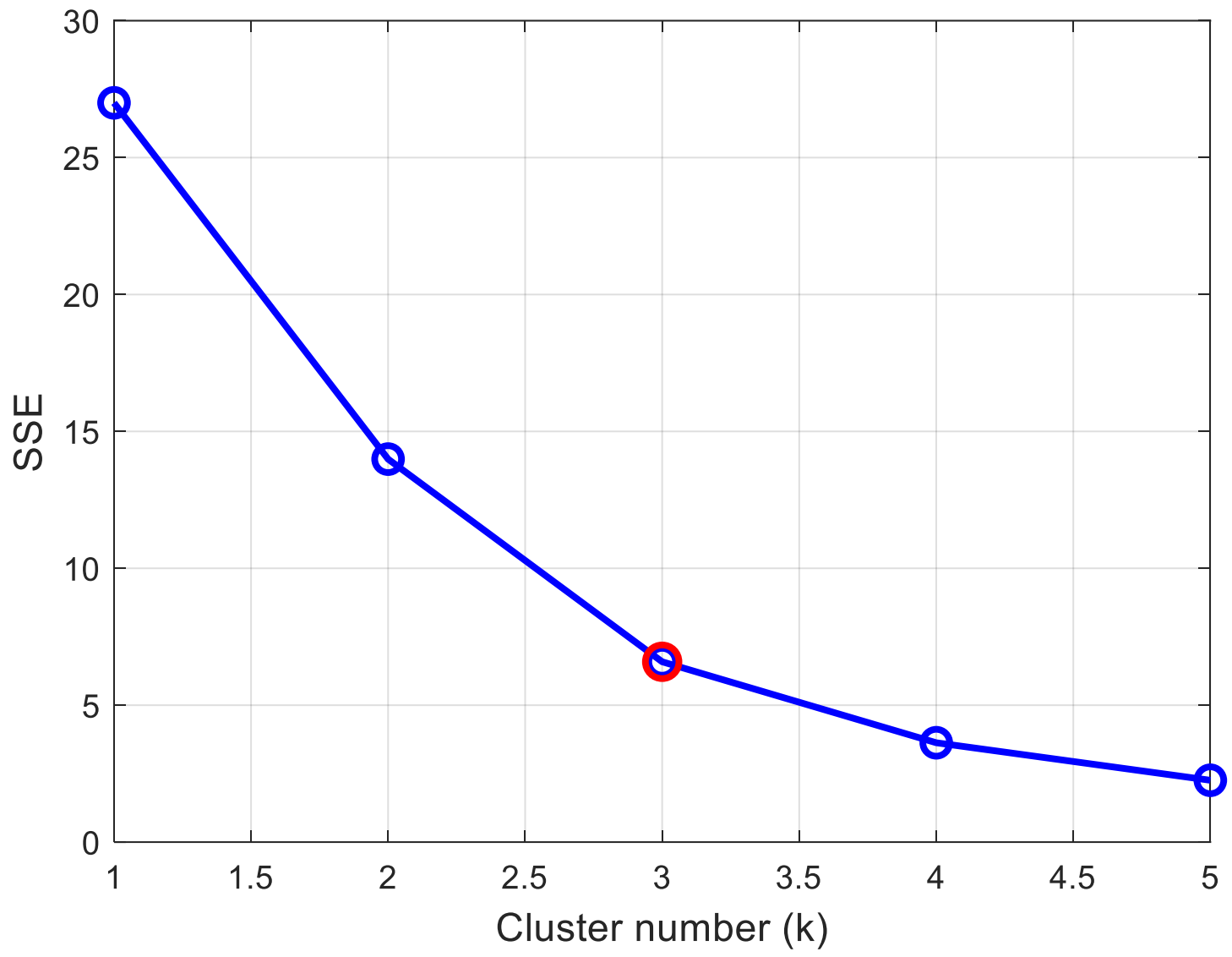

Figure R1. Elbow method to determine the optimal number of clusters.

The K-means clustering analysis revealed three distinct performance profiles, as summarized in Table R1 and Figure S9. High Performers (Cluster 3): Subjects 2, 5, and 6 exhibited consistently high success rates across all three days (overall mean = 76.4%). Notably, this group showed minimal improvement over time (2.1%), suggesting they had reached a performance ceiling from the outset. Intermediate Learners (Cluster 1): Most participants (n = 6) fell into this cluster, characterized by moderate initial performance (Day 1: 45.3%) and substantial improvement across sessions (24.5% improvement), eventually reaching levels comparable to the high performers by Day 3 (69.80%). Low Performers (Cluster 2): Subject 9 was identified as an outlier with persistently low performance (overall mean = 31.3%). This subject's improvement trajectory (12.50% over three days) was substantially shallower than that of the intermediate performers (24.48%), despite starting from a much lower baseline. Notably, while BCI illiteracy has been extensively reported in motor imagery-based BCI, our clustering analysis identified an analogous phenomenon in our proposed tactile-encoded BCI paradigm, with Subject 9 exhibiting this characteristic.

Table R1. Cluster characteristics based on three-day success rates (Mean ± SD)

Cluster No.	Subjects	Success rates (%)				
		Day 1	Day 2	Day 3	Mean	Improvement (Day 3-Day1)
1	1,3,4,7,8,10	45.3±9.2	53.1±10.1	69.8±7.3	56.1	24.5
2	9	28.1	25.0	40.63	31.3	12.5
3	2,5,6	77.1±11.0	72.9±10.0	79.2±7.9	76.4	2.1

Regarding the analysis of EEG data to assess attentional load in individuals, we greatly appreciate the opportunity to consider this important dimension, and this comment really inspires us. However, due to the inherent limitations of the current dataset, resting-state EEG data were not collected in this study. Consequently, it was not feasible to compute

relevant features such as relative power spectral density or other EEG features commonly used to characterize attentional load. We acknowledge this as a limitation of the present work. Nonetheless, we wish to emphasize that the reviewer's valuable suggestion to conduct individual difference analysis has led to an unexpected and noteworthy finding: the identification of BCI illiteracy phenomenon in the context of tactile-encoded BCI. Informed by the reviewer's constructive feedback, we plan to address this question more comprehensively in our future investigations. Specifically, we aim to incorporate resting-state and tactile-encoded BCI control task-state EEG recordings to enable deeper neurophysiological analyses, thereby elucidating the mechanisms underlying performance variability in tactile-encoded BCI. We are grateful for the reviewer's guidance, which has not only improved the current manuscript but also helped us shape the direction of our ongoing research.

"

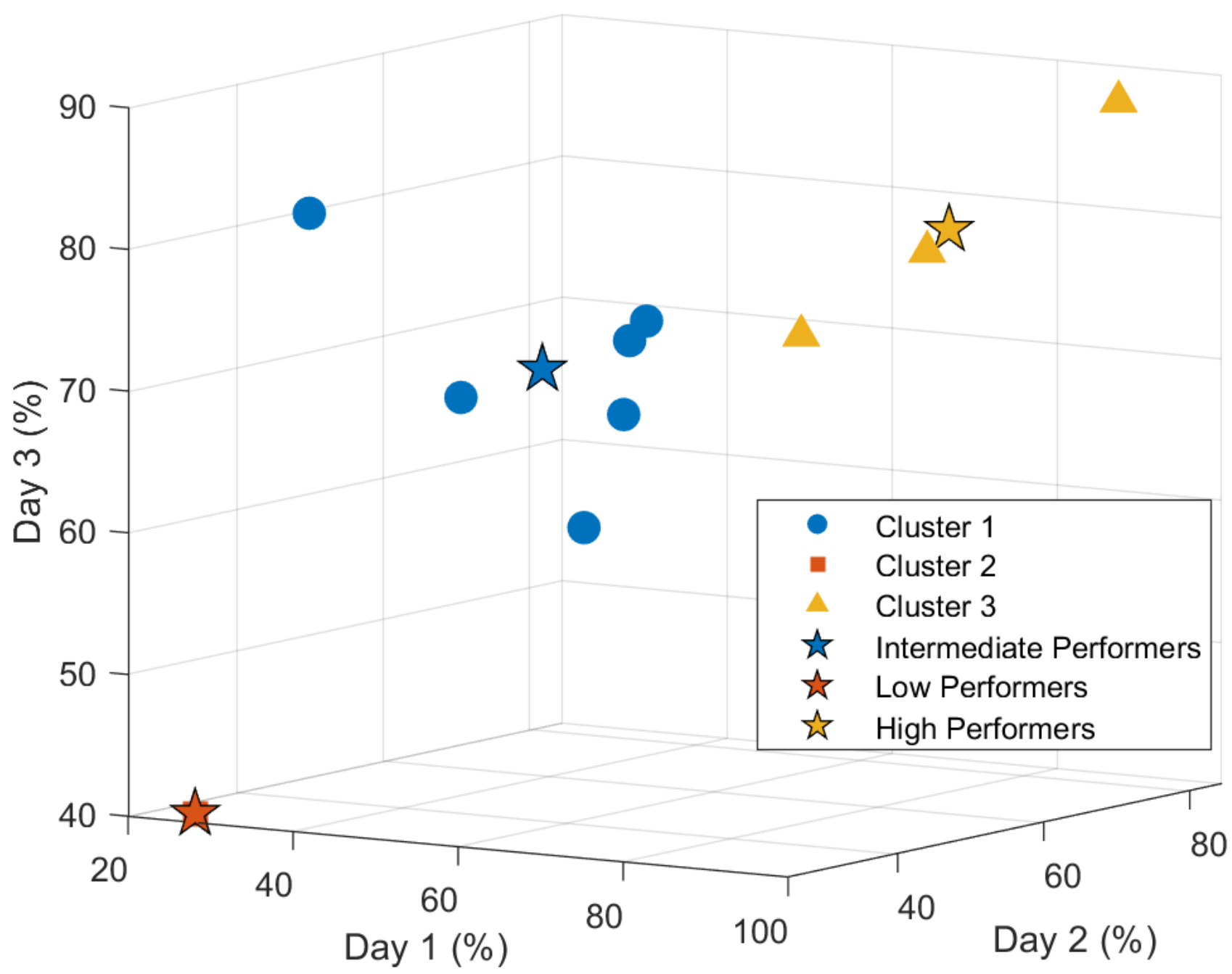

Figure S9. Individual difference analysis using K-means clustering based on success rates of dual-task runs across days 1-3. Three-dimensional scatter plot shows the distribution of 10 participants' success rates on Days 1, 2, and 3. Data points are color-coded by cluster membership (blue: Cluster 1, Intermediate Performer; red: Cluster 2, Low Performer; yellow: Cluster 3, High Performers). One low performer can be regarded as BCI illiteracy of the tactile-encoded BCI."

The Reviewer's Comment #8

BCI performance metrics. One of the BCI performance metrics is the response time. But in fact, this metric counts the number of attempts until the target P300 was detected. It is more natural to measure the response time as the time elapsed until the target P300 was detected in the unit of seconds. For instance, if I understand correctly, when n = 1, the target P300 was detected at the first attempt after the fourth round, which would take 6.4 seconds. Then, the response time should be 6.4 seconds if we ignore the time taken for decoding. Accordingly, while the authors claim that reliable movement augmentation could be achieved with minimal latency (line 376), the minimal latency actually spans 6.4 – 8.0 seconds (first or second attempts), which seems not to be short.

Response:

We sincerely thank you for your careful and detailed review. Your calculation is entirely correct. In the online test paradigm we used, the first recognition attempt indeed requires accumulating four rounds (i.e., 16 trials) for averaging before being fed into the classifier to generate the first output. What's more, it is important to clarify one additional point: each trial duration was set to 0.4 s. Considering that P300 latency can exceed 300 ms across individuals, to ensure complete capture of the P300 waveform, we extended the epoch window by appending 0.4 s of the subsequent trial to the preceding one. Consequently, to ensure sufficient data length for the 16th trial in the first recognition attempt, the system was configured to perform the first classification after the completion of the 18th trial. Therefore, when the recognition attempt is 1, the response time corresponds to 7.2 seconds, with subsequent classification decisions made every 1.6 seconds. To address your concern and improve clarity, we have added this information to the revised manuscript and reported the response time in seconds and included Figure S12 in the Supplementary Materials.

As you have pointed out, we acknowledge that the current minimal latency is indeed not very short. This is primarily because we have adopted an averaging strategy to ensure more stable P300 decoding and BCI control performance. However, it is worth noting that there is a growing body of research on single-trial decoding of P300, which holds promise for further reducing latency. Accordingly, we have supplemented the Discussion section with relevant progress on single-trial decoding. This is also one of our key research focuses for improving the performance of our proposed tactile-encoded BCI.

"Given that the P300 response typically requires trial averaging to achieve a detectable peak, a sliding-window approach was adopted. EEG responses evoked by four consecutive stimuli with the same vibrator were averaged and fed into the decoder. Each stimulation round consisted of four distinct vibration stimuli (one stimulus per vibrator), delivered in a pseudorandom sequence, with a total duration of 1.6 seconds per round. Due to the requirement of trial averaging, the first recognition attempt occurred after the completion of the 18th trial. Thereafter, a decoding decision was made after each set of 4

consecutive trials, resulting in a total of 16 attempts per run.

Further improvements in responsiveness will depend on the advances in single-trial P300 decoding, although such approaches must balance decoding accuracy, computational cost, and real-time feasibility. De Venuto et al. developed an embedded single-trial P300 decoder that increased the BCI responsiveness, while the validation was limited to only speller data *(34)*. Leoni et al. introduced an explainable deep learning pipeline that improved single-trial decoding accuracy with increased computational cost *(35)*. Du et al. reported that fusing multi-subject CNN features boosted the accuracy of single-trial P300 decoding to above 80%, despite requiring large datasets and training overhead *(36)*. Kim et al. demonstrated a calibration-free "plug-and-play" single-trial BCI using pre-trained spatial filters, showing promising real-time use *(37)*. In parallel, Eidel et al. observed that tactile P300 could be recorded using ear-mounted electrodes, highlighting the practical trade-off between improved wearability and reduced signal quality *(38)*. Collectively, these developments suggest that improving single-trial decoding while maintaining high computational efficiency will be important for enhancing the practical performance of our proposed tactile-encoded BCIs."

References:

34. D. De Venuto, G. Mezzina, A single-trial P300 detector based on symbolized EEG and autoencoded-(1D)CNN to improve ITR performance in BCIs. Sensors 21, 3961 (2021).
35. J. Leoni, S. C. Strada, M. Tanelli, A. Brusa, A. M. Proverbio, Single-trial stimuli classification from detected P300 for augmented brain-computer interface: A deep learning approach. Mach. Learn. Appl. 9, 100393 (2022).
36. P. Du, P. H. Li, L. L. Cheng, X. Q. Li, J. X. Su, Single-trial P300 classification algorithm based on centralized multi-person data fusion CNN. Front. Neurosci. 17, 1132290 (2023).
37. J. Kim, S. P. Kim, A plug-and-play P300-based BCI with zero-training application. IEEE Trans. Neural Syst. Rehabil. Eng. 33, 3443-3454 (2025).
38. M. Eidel, M. Pfeiffer, P. Ziebell, A. Kuebler, Recording the tactile P300 with the cEEGrid for potential use in a brain-computer interface. Front. Hum. Neurosci. 18, 1371631 (2024).

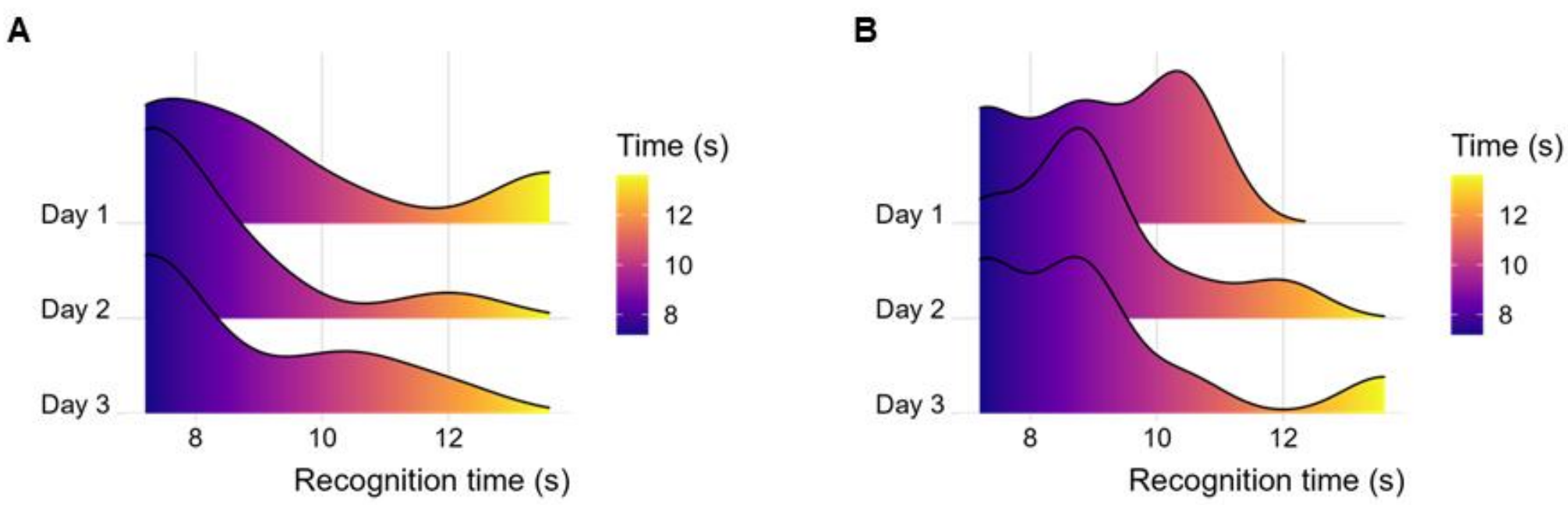

Figure S12. Participant-level distribution of the correct recognition response time (s) of the tactile-encoded BCI over three days of training. (A) single-task and **(B)** dual-task conditions.

The Reviewer's Comment #9

In the title and one-sentence summary, it would be recommended to change "neural interface" to "brain-computer interface" as neural interfaces can describe a broader range of systems than the proposed P300-based BCI.

Response:

Thank you for your suggestion. We totally agree with you that the term "brain-computer interface" is a better choice for this study. We have revised the main title, supplementary material title, and one-sentence summary accordingly.

"Title: Concurrent control of natural and robotic limbs through a tactile-encoded brain-computer interface.

One-Sentence Summary: Tactile-encoded brain-computer interface enables concurrent control of natural and robotic limbs without compromising natural human movement."

The Reviewer's Comment #10

Line 60-62. Current limitations are not clear and need rewriting. Do these illustrate limitations of augmentation by extension via non-invasive signals? How come does redundancy in muscle activity lead to limited effectiveness and difficulties in control? Please consider revising the text.

Response:

We thank the reviewer for this comment. Regarding the limitations discussed in Lines 60–62 (the initial submission version), we confirm that these refer to the constraints of specifically non-invasive peripheral interfaces for augmentation.

We have revised the context in the following ways. Firstly, we temper the idea that non-invasive methods such as EMG offer a "safer, cost-effective, and widely accessible alternative" to invasive intracortical recordings. We have added the word "may" as EMG is only an alternative if it is demonstrated to work, which is a matter of ongoing research:

> "Invasive cortical recording techniques have shown promise for cursor control during simultaneous independent natural movement *(6, 7)*. While effective, non-invasive approaches such as high-density surface electromyography (EMG) and electroencephalography (EEG) may offer a safer, cost-effective, and widely accessible alternative."

Secondly, we add information on the two main approaches to supernumerary control from EMG including their main limitations.

> For instance, Lee et al. investigated whether muscle activation patterns within the wrist's muscle-to-force null-space can be voluntarily modulated to provide independent control signals *(8)*. Although interesting, their findings indicate that this

null-space activity affords at most a single, low-fidelity control dimension that remains tightly coupled to natural wrist movements, limiting its suitability for reliable control. High-frequency inputs to motor neurons, reflected in EMG and largely independent of force production, represent an alternative control source. While these signals have predominantly been modulated using external stimuli (e.g., Go/No-Go paradigms) *(9)*, some evidence suggests they can also be influenced endogenously. For example, Ofner et al. demonstrated modulation of high-frequency components in motor neuron activities through motor imagery and even imagery of non-motor tasks such as mental arithmetic *(10)*. However, the resulting modulation was weak and inconsistent. Consequently, robust strategies for voluntary modulation remain lacking, and the practical applicability of these EMG signals for supernumerary effector control remains uncertain. *(11, 12)*.

References:

6. L. Bashford, J. Wu, D. Sarma, K. Collins, R. P. N. Rao, J. G. Ojemann, C. Mehring, Concurrent control of a brain-computer interface and natural overt movements. J. Neural Eng. 15, 066021 (2018).
7. B. Lansdell, I. Milovanovic, C. Mellema, E. E. Fetz, A. L. Fairhall, C. T. Moritz, Reconfiguring Motor Circuits for a Joint Manual and BCI Task. IEEE Trans. Neural Syst. Rehabil. Eng. 28, 248-257 (2020).
8. M. J. Lee, J. Eden, S. Gurgone, D. J. Berger, D. Borzelli, A. d'Avella, C. Mehring, E. Burdet, Control limitations in the null-space of the wrist muscle system. Sci. Rep. 14, 14 (2024).
9. B. Zicher, S. Avrillon, J. Ibáñez, D. Farina, Changes in high-frequency neural inputs to muscles during movement cancellation. J. Neural Eng. 21, 11 (2024).
10. P. Ofner, M. J. Lee, D. Farina, C. Mehring, Ieee, in 45th Annual International Conference of the IEEE-Engineering-in-Medicine-and-Biology-Society (EMBC). (Ieee, Sydney, AUSTRALIA, 2023).
11. M. J. Lee, J. Eden, S. Gurgone, D. J. Berger, D. Borzelli, A. d'Avella, C. Mehring, E. Burdet, Control limitations in the null-space of the wrist muscle system. Sci. Rep. 14, 20634 (2024).
12. M. Bräcklein, D. Y. Barsakcioglu, A. Del Vecchio, J. Ibáñez, D. Farina, Reading and modulating cortical beta bursts from motor unit spiking activity. J. Neurosci. 42, 3611–3621 (2022).

The Reviewer's Comment #11

Line 70-72. Since SMRs are well known, it may not be necessary to explain SMRs in details. Consider removing this part.

Response:

We agree with the reviewer's assessment regarding SMRs and have revised the description to make it more concise. The discussion of SMRs in the introduction has been streamlined and is now introduced in a single sentence:

"Sensorimotor rhythms (SMRs) from EEG have been widely used for user command recognition in individuals with severe motor disabilities *(14,15)*."

References:

14. J. R. Wolpaw, D. J. McFarland, G. W. Neat, C. A. Forneris, An EEG-based brain-computer interface for cursor control. Electroencephalogr. Clin. Neurophysiol. 78, 252-259 (1991).
15. A. Biasiucci, R. Leeb, I. Iturrate, S. Perdikis, A. Al-Khodairy, T. Corbet, A. Schnider, T. Schmidlin, H. Zhang, M. Bassolino, D. Viceic, P. Vuadens, A. G. Guggisberg, J. D. R. Millán, Brain-actuated functional electrical stimulation elicits lasting arm motor recovery after stroke. Nat. Commun. 9, 2421 (2018).

The Reviewer's Comment #12

Line 78-82. "While MI offers a reliable source of multidimensional neural signals that can be internally modulated, its application in human augmentation is impractical as it requires deciphering supernumerary control intentions that are superimposed on the neural activity associated with natural movement." But Bashford et al. (JNE 2018) demonstrated that humans could learn to dissociate cortical signals associated with the control of natural limb movements and the control supernumerary effectors. Hence, would it be possible for humans to modulate SMRs for both SE control and natural limb control via learning? Please discuss this possibility.

Response:

We thank the reviewer for this helpful comment. We had previously referred to this study, but without explicitly naming the authors:

> "Invasive cortical recording techniques have shown promise for cursor control during simultaneous independent natural movement *(6, 7)*.
>
> **References:**
>
> 6. L. Bashford, J. Wu, D. Sarma, K. Collins, R. P. N. Rao, J. G. Ojemann, C. Mehring, Concurrent control of a brain-computer interface and natural overt movements. J. Neural Eng. 15, 066021 (2018).
> 7. B. Lansdell, I. Milovanovic, C. Mellema, E. E. Fetz, A. L. Fairhall, C. T. Moritz, Reconfiguring Motor Circuits for a Joint Manual and BCI Task. IEEE Trans. Neural Syst. Rehabil. Eng. 28, 248-257 (2020)."

We acknowledge that Bashford et al. (2018) demonstrated that motor execution and motor imagery signals can be separated even when they originate from the same cortical region. However, non-invasive BCI is typically limited to two classes after which the accuracy decreases below usability (70% for 2 classes with modern techniques) However, we hypothesised that our tactile-evoked P300 approach may be better suited for intuitive, multi-dimensional supernumerary control.

Similarly, using non-invasive EEG, Leeb et al. (2013) and Cheung et al. (2012) showed successful BCI control during concurrent movement. However, in these studies, the imagined and executed movements were different. This may be better described as "augmentation by transfer" rather than "augmentation by extension."

To our knowledge, only one study has non-invasively examined imagined movements performed alongside the same real movement. Peñaloza and Nishio (2018) found that imagined grasping could trigger a robotic arm during a bimanual task. However, their analysis of SMR modulation was limited, providing only tentative support for true augmentation. And in their experiment, each subject participated in the experiment only once and did not undergo repeated sessions across multiple days. Therefore, no learning effects of modulating SMRs for both SE control and natural limb control was reported.

To address this gap, we have added a sentence in the Discussion highlighting concurrent identical motor imagery and execution as an important direction for future research.

> "Neural interfaces are increasingly transitioning from discrete, switch-based control towards continuous control paradigms. For real-time BCI applications, control that is continuously proportional to a neural signal is desirable compared to a simple action trigger, as it may allow direct control over the robotic device's range of motion *(41)*. Such control method relies on the ability of users to voluntarily modulate neural rhythms through real-time neural feedback. For example, for endogenous paradigms such as MI, the modulation of SMRs can be utilised to drive continuous control. However, whether users can learn to independently modulate sensorimotor rhythms for concurrent supernumerary control and identical natural limb movement using non-invasive EEG remains an open question, representing a critical direction for future investigation."

References:

2. C. I. Penaloza, S. Nishio, BMI control of a third arm for multitasking. Sci. Robot. 3, eaat1228 (2018).
6. L. Bashford, J. Wu, D. Sarma, K. Collins, R. P. N. Rao, J. G. Ojemann, C. Mehring, Concurrent control of a brain-computer interface and natural overt movements. J. Neural Eng. 15, 066021 (2018).
7. B. Lansdell, I. Milovanovic, C. Mellema, E. E. Fetz, A. L. Fairhall, C. T. Moritz, Reconfiguring Motor Circuits for a Joint Manual and BCI Task. IEEE Trans. Neural Syst. Rehabil. Eng. 28, 248-257 (2020).
16. R. Leeb, M. Lancelle, V. Kaiser, D. W. Fellner, G. Pfurtscheller, Thinking Penguin: Multimodal Brain-Computer Interface Control of a VR Game. IEEE Trans. Comput. Intell. AI Games 5, 117-128 (2013).
17. W. Cheung, D. Sarma, R. Scherer, R. P. N. Rao, Ieee, in 34th Annual International Conference of the IEEE Engineering-in-Medicine-and-Biology-Society (EMBS). (Ieee, San Diego, CA, 2012), pp. 6715-6718.
41. B. J. Edelman, J. Meng, D. Suma, C. Zurn, E. Nagarajan, B. S. Baxter, C. C. Cline, B. He,

Noninvasive neuroimaging enhances continuous neural tracking for robotic device control. Sci. Robot. 4, eaaw6844 (2019).

The Reviewer’s Comment #13
Line 85-94. Explanation of the P300 speller is too long. This part can be shortened.

Response:
We thank the reviewer for this suggestion and have modified this part of the Introduction to have a more concise explanation of the P300 speller.

“The classic Farwell & Donchin "P300 Speller" enables users to communicate sequences of letters to a computer by exploiting this response *(19, 20)*. In this paradigm, attending to an infrequent target flash elicits a P300 that can be decoded to infer the intended letter, typically achieving accuracies above 80% at around 7-8 characters per minute *(21, 22)*.”

References:
19. L. A. Farwell, E. Donchin, Talking off the top of your head: Toward a mental prosthesis utilizing event-related brain potentials. Electroencephalogr. Clin. Neurophysiol. 70, 510-523 (1988).
20. E. W. Sellers, E. Donchin, A P300-based brain-computer interface: Initial tests by ALS patients. Clin. Neurophysiol. 117, 538-548 (2006).
21. J. H. Pan, X. N. Chen, N. M. Ban, J. S. He, J. Y. Chen, H. Y. Huang, Advances in P300 brain-computer interface spellers: toward paradigm design and performance evaluation. Front. Hum. Neurosci. 16, 1077717 (2022).
22. E. Donchin, K. M. Spencer, R. Wijesinghe, The mental prosthesis: Assessing the speed of a P300-based brain-computer interface. IEEE Trans. Rehabil. Eng. 8, 174-179 (2000).

The Reviewer’s Comment #14
Please provide rationale to choose body locations for tactile stimulation.

Response:
The rationale for selecting body locations for tactile stimulation is as follows: This study focuses on controlling an additional robotic arm, so the vibrators were placed on both upper arms to correspond with the two robotic arms, rather than on the torso or lower limbs. The placement was chosen to avoid interfering with natural finger movements, so the hands were excluded from potential placement. To prevent bias caused by asymmetric placement on the upper limbs, the vibrators were positioned symmetrically near the elbow and wrist joints on both sides. This explanation has been added to the revised Supplementary Materials.

“**Supplementary Materials: Tactile vibrator setup**
Four C2-HDLF vibrators (Engineering Acoustics Inc., Casselberry, FL, USA) were secured bilaterally to the participants’ arms using hypoallergenic medical tape (Fig. S3). The vibrators were placed on both upper arms to correspond with the two robotic arms, rather

than on the torso or lower limbs. The hands were excluded as potential placement sites to avoid interfering with natural finger movements. The vibrators were positioned symmetrically near the elbow and wrist joints on both sides to minimise proprioceptive bias and ensure balanced perception across both arms."

The Reviewer's Comment #15

Line 125. Is it correct to say that tactile P300 BCI is used to recognize motor intention? In fact, this description is found across the manuscript. I agree that the tactile P300 BCI in this study is used for recognizing the user's intention of robot control. But motor intention is somewhat different, typically meaning the intention of movement encoded in relevant EEG signatures, not P300 per se. So, using the term, "motor intention" in tactile P300 BCIs can be misleading. Please consider revising the corresponding descriptions.

Response:

We appreciate the reviewer's comment and agree that our original phrasing could lead to misunderstanding. While the P300 signal in our paradigm serves as a proxy for intended motor commands for controlling the supernumerary DoFs, we acknowledge that the P300 itself does not originate from cortical sensorimotor processes. To better reflect this distinction, we have replaced the term "motor intention recognition" with "user command recognition," which more accurately captures the role of the signal in our paradigm.

The text has been revised to read:

> "…this study investigated (i) whether a tactile-encoded BCI can be utilized for user command recognition and DoF augmentation…"

In addition, the term "motor intention recognition" has been replaced with "user command recognition" throughout the manuscript.

The Reviewer's Comment #16

Line 126-127. Another important question of this study would be whether the tactile-encoded BCI interferes with natural movement. Consider adding this question.

Response:

Thank you for your valuable suggestion. The performance of the ball-balancing task, as reported in Figure 4, addresses this question. Accordingly, we have incorporated this question into the revised manuscript at the end of Introduction part.

"Overall, this study investigated (i) whether a tactile-encoded BCI can be utilised for user command recognition and DoF augmentation, (ii) whether the performance of the tactile-encoded BCI improves with training, (iii) whether the tactile-encoded BCI interferes with natural movement, and (iv) the performance of a tactile-encoded BCI used for functional

movement augmentation tasks.”

The Reviewer’s Comment #17

Was the mapping between stimuli and commands randomized across participants? Please clarify.

Response:

We thank the reviewer for this question. The mapping between the vibrotactile stimuli and the corresponding control commands was kept consistent across participants throughout the experiment. Each vibrator location was associated with a predefined command to simplify the learning process and maintain consistency across sessions and participants. We have clarified this mapping procedure in the revised manuscript.

“Each vibrator location was associated with a predefined command throughout the experiment. This mapping was kept consistent across participants to facilitate learning across sessions, while the presentation order of the vibrotactile stimuli within each run was pseudorandomized according to the oddball paradigm.”

The Reviewer’s Comment #18

The P300 amplitudes in Fig. 2A are near 4 uV while those for the single task in Fig. 2B are around 7uV. Please explain this discrepancy.

Response:

Thank you very much for your careful review of our manuscript. We have thoroughly examined the analysis data and code, and we confirm that the results presented in the figures are accurate. In Figures 2A and 2D, the P300 waveforms represent the grand-averaged results across all subjects, with the shaded areas indicating the standard deviation of the 10 individual P300 curves at each time point. As shown in Figure 2E, there is substantial variability in P300 latencies across subjects, which leads to misalignment in the timing of the peak P300 amplitudes when individual curves are averaged. Consequently, the peak amplitude in the grand-averaged waveform is inevitably reduced. The latency indicated in Figures 2A and 2D corresponds to the average latency across subjects, and the P300 amplitude is measured at that specific time point. In contrast, Figure 2B presents the maximum P300 amplitude for each subject, measured at their individual peak latency. Therefore, this approach captures the subject-level maximum P300 amplitudes. This difference in methodology accounts for the apparent discrepancy between the amplitude values in Figures 2A, 2D and 2B. Both representations are meaningful and serve different purposes: Figures 2A and 2D illustrate the overall trend of P300 across all subjects, while Figure 2B reveals the distribution of P300 amplitudes at the subject level. Thank you again for your valuable comment. We hope this explanation clarifies the point.

The Reviewer's Comment #19
Please indicate the sample size inside Fig. 2 or in the caption.

Response:
Thank you for your suggestion. In this study, we recruited 10 participants for the proposed tactile-encoded brain-computer interface training and testing. Therefore, the sample size of this study is 10. This information has been added to the caption of Fig.2.

"**Fig. 2. Evoked P300 underlying the tactile-encoded BCI tasks over three days of experiment across 10 participants.** Grand-averaged P300 of Cz for **(A)** single-task and **(D)** dual-task across days 1-3, with corresponding topographic maps illustrating the P300 peak distribution. The solid lines represent the mean amplitude of the P300, and the shaded boundary represents the standard deviation across participants. Boxplots of P300 **(B)** peak amplitude, **(C)** peak coefficient of variation (CV), **(E)** latency, and **(F)** latency CV across days. Correlation matrices of P300 features for **(G)** single-task and **(H)** dual-task conditions, with each dot representing individual data points from each participant per day. With an outlier of the peak CV omitted from the figure but included in statistical analysis. (*if $p < 0.05$)."

The Reviewer's Comment #20
Please indicate the EEG channel for Fig. 2A, D in the caption.

Response:
Figure 2A and 2D show the grand-averaged P300 of Cz. This information has been added to the caption.

"**Fig. 2. Evoked P300 underlying the tactile-encoded BCI tasks over three days of experiment across 10 participants.** Grand-averaged P300 of Cz for **(A)** single-task and **(D)** dual-task across days 1-3, with corresponding topographic maps illustrating the P300 peak distribution. The solid lines represent the mean amplitude of the P300, and the shaded boundary represents the standard deviation across participants. Boxplots of P300 **(B)** peak amplitude, **(C)** peak coefficient of variation (CV), **(E)** latency, and **(F)** latency CV across days. Correlation matrices of P300 features for **(G)** single-task and **(H)** dual-task conditions, with each dot representing individual data points from each participant per day. With an outlier of the peak CV omitted from the figure but included in statistical analysis. (*if $p < 0.05$)."

The Reviewer's Comment #21
What do dots in Fig. 2G-H represent?

Response:
In this study, 10 participants were recruited, and each participant underwent the experiment three times on three separate days. The dots in Fig. 2G-H represent individual data points from each participant per day. This information has been clarified in the figure caption.

“**Fig. 2. Evoked P300 underlying the tactile-encoded BCI tasks over three days of experiment across 10 participants.** Grand-averaged P300 of Cz for **(A)** single-task and **(D)** dual-task across days 1-3, with corresponding topographic maps illustrating the P300 peak distribution. The solid lines represent the mean amplitude of the P300, and the shaded boundary represents the standard deviation across participants. Boxplots of P300 **(B)** peak amplitude, **(C)** peak coefficient of variation (CV), **(E)** latency, and **(F)** latency CV across days. Correlation matrices of P300 features for **(G)** single-task and **(H)** dual-task conditions, with each dot representing individual data points from each participant per day. With an outlier of the peak CV omitted from the figure but included in statistical analysis. (*if $p < 0.05$).”

The Reviewer’s Comment #22

In Fig. 2A, D, EEG channels showing high amplitudes in the topology are distributed in the frontocentral middle area. Typically, Pz is a key channel eliciting P300. Please discuss why Pz exhibits relatively low amplitudes in this study.

Response:

We thank the reviewer for this comment, which prompted us to revisit Fig. 2A and D and to re-analyse the apparent strong frontocentral contribution to the P300 signal. Upon re-examination, we determined that this topographic distribution may be partially misleading due to the removal (and subsequent interpolation) of the frontal electrodes. These channels had originally been excluded from the classification pipeline to minimise interference from ocular artefacts. However, to provide a more representative topographic depiction, we have now reinstated these channels in the revised Fig. 2. As a result, the updated topographic plots show reduced frontocentral dominance. Figures R2 and R3 show the EEG P300 of all electrodes. It can be observed that the amplitude at channel Pz remains relatively lower than at channel Cz, which served as the primary channel of analysis in this study. This pattern is consistent with prior literature on tactile paradigms eliciting P300 responses. For example, Li et al. (2019), Han et al. (2020) and Chu et al. (2021) similarly reported comparatively higher tactile-evoked P300 amplitudes at Cz than at Pz.

References:

Han, X., Niu, J., & Guo, S. (2020). A tactile-based brain computer interface P300 paradigm using vibration frequency and spatial location. *Journal of Medical and Biological Engineering*, *40*(6), 773-782.

Li, Jianing, Jiangbo Pu, Hongyan Cui, Xiaobo Xie, Shengpu Xu, Ting Li, and Yong Hu. (2019). An online P300 brain–computer interface based on tactile selective attention of somatosensory electrical stimulation. *Journal of Medical and Biological Engineering*.

Chu, C., Luo, J., Tian, X., Han, X., & Guo, S. (2021). A P300 brain-computer interface paradigm based on electric and vibration simple command tactile stimulation. *Frontiers in Human Neuroscience*, *15*, 641357.

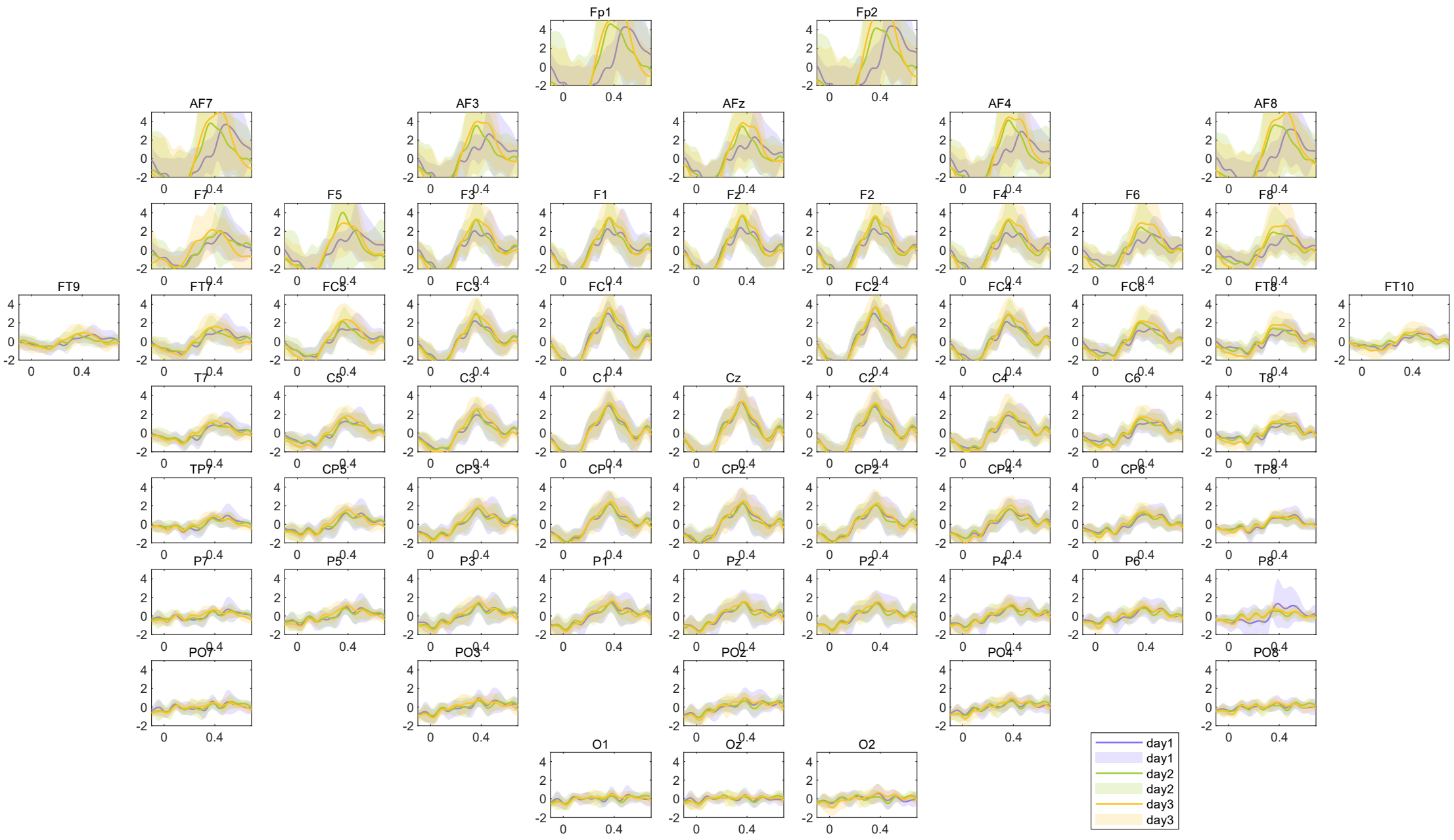

Figure R2. Evoked P300 underlying the tactile-encoded BCI tasks over three days of experiment across 10 participants for single-task.

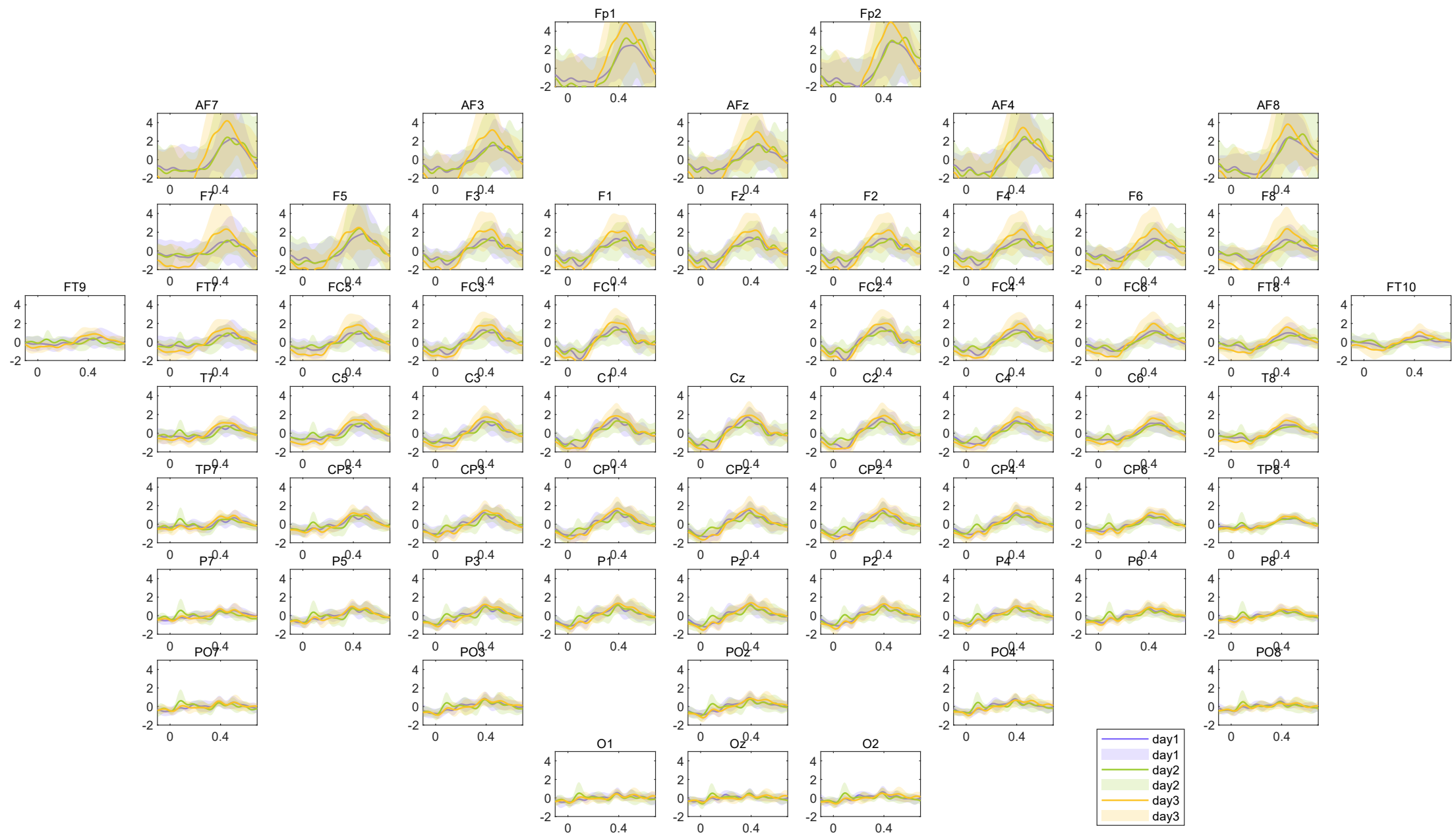

Figure R3. Evoked P300 underlying the tactile-encoded BCI tasks over three days of experiment across 10 participants for dual-task.

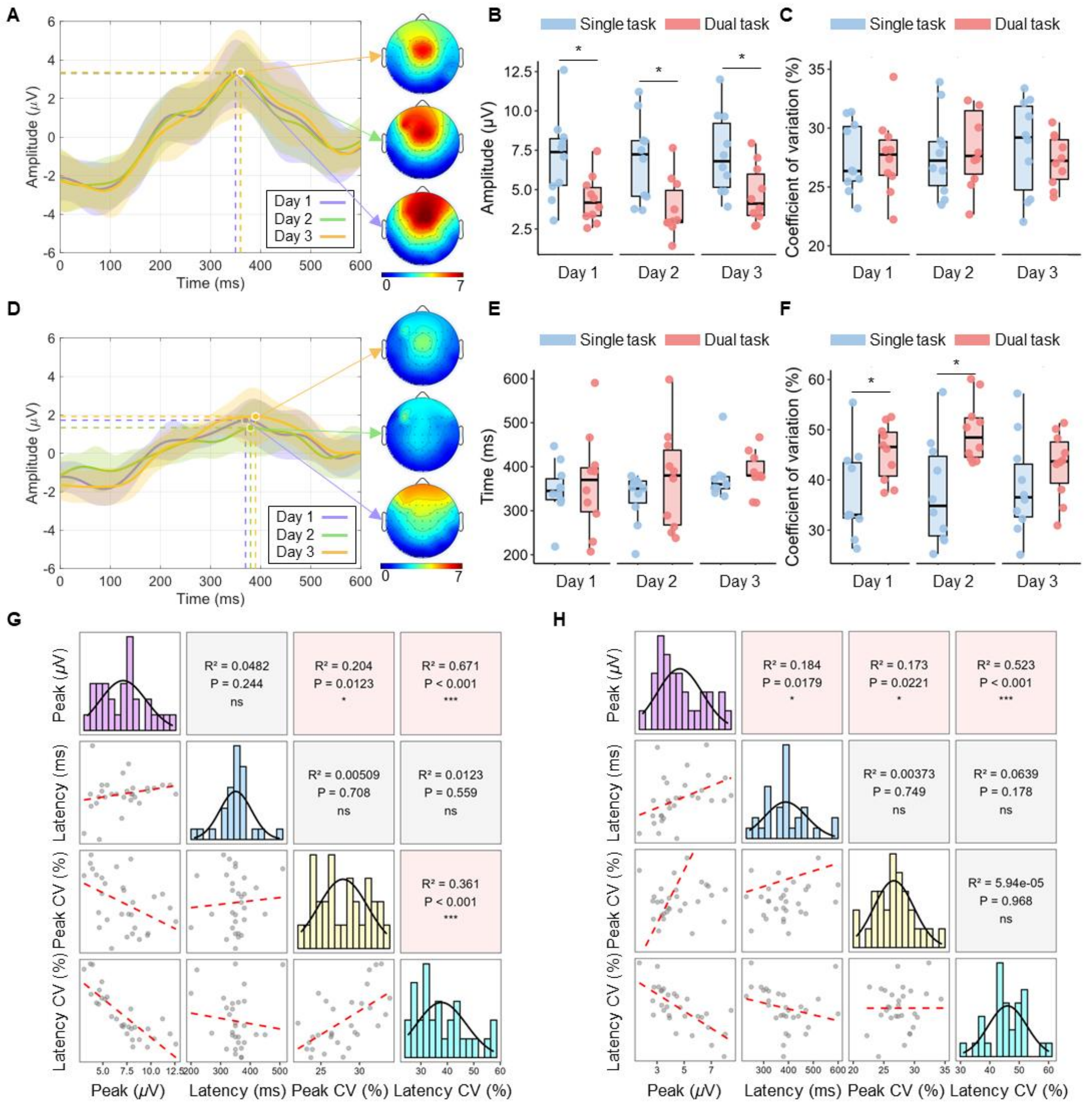

Fig. 2. Evoked P300 underlying the tactile-encoded BCI tasks over three days of experiment across 10 participants. Grand-averaged P300 of Cz for **(A)** single-task and **(D)** dual-task across days 1-3, with corresponding topographic maps illustrating the P300 peak distribution. The solid lines represent the mean amplitude of the P300, and the shaded boundary represents the standard deviation across participants. Boxplots of P300 **(B)** peak amplitude, **(C)** peak coefficient of variation (CV), **(E)** latency, and **(F)** latency CV across days. Correlation matrices of P300 features for **(G)** single-task and **(H)** dual-task conditions, with each dot representing individual data points from each participant per day. With an outlier of the peak CV omitted from the figure but included in statistical analysis. (*if $p < 0.05$).

The Reviewer's Comment #23

Line 164-165. "the comparable P300 latency observed between single- and dual-BCI tasks on the last training day essentially revealed that both tasks exhibit similar performance after training." The comparable P300 latency between the two tasks was observed on all training days (Fig. 2E). So, why this result reflect similar performance after training?

Response:

We apologize for the error in our expression. The intended meaning was that the coefficient of variation (CV) of the P300 latency was comparable between the two tasks, as shown in Fig. 2F. However, after adding further analysis to explore the main effect of time on CV of P300 latency (Figure S5), we realized that concluding a learning effect in BCI control based solely on changes in CV of P300 latency would be overly strong, as BCI learning effects are likely driven by multiple factors beyond this single measure. Accordingly, in the revised manuscript, the sentence has been deleted from the manuscript and we have also modified other corresponding text to temper the original conclusion. Figure S5 has also been added to the Supplementary Materials. Thank you again for your insightful comments, which have helped us improve the rigor of our analysis and the clarity of our claims.

"We thus hypothesise that through training, participants learned to effectively allocate attention during the dual BCI tasks, leading to more stabilised P300 latency. However, it was not possible to infer a learning effect in BCI control solely from changes in the CV of the P300 latency, especially because other metrics of performance, such as information transfer rate (ITR), did not fully correlate with CV.

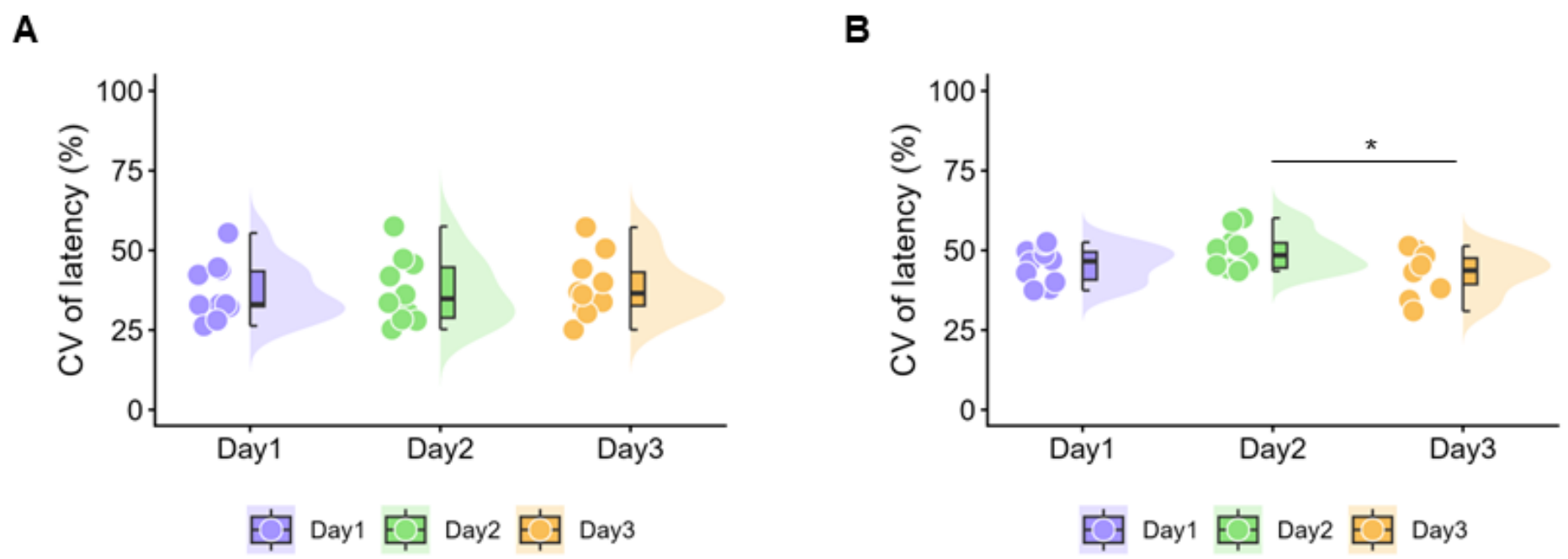

Figure S5. Coefficient of variation (CV) of P300 latency over three days of training. **(A)** single-task and **(B)** dual-task conditions. Violin plots show participant-level distributions with embedded boxplots. (* if $p < 0.05$; one-way repeated-measures ANOVA)

The Reviewer's Comment #24

In Fig. 3D-E, the distribution of the number of attempts extends to 0. Although these distribution curves may be obtained by smoothing histograms (I am not sure though because no explanation is given), zero attempt does not make a sense. So, consider drawing histograms instead or modifying the distribution curves.

Response:

We agree that the extension of the distribution curves to zero was misleading. To address this issue, we have now constrained the x-axis to the range of the actual data in the revised Fig 3. This revision eliminates the misleading smoothing effect and ensures that the curves accurately reflect the data distribution.

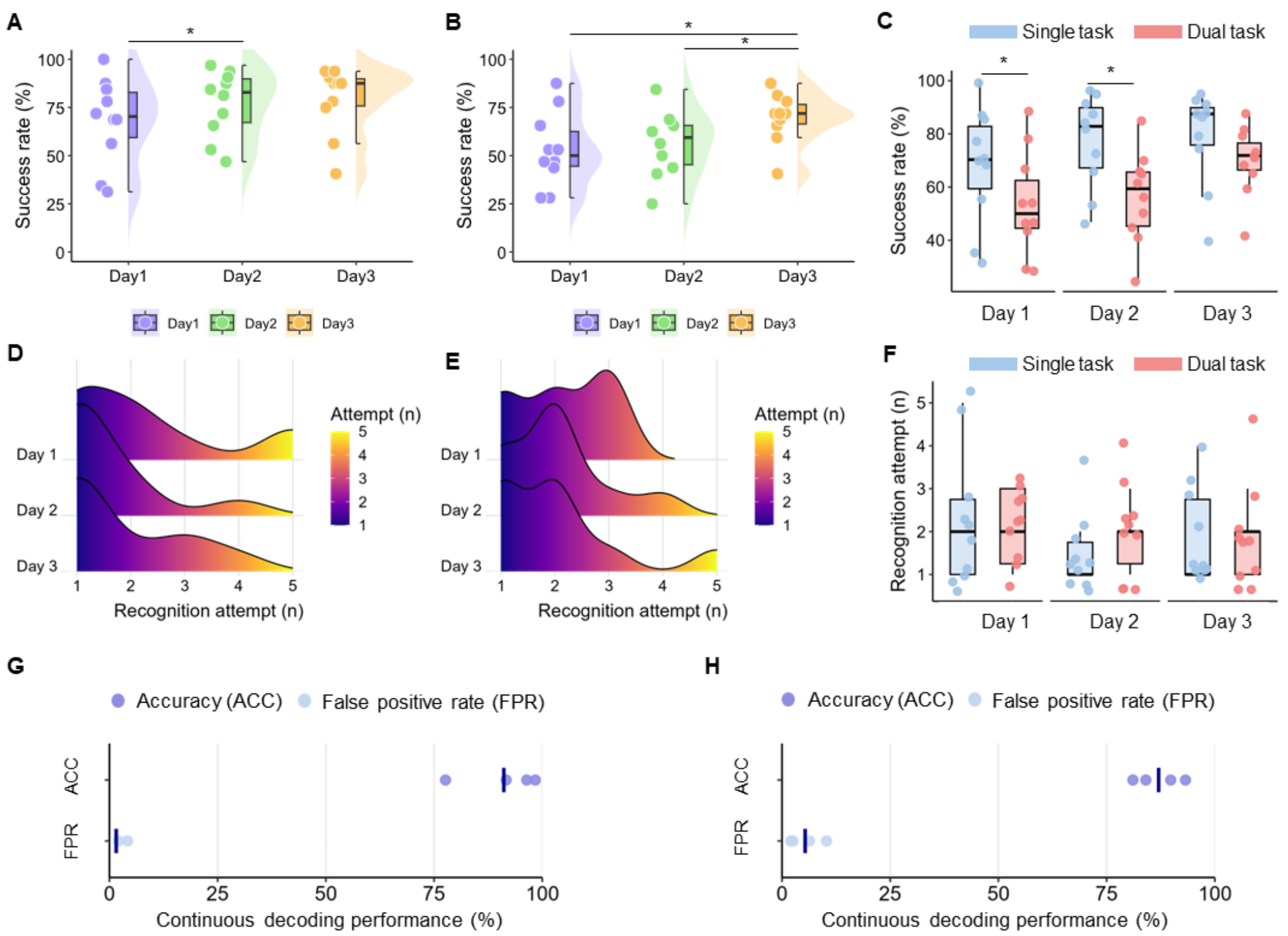

Fig. 3. Online performance of tactile-encoded BCI over three days of experiment, comparing single- and dual-task conditions. Success rates for **(A)** single-task and **(B)** dual-task runs across days 1-3. **(C)** Comparisons of success rate between single- and dual-task conditions on each day. Distribution of the number of recognition attempts before first correct recognition for **(D)** single-task and **(E)** dual-task runs across days. **(F)** Comparisons of recognition attempt between single- and dual-task conditions on each day. Continuous decoding performance metrics for **(G)** single-task and **(H)** dual-task conditions, expressed as accuracy (ACC), and false positive rate (FPR). Each point represents performance from an individual participant. (* if $p < 0.05$)

The Reviewer's Comment #25

Line 282-284. "By combining the evoked P300 with prior tactile encoding, the proposed BCI augmented the controllable DoFs as many as the number of tactile vibrators, …" I am not sure about this. There are four vibrators but the number of commands (controllable DoFs) is three (see Fig. 1). Did the authors count "do nothing" as an independent controllable DoF? If so, please explain it.

Response:

We apologise for the misunderstanding caused by the presentation in the Fig.1. The four

tactile vibrators correspond to four binary classifications, i.e., whether a P300 is detected or not. If a P300 is detected, the control command associated with that vibrator is executed; if not, no command is executed. In the case where none of the four vibrators elicit a detectable P300, the system executes a "do nothing" command. However, we did not count “do nothing” as an independent controllable DoF. We apologize for the confusion caused by the original version of Fig. 1, which has been revised accordingly.

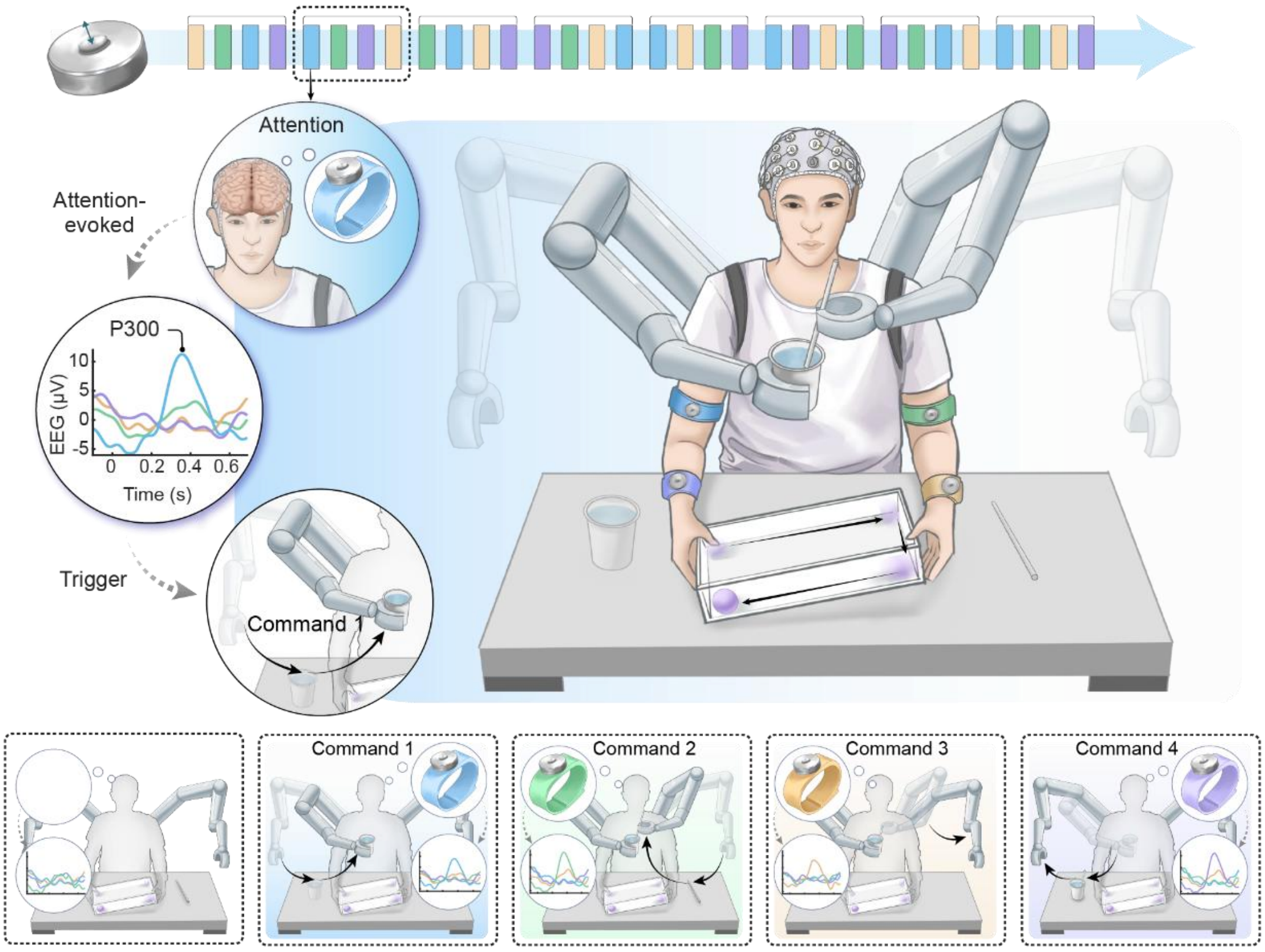

Fig. 1. Experimental setup for tactile-encoded BCI control of supernumerary robotic arms. Four tactile vibrators were attached bilaterally to the upper and lower arms, each delivering 200-ms bursts of vibration with 200-ms interstimulus intervals in a pseudorandom order. Each vibrator corresponded to one of four selectable control states, with symmetric placement to avoid proprioceptive bias. Attended stimuli evoked P300 responses in the EEG, which were decoded online to trigger corresponding robotic commands. A transparent tray was used for the ball-balancing task in the dual-task condition.”

The Reviewer’s Comment #26

Line 479-481. It read that the classification of ERPs in response to each stimulus was conducted individually, yielding four classification outcomes. Then, it is possible that more than one outcome can be the detection of P300. In such cases, how to decide which stimulus was a target?

Response:

The classifier's output includes both the category and a confidence score. A confidence

score greater than zero indicates the detection of a P300, while a score less than zero indicates no P300 detected. In cases where more than one outcome shows the detection of a P300, the outcome with the highest confidence score is selected as the recognized target. This clarification has been added to the revised Supplementary Materials.

"**Supplementary Materials: Online BCI testing and its performance evaluation**
During the online decoding, feature extraction was performed using the xDAWN algorithm and then classified using a support vector machine (SVM). The classifier's output includes both the category and a confidence score. A confidence score greater than zero indicates the detection of a P300, while a score less than zero indicates no P300 detected. In cases where more than one outcome shows the detection of a P300, the outcome with the highest confidence score is selected as the recognised target."

The Reviewer's Comment #27
Line 485-486. "The number of recognition attempts required until the target P300 was detected was recorded as the response time." But in an unsuccessful test, P300 would be elicited by non-target stimuli. In this case, the response time was not recorded? In other words, the response time was recorded only for successful tests? Then, what was the average response time for unsuccessful tests with false non-target recognition?

Response:
Thank you for your question. The response time for unsuccessful tests with false non-target recognition was indeed recorded. However, it is important to clarify that if no false non-target recognition occurred during a test, there would be no corresponding response time for that specific condition. In such cases, the response time is denoted as "NaN" in Table R1:

Table R1. False non-target recognition attempt (n)

	single task			dual task		
Sub No.	Day 1	Day 2	Day 3	Day 1	Day 2	Day 3
1	NaN	NaN	5	NaN	NaN	1
2	1	1	3	3	1	1
3	2	3	2	5	2	1
4	2	2	1	1	2	2
5	1	1	5	2	2	1
6	NaN	NaN	4	3	2	2
7	5	1	NaN	2	1	4
8	5	2	2	3	3	4
9	2	1	1	3	2	3
10	4	1	1	3	3	2

The Reviewer's Comment #28

Since there could be two cases when the test failed, including false non-target recognition and missed recognition, it is essential to report the rates of each failure separately.

Response:

Thank you for your suggestions. False non-target recognition and missed-recognition rates are shown in Figure S7 and Figure S8 which have been added to the revised Supplementary Materials. The values can be also found in the Data file S1.

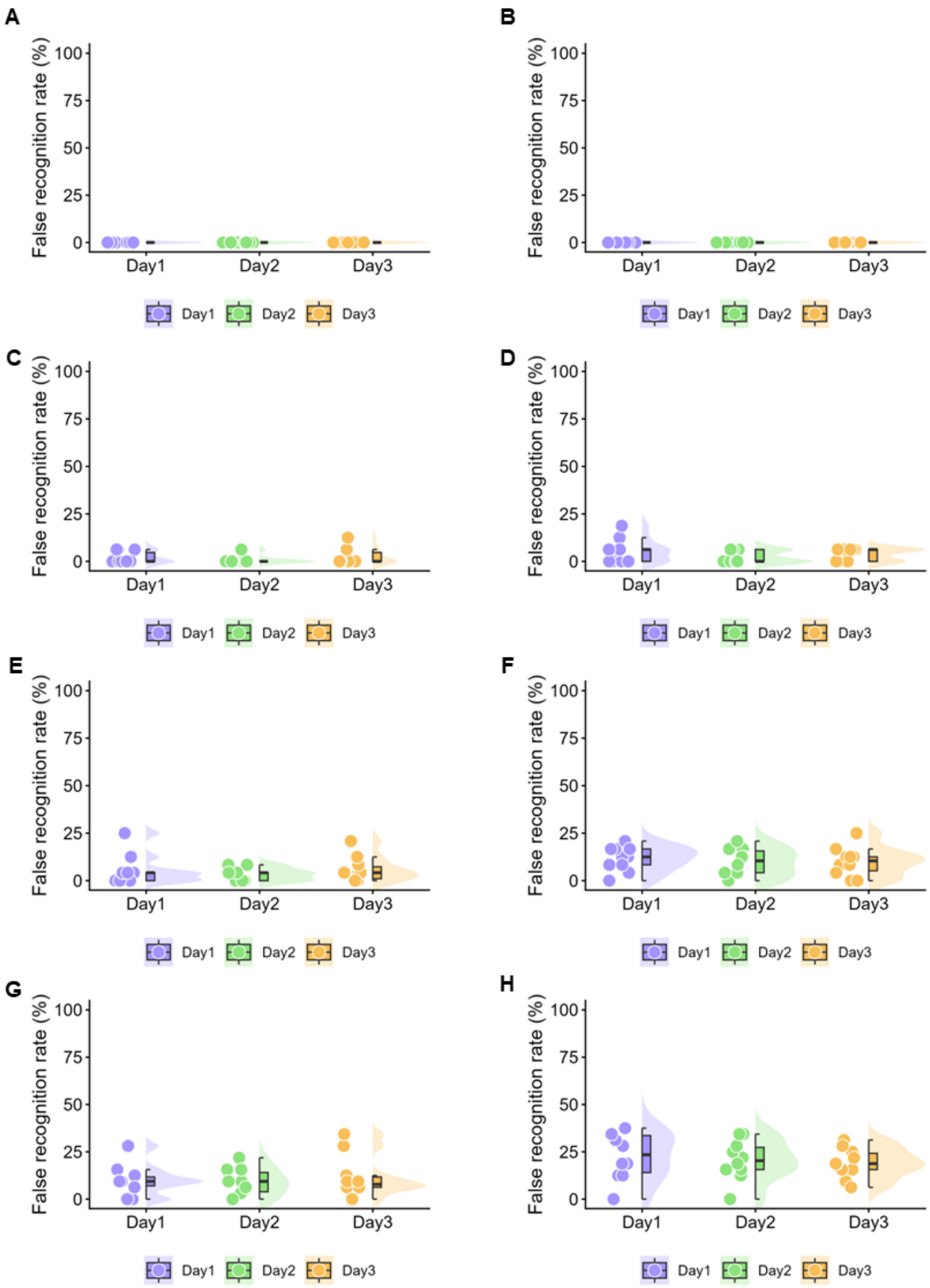

Figure S7. Distributions of false non-target recognition rates for the tactile-encoded

brain–computer interface (BCI) across increasing task complexity over three consecutive training days. Violin plots show participant-level false non-target recognition rates with embedded boxplots. Rows correspond to increasing numbers of classifiable targets: (A, B) one target, (C, D) two targets, (E, F) three targets, and (G, H) four targets. Columns represent task conditions, with single-task (BCI-only) performance shown in the first column and dual-task performance (BCI combined with ball-balancing) shown in the second.

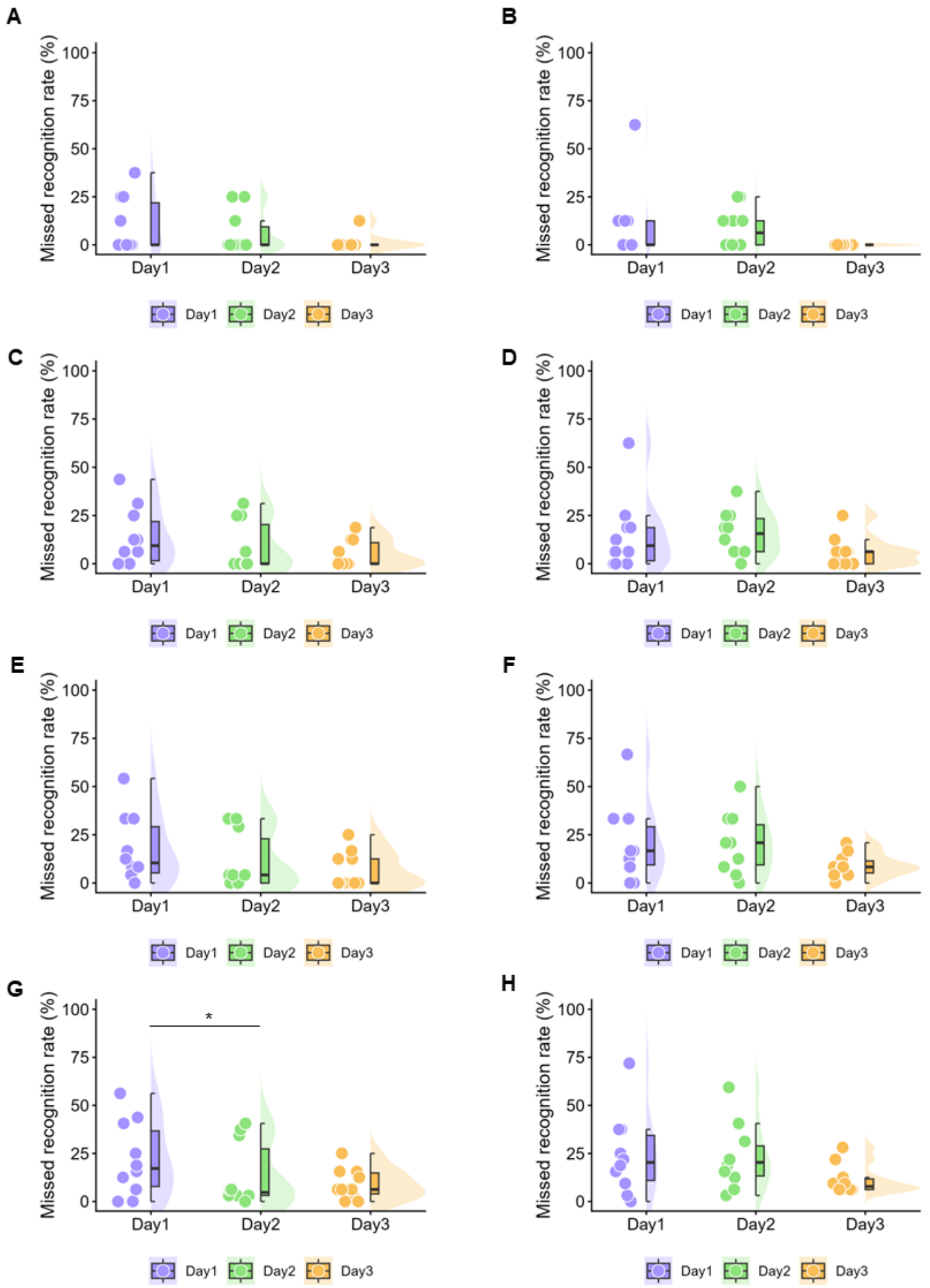

Figure S8. Distributions of missed-recognition rates for the tactile-encoded brain–computer interface (BCI) across increasing task complexity over three consecutive training days. Violin plots show participant-level missed-recognition rates with embedded boxplots. Rows correspond to increasing numbers of classifiable targets: (A, B) one target,

(C, D) two targets, (E, F) three targets, and (G, H) four targets. Columns represent task conditions, with single-task (BCI-only) performance shown in the first column and dual-task performance (BCI combined with ball-balancing) shown in the second (* if $p < 0.05$; one-way repeated-measures ANOVA).

The Reviewer's Comment #29

Why only four out of ten participants performed the additional test for continuous decoding? Why not everyone?

Response:

Thank you for your question. We acknowledge that the ideal presentation of results would have involved all participants completing the continuous decoding experiment. However, during our experiment, each participant was required to complete both the single-task and dual-task calibration sessions as well as online testing sessions for each day. The total duration per day was already considerable, and all participants were additionally required to repeat the experiment over three days. Therefore, for this additional test, we fully respected the participants' willingness to take part.

When initially drafting the manuscript, we discussed whether to include the results of this additional experiment. After discussion among all authors, we decided to present as many results as possible to demonstrate the application scenarios of this novel tactile-encoded BCI. Moreover, in Supplementary Material Movie S4, we have also provided a demonstration of continuous decoding, and the results from these four participants can serve to support this demonstration. Continuous decoding represents the next critical step in our ongoing investigation of the proposed tactile-encoded BCI, with the ultimate goal of integrating it with wearable robotic control to enable continuous control in three-dimensional space. We look forward to presenting this next phase of our research to you and readers in the near future.

The Reviewer's Comment #30

What was the maximum number of attempts? Since there could be 80 trials per run and one round consisted of 4 trials, there could be as many as 20 rounds. Since the first attempt began after the 4th round, the maximum number of attempts would be 17? Please indicate it.

Response:

Thank you for pointing out this concern. As we clarified in the previous response, to ensure the complete capture of the P300 waveform (considering that P300 latency can exceed 300 ms), we employed an overlapping epoch strategy. Specifically, to secure sufficient data length for the 16th trial in the first recognition attempt, the system was configured to delay the first classification until after the completion of the 18th trial. This technical setup has a direct implication for the maximum number of attempts: it results in the final round (trials 77-80) not being processed for a classification decision. Consequently, the recognition

attempts are made from the 4th round to the 19th round, yielding a total of 16 recognition attempts per run. To make this clear in the manuscript, we revised the descriptions in the Materials and Methods section in the revised manuscript.

"Given that the P300 response typically requires trial averaging to achieve a detectable peak, a sliding-window approach was adopted. EEG responses evoked by four consecutive stimuli with the same vibrator were averaged and fed into the decoder. Each stimulation round consisted of four distinct vibration stimuli (one stimulus per vibrator), delivered in a pseudorandom sequence, with a total duration of 1.6 seconds per round. Due to the requirement of trial averaging, the first recognition attempt occurred after the completion of the 18th trial. Thereafter, a decoding decision was made after each set of 4 consecutive trials, resulting in a total of 16 attempts per run."

The Reviewer's Comment #31

What was the average time interval between experimental sessions (days)?

Response:

Thank you for your question. We have added a detailed summary of timing information to the revised Supplementary Materials. The three experimental sessions (Day 1, Day 2, and Day 3) were completed within a 15-day window for all participants. Using Day 1 as the baseline, the individual time intervals for Day 2 and Day 3 are presented in Table S1. Overall, the second session occurred on average 4.2 ± 3.0 days after the first, and the third session occurred 8.5 ± 4.4 days after the first. The average time interval between experimental sessions is 4.3 ± 2.8 days. All participants completed the study within 15 days.

Table S1. Time intervals between experimental sessions for each participant, expressed in days relative to Day 1.

Participant	Day 2 (+ days)	Day 3 (+ days)
1	+2	+10
2	+7	+13
3	+1	+3
4	+4	+5
5	+8	+9
6	+7	+14
7	+8	+15
8	+1	+7
9	+1	+3
10	+3	+6
mean $\pm$ std	$+4.2 \pm 3.0$	$+8.5 \pm 4.4$

The Reviewer's Comment #32

Line 520-522. Channel Cz was chosen for the P300 analysis. Then, is it necessary to show the topographies in Fig. 2A, D?

Response:

We thank the reviewer for raising this point. After careful discussion, we hold the view that retaining these topographies remains beneficial. Although we ultimately selected channel Cz for further analysis, this decision was made after analysing the topographic maps, which revealed that Cz was generally the most responsive channel in terms of P300 presentation. This approach follows common practice in EEG studies, where brain topographies are first presented to identify representative electrodes, followed by detailed time-frequency or waveform plots of those selected channels. For instance, studies on movement-related cortical activity typically report statistical results from the C3 and C4 electrodes, such as Zhong et al. (2022), and Shu et al. (2018) Therefore, the primary value of these two topographies in Fig 2 is to provide a visual justification for our selection of the Cz channel. This supports the neurophysiological analysis of focusing our statistical analysis on Cz channel. Additionally, as you also suggested regarding the analysis of more channels, these brain topographies serve as a presentation of the results regarding this question. Therefore, we have retained the two brain topographies in this revised version.

However, your suggestion also prompted us to reexamine the topography in Fig. 2A and 2D, and we have identified that this figure may cause misunderstanding. The largest P300 amplitude appears to be at the prefrontal area. The apparent maximum P300 amplitude at the frontal electrodes is likely an artefact of interpolation after these channels were excluded from the analysis, which makes the prefrontal region appear artificially prominent. To address this, we replotted the topographies including Fz and F1–F7 channels. Thank you for highlighting this issue.

References:

Zhong, Yucun, et al. "Tactile sensation assisted motor imagery training for enhanced BCI performance: A randomized controlled study." IEEE Transactions on Biomedical Engineering 70.2 (2022): 694-702.

Shu, Xiaokang, et al. "Tactile stimulation improves sensorimotor rhythm-based BCI performance in stroke patients." IEEE transactions on biomedical engineering 66.7 (2018): 1987-1995.

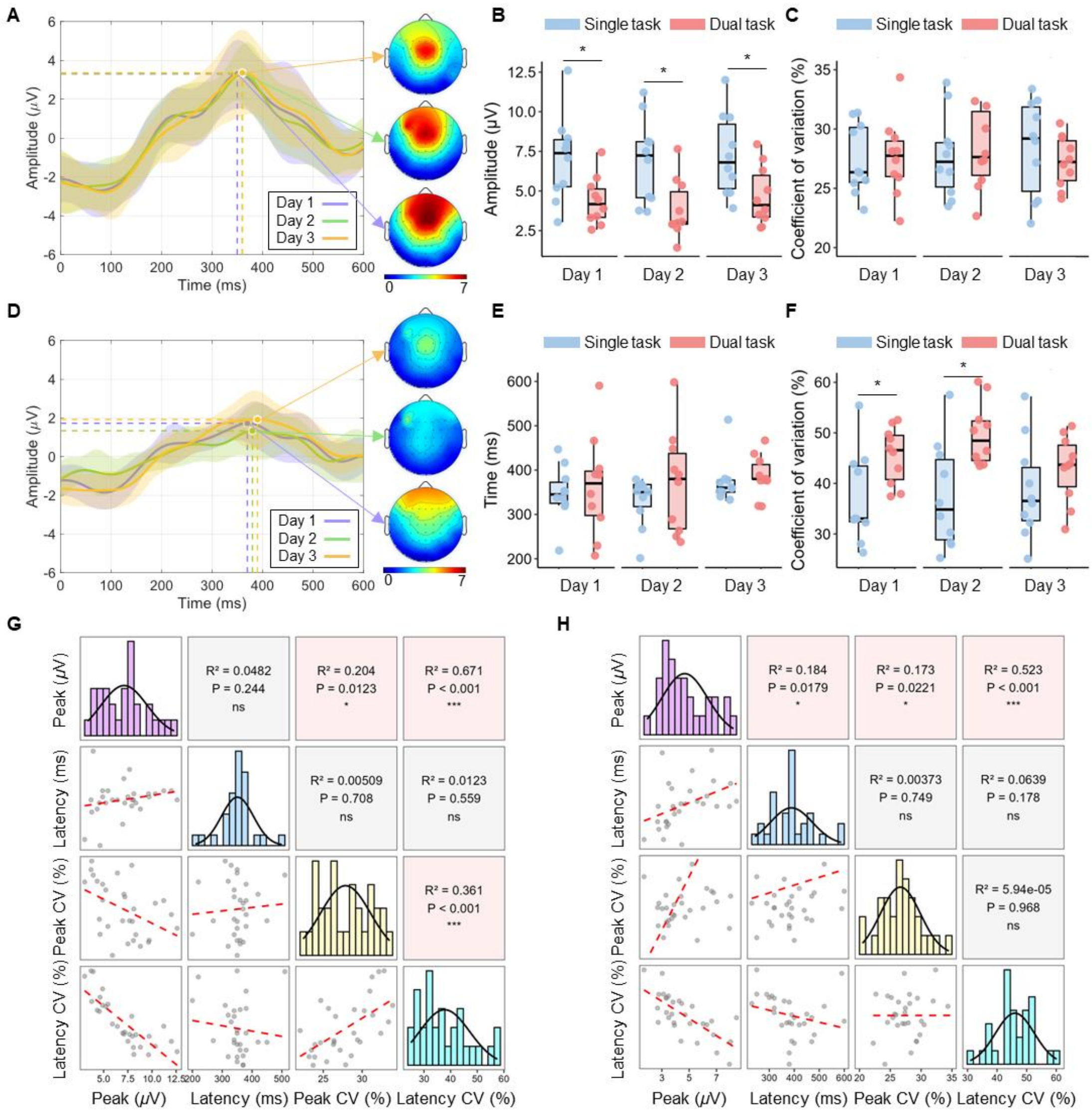

Fig. 2. Evoked P300 underlying the tactile-encoded BCI tasks over three days of experiment across 10 participants. Grand-averaged P300 of Cz for **(A)** single-task and **(D)** dual-task across days 1-3, with corresponding topographic maps illustrating the P300 peak distribution. The solid lines represent the mean amplitude of the P300, and the shaded boundary represents the standard deviation across participants. Boxplots of P300 **(B)** peak amplitude, **(C)** peak coefficient of variation (CV), **(E)** latency, and **(F)** latency CV across days. Correlation matrices of P300 features for **(G)** single-task and **(H)** dual-task conditions, with each dot representing individual data points from each participant per day. With an outlier of the peak CV omitted from the figure but included in statistical analysis. (*if $p < 0.05$).

The Reviewer's Comment #33

18 EEG channels in the frontal regions were excluded from the P300 analysis. Were these channels also excluded from decoding? But, in Fig. 2A, D, frontal channels showed the largest P300 amplitudes. So, these channels needed to be included in decoding. Please clarify which channels were excluded from the P300 analysis and decoding, respectively.

Response:

Thank you for raising this important point. EEG signals can be substantially influenced by eye blink and muscular artifacts, particularly over prefrontal regions, which can potentially lead to biases in the BCI control. Initially, in our offline analysis, we excluded all electrodes located over the frontal regions. Regarding your query concerning Fig. 2A and 2D, where the frontal channels appeared to show the largest P300 amplitudes: although the frontal channels were excluded from the analysis, the brain topography plots were generated using automatic interpolation. This interpolation likely resulted in the prefrontal regions visually displaying the highest amplitudes. To address this concern, we plotted Figures R2 and R3 to show the EEG P300 of all electrodes. It can be observed that the amplitude standard deviation of the P300 recorded from electrodes over the prefrontal region is very large, indicating severe susceptibility to noise. Consequently, in our online BCI control tests, the first two rows of electrodes located over the frontal regions were entirely excluded. These excluded electrodes include: Fp1, Fp2, AF7, AF3, AFz, AF4, and AF8. Additionally, the two outermost temporal electrodes, FT9 and FT10, were also excluded from both online and offline analyses. Regarding the third-row electrodes (Fz and F1–F7), these were found to be less affected by these artifacts. Notably, during eye blinks, no significant artifacts were observed in these channels. Furthermore, as the P300 features are predominantly below 10 Hz, we applied a 10 Hz low-pass filter to the EEG signals in both online testing and offline analysis to minimize the influence of such artifacts as much as possible. Therefore, in this round of revision, electrodes Fz and F1–F7 were included in the analysis, as these electrodes were also used for online decoding during the experiment. In this revised version, we have included electrodes Fz and F1–F7 in the offline analysis and have subsequently replotted Fig. 2A and 2D accordingly. After incorporating these channels, the results remained consistent with our previous conclusions: electrode Cz continued to exhibit the largest P300 amplitude across all ten participants during the first calibration phase on day 1, thereby serving as a baseline. We have clarified this point in the revised manuscript. We agree with you that the initial description of the 18 excluded channels was too general. We have therefore provided a more specific description in the revised manuscript.

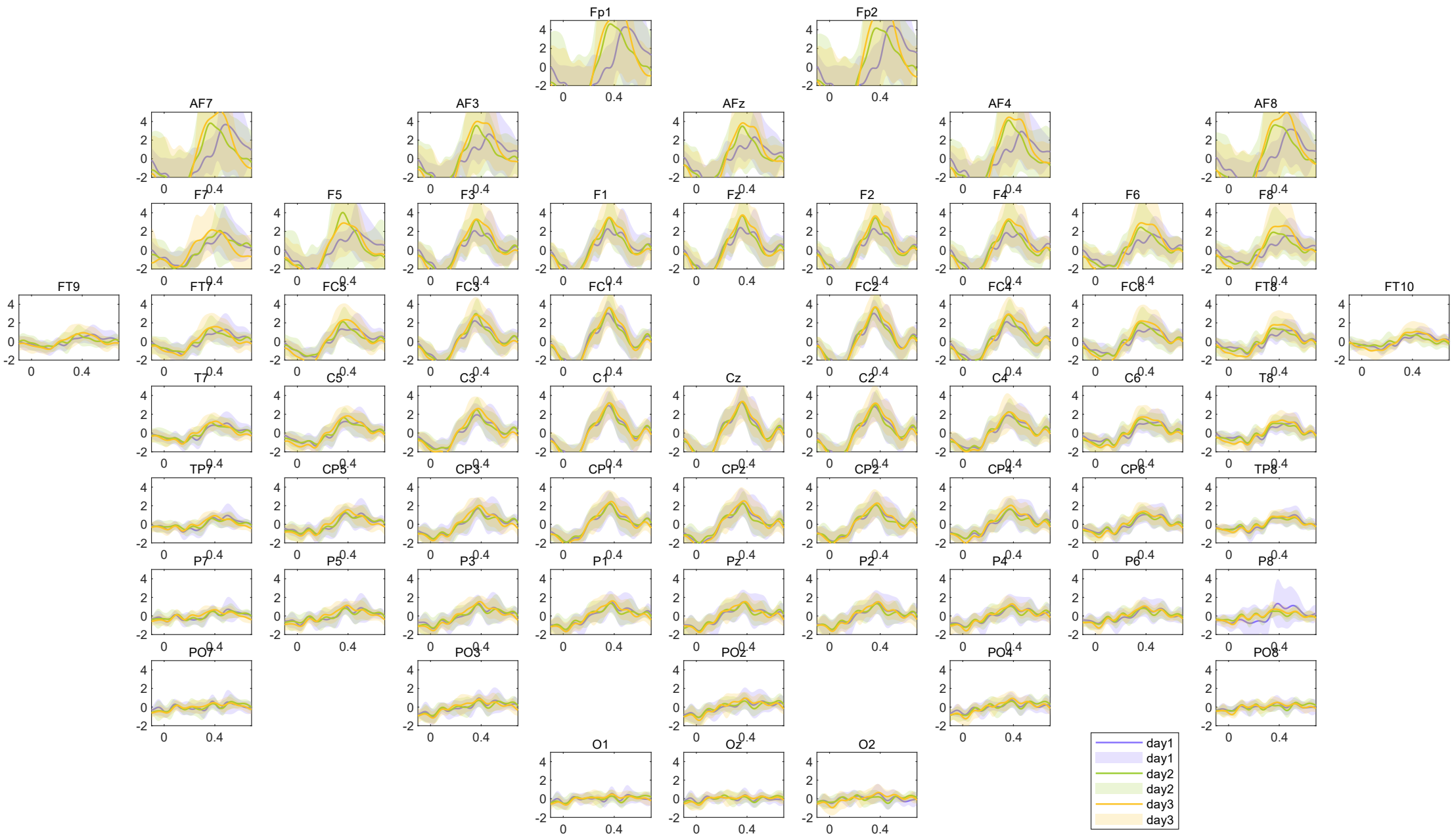

Figure R2. Evoked P300 underlying the tactile-encoded BCI tasks over three days of experiment across 10 participants for single-task.

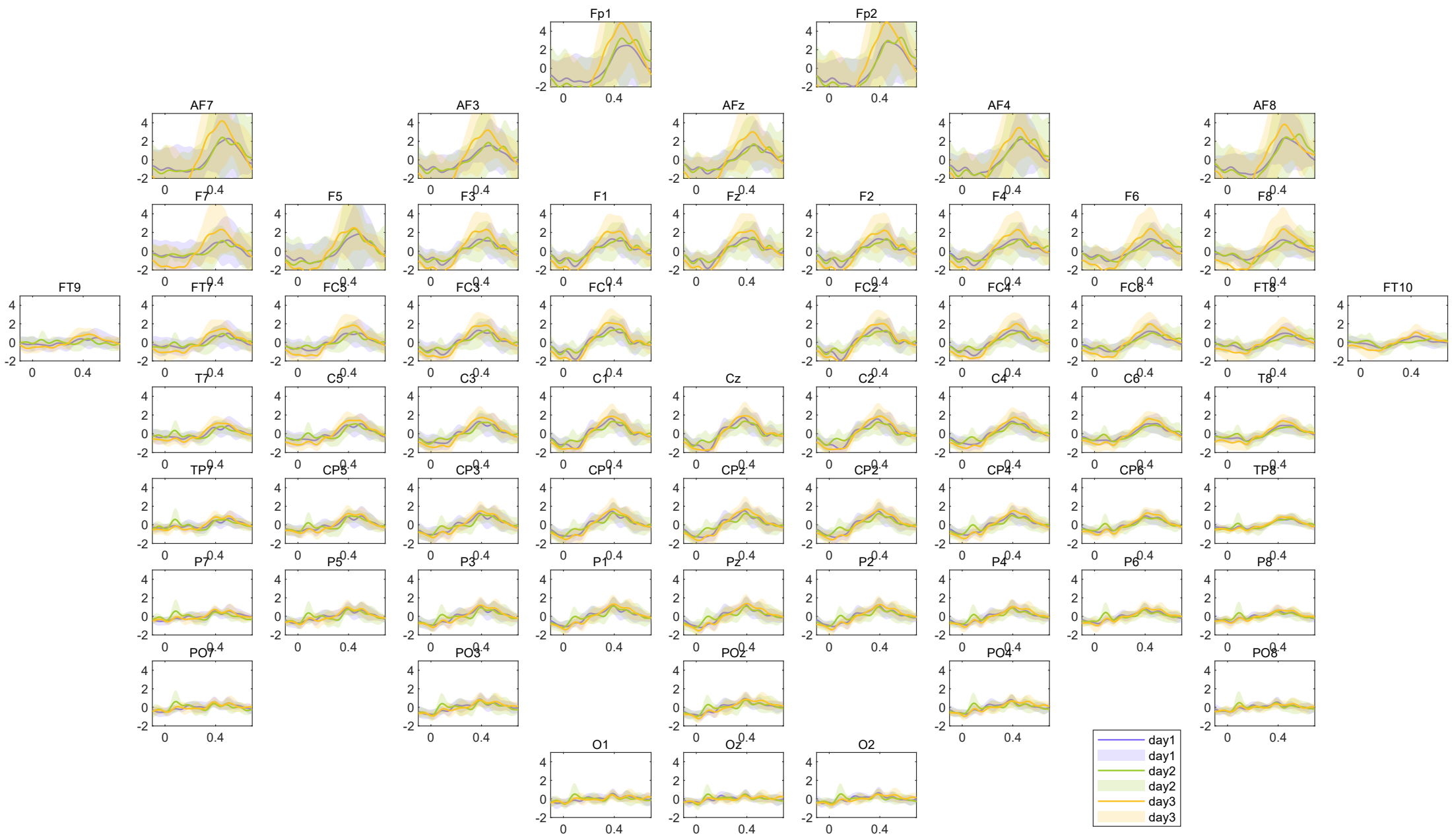

Figure R3. Evoked P300 underlying the tactile-encoded BCI tasks over three days of experiment across 10 participants for dual-task.

"***EEG pre-processing:*** EEG from each run was pre-processed using a standard sequence. Data were epoched from -0.1 to 0.7s relative to stimulus onset. A finite impulse response band-pass filter (1-10 Hz) and a notch filter (48-52 Hz) were applied to remove slow drifts and high-frequency noise. The signals were then re-referenced to the average of electrodes M1 and M2, placed on the mastoid processes, to reduce reference-dependent artefacts. After baseline removal, data were downsampled to 100 Hz. For each trial, the post-stimulus interval of 100-500 ms was extracted, as the P300 peaks typically appear

around 300 ms (21). This window emphasises the positive deflection while excluding later potentials. Due to substantial contamination from eye blinks and facial muscle artefacts, 7 EEG channels over the frontal regions (Fp1, Fp2, AF7, AF3, AFz, AF4, and AF8) and 2 two temporal electrodes (FT9, FT10) were excluded from the P300 analysis. M1, M2, and CPz, which are commonly used as references in EEG analysis, were also excluded from analysis. The remaining electrodes were all included for offline analysis, whereas those exhibiting observable P300 responses were used for online decoding."

The Reviewer's Comment #34
It is recommended to report the performance evaluation results for the four-limb coordination tasks, even though these tasks are designed for the demonstration purpose.

Response:
We sincerely thank you for your valuable suggestion. A total of four demonstration movies were provided. To objectively and quantitatively evaluate the performance of the four-limb coordination tasks, we proposed three key metrics. In practical BCI control tasks, it is expected that the next command should be triggered as soon as possible after the completion of the preceding command. Therefore, based on the raw data of the four demonstrations and the recorded videos, we calculated the command-switch intervals. Considering that in actual control, the subject typically waited until the robotic arm movement task triggered by the previous command is completed before triggering the next command, we also provided the command-switch intervals after excluding the robotic arm movement duration. Furthermore, for continuous control tasks, it is desirable that movement ceases immediately upon reaching the target position. Hence, the command-stop delay was also adopted as an evaluation metric. These results are presented in Table R2: Performance Evaluation for the Demonstrations, which has been added to the revised Supplementary Material.

"

Table R2. Performance evaluation for the demonstrations.

Demonstration No.	Metrics	C1→C2	C2→C3
Demo 1	[1]Command-switch interval 1 (s)	11.2	N/A
	[2]Command-switch interval 2 (s)	8.2	N/A
	[3]Command-stop delay (s)	1.6	N/A
Demo 2	[1]Command-switch interval 1 (s)	16	27.2
	[2]Command-switch interval 2 (s)	8.3	12.6
	[3]Command-stop delay (s)	1.6	1.6
Demo 3	[1]Command-switch interval 1 (s)	12.8	11.2
	[2]Command-switch interval 2 (s)	4.8	5.6
	[3]Command-stop delay (s)	1.6	9.6
Demo 4	[1]Command-switch interval 1 (s)	3.2	4.8
	[2]Command-switch interval 2 (s)	2.1	3.7
	[3]Command-stop delay (s)	1.6	0

[1]Command-switch interval 1 refers to the interval from the first recognition of the previous

command to the first recognition of the next command.
[2]Command-switch interval 2 is defined as Command-switch interval 1 minus the time taken by the robotic arm to execute the task.
[3]Command-stop delay refers to the duration of the delay before the recognition of the previous command concludes.
"

The Reviewer's Comment #35
Reporting the BCI performance in terms of the information transfer rate (ITR) would be helpful to compare the performance with existing tactile P300 BCIs.

Response:
Following your recommendation, we have added the information transfer rate (ITR) (Figure S10) to the revised Supplementary Materials. In this study, ITR was computed based on the four-degree-of-freedom online recognition success rates reported in Figure 3A and B, together with the corresponding recognition time (calculated based on recognition attempt reported in Figure 3D and E). We would like to highlight an important point regarding the ITR calculation: we used the online success rate and the classification accuracy respectively as the basis for computing ITR. This is because the online task involves the recognition of four degrees of freedom, and while the theoretical classification accuracy must be higher, the success rate more faithfully reflects the functional performance of the system in a real-time control context. However, to ensure consistency and mutual validation across all reported results, we also deliberately adopted the online success rate for ITR estimation.

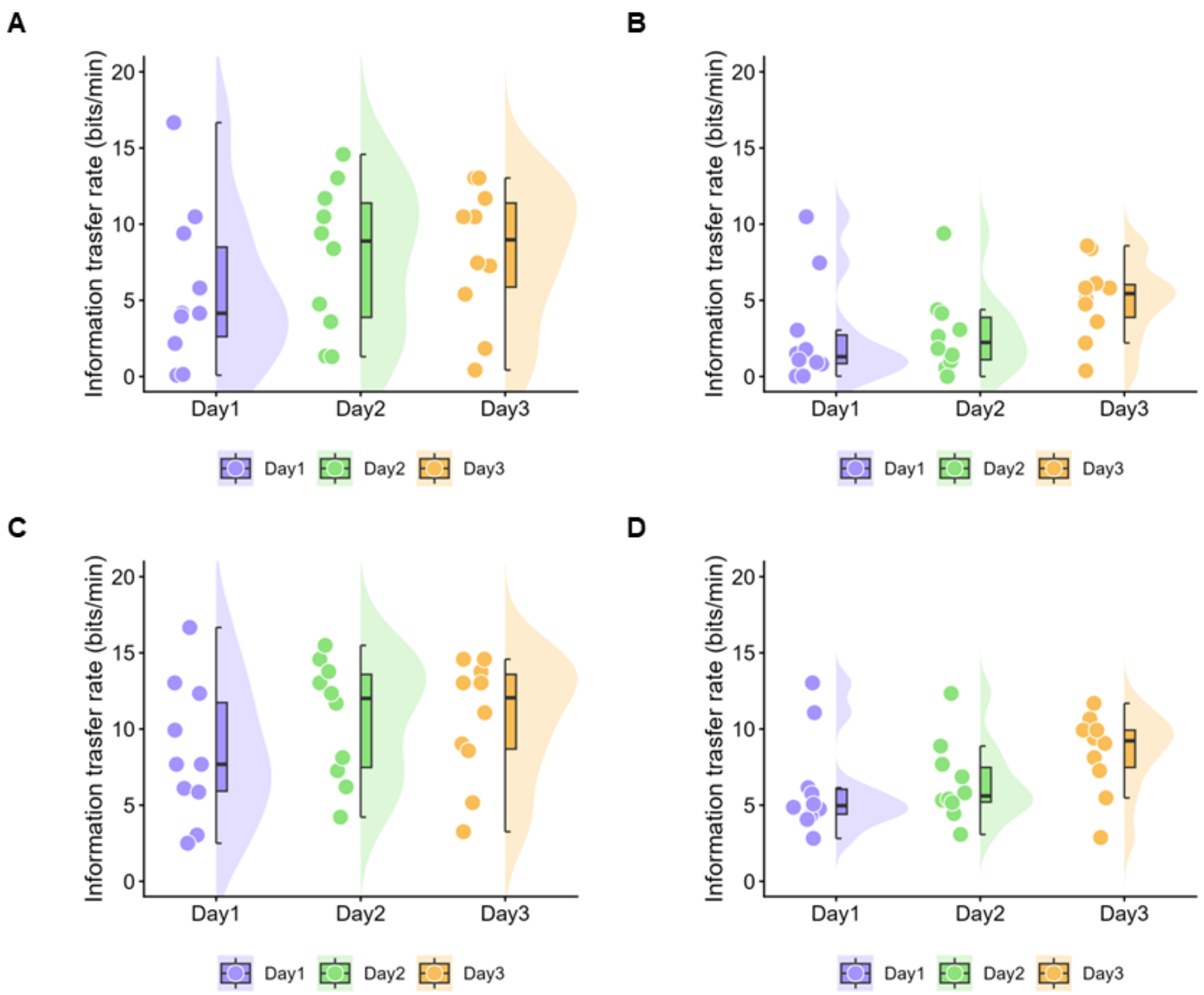

Figure S10. Distributions of information transfer rate (ITR) for the tactile-encoded brain–computer interface over three training days. Violin plots show participant-level ITR with embedded boxplots. ITR was calculated based on online success rate in (A) single-task and (B) dual-task conditions, and based on accuracy in (C) single-task and (D) dual-task conditions.

Response to Reviewers' Comments

Nature Communications

Manuscript ID: NCOMMS-26-004391A

Concurrent control of natural and robotic limbs through a tactile-encoded brain-computer interface

Tianyu Jia, Xingchen Yang, Ciaran McGeady, Yifeng Li, Jinzhi Lin, Kit San Ho, Feiyu Pan, Linhong Ji, Chong Li, Dario Farina

To Editors and Reviewers

We sincerely appreciate your time and effort dedicated to reviewing our manuscript. We greatly appreciate the constructive feedback and the insightful comments, and we are delighted that the revisions we made have satisfactorily addressed your concerns, which have helped us improve our work. Regarding the three minor concerns raised by Reviewer 4, our point-by-point responses are listed below. We look very much forward to seeing our work published in *Nature Communications*.

Reviewer #4

The Reviewer's Comment #1

I truly appreciate the authors' tremendous efforts in revising the manuscript. I believe the manuscript has now improved significantly. While the authors have thoroughly and satisfactorily addressed all the comments I raised, I would like to suggest a few minor additional points as follows.

Regarding the sense of embodiment, I fully understand that recall bias can affect the results of the survey conducted six months after the original experiment and that it would therefore not be appropriate to include these data in the manuscript. However, as the sense of embodiment may present an important question in the context of movement augmentation, I would suggest mentioning it as a topic for future investigation in the Discussion, so that readers are made aware of this open issue.

Response:

We agree that incorporating an assessment of the sense of embodiment is an important question in the context of movement augmentation. We have added a description of this aspect in the Discussion section, hopefully this will provide more insights for readers.

"Furthermore, the enhancement of the sense of embodiment may also contribute to performance gains. In future studies, this could be investigated by questionnaires to assess whether users could develop a sense of embodiment and improve it with training."

The Reviewer's Comment #2

Since Figures R2 and R3 are indeed informative regarding the ERP responses underlying the proposed BCI, I would suggest including them in the Supplementary Materials.

Response:

Thank you for your suggestion. We have added Figures R2 and R3 to the Supplementary Materials, where they are named as Figures S14 and S15.

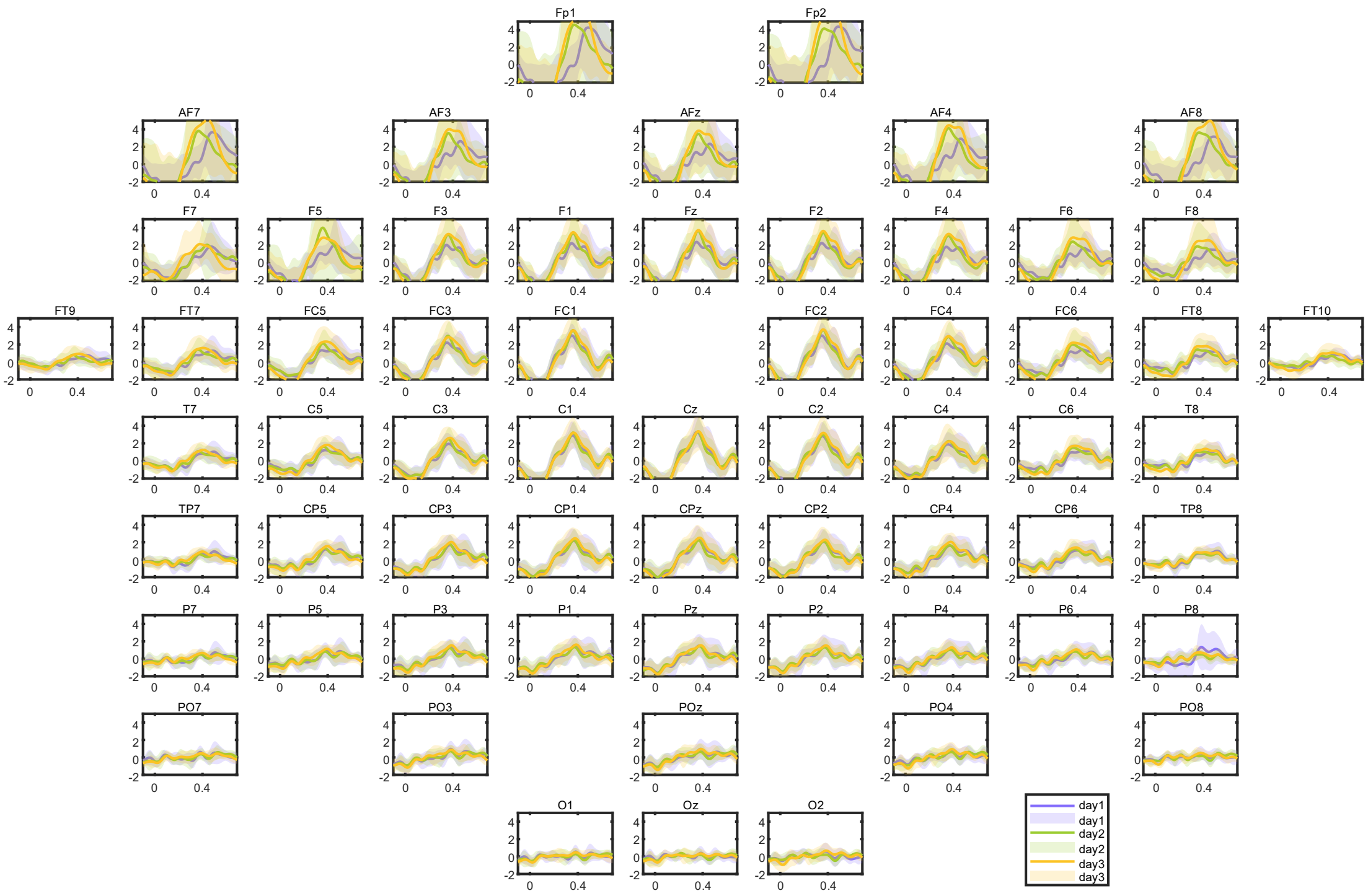

Figure S14. Evoked P300 underlying the tactile-encoded BCI tasks over three days of experiment across 10 participants for the single-task condition. The solid lines represent the mean amplitude of the P300, and the shaded boundary represents the standard deviation across participants.

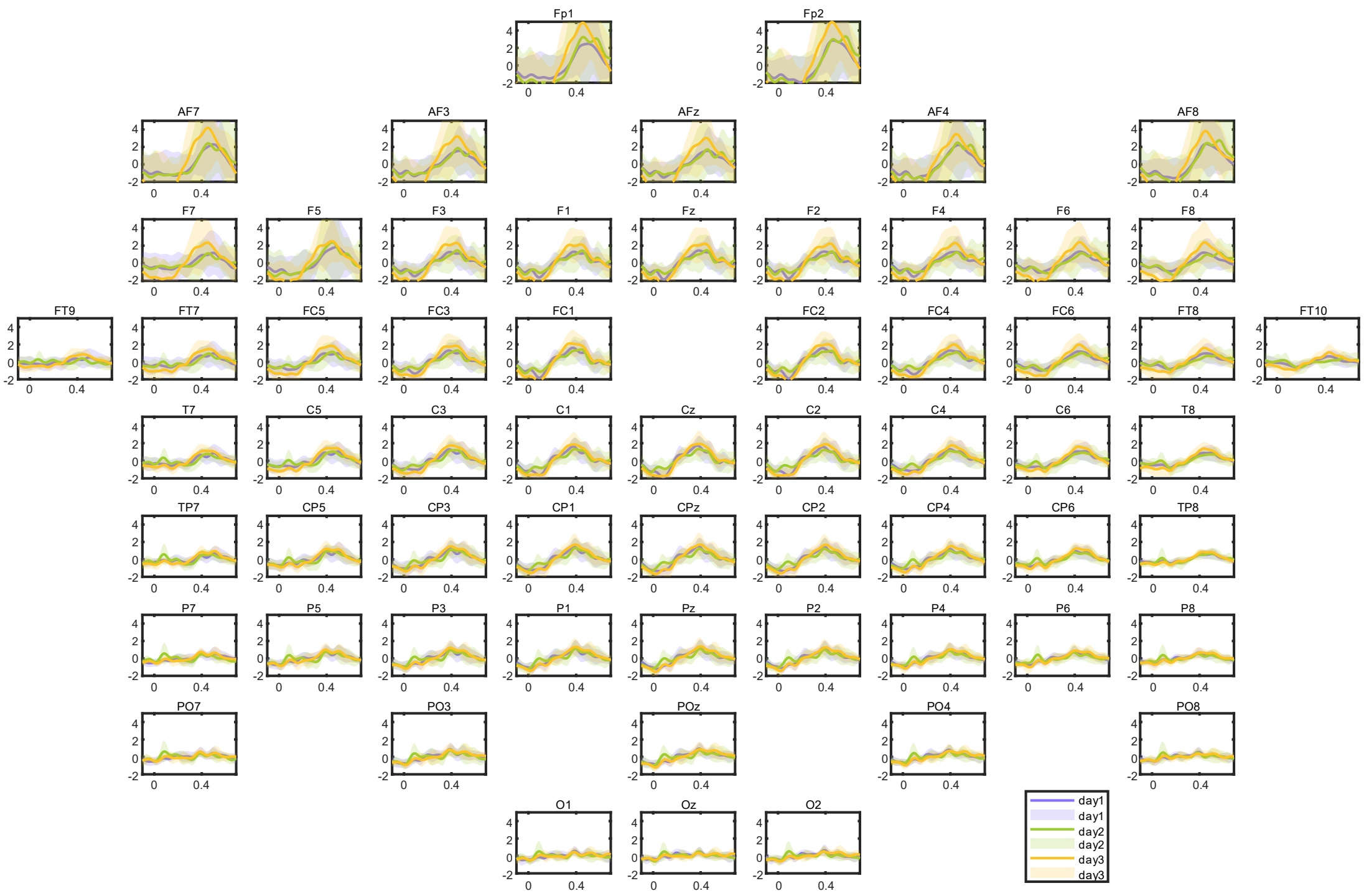

Figure S15. Evoked P300 underlying the tactile-encoded BCI tasks over three days of experiment across 10 participants for the dual-task condition. The solid lines represent the mean amplitude of the P300, and the shaded boundary represents the standard deviation across participants.

The Reviewer's Comment #3

Regarding the mapping between stimuli and commands, it is now clear that the mapping was kept consistent across participants, which is entirely appropriate. However, I still do not fully understand how keeping the consistent mapping across participants "facilitates learning across sessions". It is clear that keeping a certain mapping within a given participant across sessions would facilitate learning for that individual. What remains unclear is why keeping a consistent mapping across participants would facilitate learning across sessions within each participant.

Response:

We fully agree with this point. Accordingly, we have removed the phrase "to facilitate learning across sessions" from the manuscript. Thank you again for your suggestion and careful review.

"This mapping was kept consistent across participants, while the presentation order of the vibrotactile stimuli within each run was pseudorandomized according to the oddball paradigm."
